# A core microbiota dominates a rich microbial diversity in the bovine udder and may indicate presence of dysbiosis

**DOI:** 10.1038/s41598-020-77054-6

**Published:** 2020-12-10

**Authors:** Davide Porcellato, Roger Meisal, Alberto Bombelli, Judith A. Narvhus

**Affiliations:** 1 Faculty of Chemistry, Biotechnology and Food Science, The Norwegian University of Life Sciences, P.O. Box 5003, 1432 Ås, Norway; 2 Present Address: Møreforsking Ålesund AS, Ålesund, Norway

**Keywords:** Microbial communities, Microbial ecology

## Abstract

The importance of the microbiome for bovine udder health is not well explored and most of the knowledge originates from research on mastitis. Better understanding of the microbial diversity inside the healthy udder of lactating cows might help to reduce mastitis, use of antibiotics and improve animal welfare. In this study, we investigated the microbial diversity of over 400 quarter milk samples from 60 cows sampled from two farms and on two different occasions during the same lactation period. Microbiota analysis was performed using amplicon sequencing of the 16S rRNA gene and over 1000 isolates were identified using MALDI-TOF MS. We detected a high abundance of two bacterial families, *Corynebacteriaceae* and *Staphylococcaceae,* which accounted for almost 50% of the udder microbiota of healthy cows and were detected in all the cow udders and in more than 98% of quarter milk samples. A strong negative correlation between these bacterial families was detected indicating a possible competition. The overall composition of the udder microbiota was highly diverse and significantly different between cows and between quarter milk samples from the same cow. Furthermore, we introduced a novel definition of a dysbiotic quarter at individual cow level, by analyzing the milk microbiota, and a high frequency of dysbiotic quarter samples were detected distributed among the farms and the samples. These results emphasize the importance of deepening the studies of the bovine udder microbiome to elucidate its role in udder health.

## Introduction

There is still an ongoing discussion as to whether the mammary internal epithelial surface of the bovine udder harbours its own microbiota and whether this microbiota has a role in influencing udder health^1,2^. The source of microorganisms in the milk found deep inside the udder may originate from external contamination of the teat area followed by migration into the remote alveoli. Alternatively, translocation may occur from other parts of the body, such as the gut, via the blood vessels^3^. It has been hypothesized that translocation of bacteria from the rumen to the udder (and into the milk) may be important for the development of the intestinal microbiota of the suckling calf^4^.

The healthy bovine udder is a large organ comprised of four milk-producing quarters which each open to the outside of the body through a teat canal. Milk production takes place in the alveoli and collects first in the gland cistern and then in the teat cistern. In healthy udders, the quarters produce copious amounts of milk with low numbers of somatic cells and the numbers of microorganisms are sparse. Bovine mastitis causes considerable animal suffering and enormous economic losses on a global scale^5^. Mastitis leads to an increase in the numbers of somatic cells especially leukocytes in the milk, which may herald the infection before other clinical signs appear and also large numbers of the infecting organisms as well as a reduced milk yield. During mastitis, an imbalanced microbiota is detected in the infected quarter milk samples and this is caused by the presence of the pathogenic microorganism, the immune response of the cow and the interactions between the pathogen and the resilient microbiota^6,7^.

More recent studies have used molecular techniques to study the microbiology of milk from the udder and have uncovered an extraordinary diversity of bacteria. However, Taponen et al.^2^ summarized several previous microbiota studies and showed that the results are so variable that an identification of a generic udder microbiome is not at present possible. They also explained how a variety of factors would affect the results, especially the method of sampling. The studies reviewed by Taponen et al.^2^ were carried out using a variety of analysis methods and sampling techniques and, in addition, milk was obtained from different breeds of cow. Milk sampling methodology and microbiological analysis was recently studied by Dahlberg et al.^8^. They concluded that the contribution of external contamination of samples was a considerable problem when the bacterial load of the milk was low and this, in addition, made the removal of kit contaminations a challenge.

The presence of a healthy microbiota in diverse human body sites has been linked to a protective role against dysbiosis-related diseases such as inflammatory bowel disease or mastitis^9,10^.In cattle, there is evidence that mastitis changes the microbiome composition of the udder and that the functionality of the microbiome differs^11,12^. Furthermore, the microbiome balance within the quarter seems to be very fragile and is subject to several shaping factors from the host and from the environment (reviewed in^13^). More findings are needed to elucidate the role of the udder microbiome in the lactating cows and to identify its potential role against mastitis. Therefore, the aim of this work was to decipher the microbial composition at quarter level of cows regularly milked and with no sign of clinical mastitis in order to increase the knowledge of the potential microbial role. We performed a cross-sectional study starting with milk from 60 Norwegian Red cows selected from two farms and sampled at early and late lactation. Microbial composition was assessed using amplicon sequencing of the 16S rRNA gene and, in addition, we identified over 1000 isolates to confirm the presence of viable bacteria in the samples.

## Results

### Overall composition of the udder microbiota across farms and sampling periods

Quarter milk samples were collected from 60 cows in the first sampling period. Between the two sampling periods, eleven cows were removed from the study. All the cows were sampled during a single lactation period and the average days in milking (DIM) was 38 and 44 for farm A (“Centre for livestock production” at the Norwegian University of Life Sciences) and farm K (“Kalnes Upper Secondary School” farm), respectively in the first period and 213 and 222 during the second period. The range of parity of the cows was similar between the two farms (Table S1).

**Figure 1:**
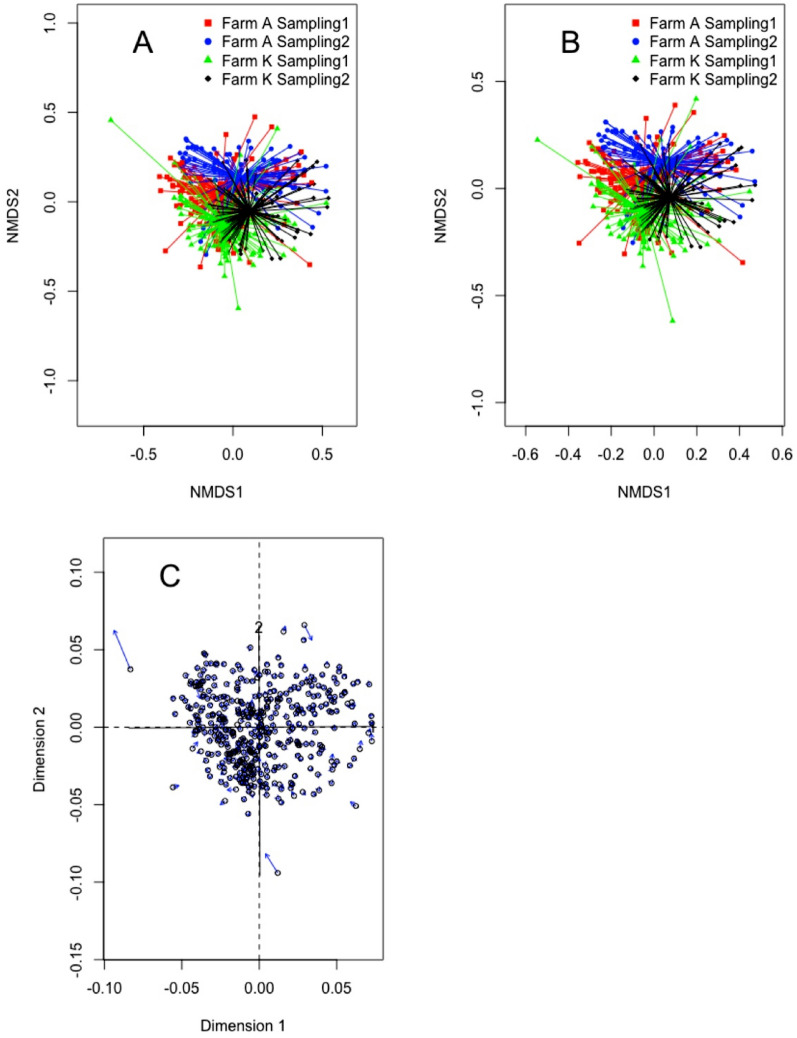
Non-metric multidimensional scaling (NMDS) plot of bovine quarter milk samples. (**A**) NMDS using Bray–Curtis dissimilarity matrix and (**B**) NMDS constructed using the Jaccard index dissimilarity matrix. (**C**) Procrustes analysis combining the NMDS results for Bray–Curtis and Jaccard index dissimilarity matrixes.

To explore the composition of the udder microbiota, amplicon sequencing of the 16S rRNA gene was performed for all the samples. From the original number of samples, a total of 403 quarter milk samples passed the quality filtering steps and were included in the study. The number of high-quality sequences obtained were 4,832,201 with an average of 12,549 sequences per milk sample (median 5988, min 109, max 102,760). A total of 10,010 sequence variants (SV) were detected and of these, 8759 were positively assigned to family level and kept for further analysis. Species richness estimation (Chao1) had an average of 89 SV per samples (median 61) and was significantly different (*p* < 0.05) between farm A (median 69.5) and farm K (median 58) but did not show significant difference between the two sampling periods. No significant differences were found with respect to sampling period and farms, regarding species diversity, which was calculated using Shannon index. Between-samples diversity (beta diversity) was calculated using non-metric multidimensional scaling using as input the Bray–Curtis and Jaccard distance matrix (Fig. 1). Both plots showed highly similar grouping between samples from the different farms and different sampling periods. This was also confirmed by Procrustes analysis (Fig. 1C). Multivariate homogeneity of group dispersion was not significant between the sampling periods or between the farms (*p* > 0.05) while PERMANOVA was significant (*p* < 0.001) between the two farms, between the two sampling occasions and the interaction between sampling and farms using both the Bray–Curtis and the Jaccard distance matrix.

**Figure 2:**
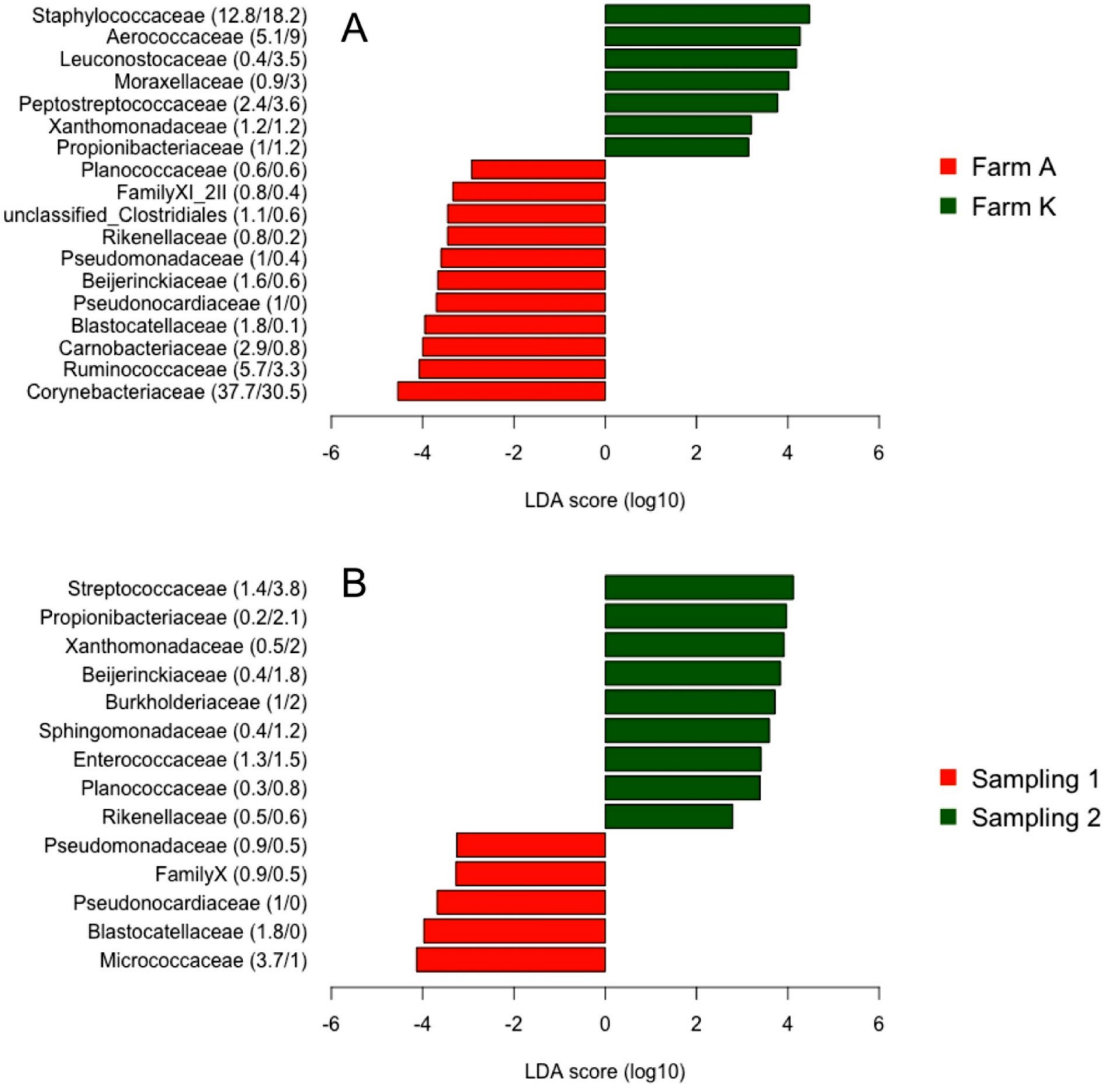
Linear discriminant analysis effect size (LEfSe) scores computed between the relative abundances of the most abundant families (> 0.5%) between (**A**) the farms and (**B**) the sampling periods. Values after taxa names are the percentage of abundance detected for each group.

Taxonomical evaluation of the bacterial SVs identified 75 orders and 186 families. Twenty-seven families had a relative abundance greater than 0.5%. *Corynebacteriaceae* dominated the microbiota and accounted for more than 1/3 of the reads (34.2%) followed by *Staphylococcaceae, Aerococcaceae* and *Ruminococcaceae* (Table 1). These four families were detected in more than 80% of the quarter milk samples from both farms. Linear discriminant analysis of the relative abundances identified 51 families which were significantly different between the farms. Forty-one families were significantly enriched in quarter milk samples from farm A and 18 families from samples obtained from the farm K. In particular, the two most abundant families in the experiment (*Corynebacteriaceae* and *Staphylococcaceae*) were those that differed the most between farms (Fig. 2). In particular, *Corynebacteriaceae* were more abundant in farm A (38.3%, vs. 31.1% in farm K), while *Staphylococcaceae* showed a greater abundance in samples from farm K (18.5%, vs. 13.1%). Sequence variants assigned to the *Corynebacteriaceae* family were found in almost all the quarter milk samples (99 and 98% of the samples from farm A and K, respectively), while *Staphylococcaceae* were present in 98% of the quarter milk samples from farm K and in 93.6% of the quarter from farm A. However, these two families were detected in all the 60 cows in both sampling periods. Among the *Corynebacteriaceae* sequences, *Corynebacterium bovis* was the dominant taxa detected. The sequences assigned to this species accounted for 47.3% of all the *Corynebacteriaceae* sequences and 74.9% of the quarter milk samples were positive for *C. bovis*. Among the most abundant SVs assigned to the family *Staphylococcaceae,* only one SV was identified as *Staph. aureus* (SV_53, Table S3) and accounted for the 1.2% of all the sequences assigned to this family. Most of the *Staphylococcaceae* sequences were mostly identified, by sequence search, to the minor mastitis-related species of non-aureus staphylococci (NAS) group. This group included *Staph. epidermidis, Staph. haemolyticus, Staph. simulans, Staph. chromogenes* and *Staph. xylosus.*

**Table 1:** Descriptive results of the microbiota summarized at family level (> 0.5%) detected in 403 quarter milk samples of 60 Norwegian Red cows.

	% Read over total	Number SV	% Quarter positive in farm A	% Quarter positive in farm B	% Positive cows	Average % of reads in farm A	Average % of reads in farm K
*Corynebacteriaceae*	34.2	742	99	98	100	38.3	31.1
*Staphylococcaceae*	15.5	254	93.6	98	100	13.1	18.5
*Aerococcaceae*	7	261	83.8	89.4	100	5.2	9.2
*Ruminococcaceae*	4.5	1518	83.8	85.9	100	6	3.4
*Peptostreptococcaceae*	3	107	76.5	79.9	100	2.5	3.7
*Streptococcaceae*	2.6	155	52.5	53.8	95	2.3	2.9
*Micrococcaceae*	2.4	118	68.6	57.3	98.3	2	3.1
*Lachnospiraceae*	2.4	739	75	73.4	100	2.7	2.3
*Moraxellaceae*	2	209	67.2	76.9	100	1	3.1
*Leuconostocaceae*	2	48	15.2	70.4	78.3	0.4	3.6
*Carnobacteriaceae*	1.8	135	71.1	59.3	100	2.9	0.8
*Burkholderiaceae*	1.4	237	66.2	52.8	96.7	1.3	1.7
*Enterococcaceae*	1.4	87	29.9	22.6	78.3	1.3	1.5
*Xanthomonadaceae*	1.2	59	49.5	28.6	85	1.2	1.2
*Beijerinckiaceae*	1.1	76	48.5	24.1	88.3	1.6	0.6
*Propionibacteriaceae*	1.1	62	47.1	53.8	95	1	1.2
*Blastocatellaceae*	1	19	17.2	5	30	1.9	0.1
Unclassified *Clostridiales*	0.9	503	68.6	54.3	98.3	1.2	0.6
*Sphingomonadaceae*	0.8	91	46.1	42.2	95	0.9	0.7
Family X	0.7	4	1.5	0	5	0	0
Family XI	0.7	6	67.6	58.8	98.3	1	0.5
Family XI_2	0.7	89	28.9	27.6	85	0.2	0.1
*Pseudomonadaceae*	0.7	150	52.9	30.2	96.7	1.1	0.4
*Planococcaceae*	0.6	72	43.1	32.7	88.3	0.6	0.6
Family XI_2II	0.6	182	60.3	46.7	96.7	0.8	0.4
*Rikenellaceae*	0.5	389	50	35.2	96.7	0.9	0.3
*Pseudonocardiaceae*	0.5	26	21.1	1.5	48.3	1	0

Interestingly, *Leuconostocaceae* which accounted for only 2% of the total microbiota, were found mostly in farm K. This family was found in 70% of the quarters from farm K but only in 15% of the quarters from farm A. A temporal shift of the udder microbiota could be identified between the two sampling periods. In total, 21 families were significantly more abundant during the second sampling (late Aug-Sep) and 13 were more abundant during the first sampling (Jan-Mar). In particular, *Streptococcaceae* and *Micrococcaceae* were the two families that differed the most between the two sampling periods.

**Figure 3:**
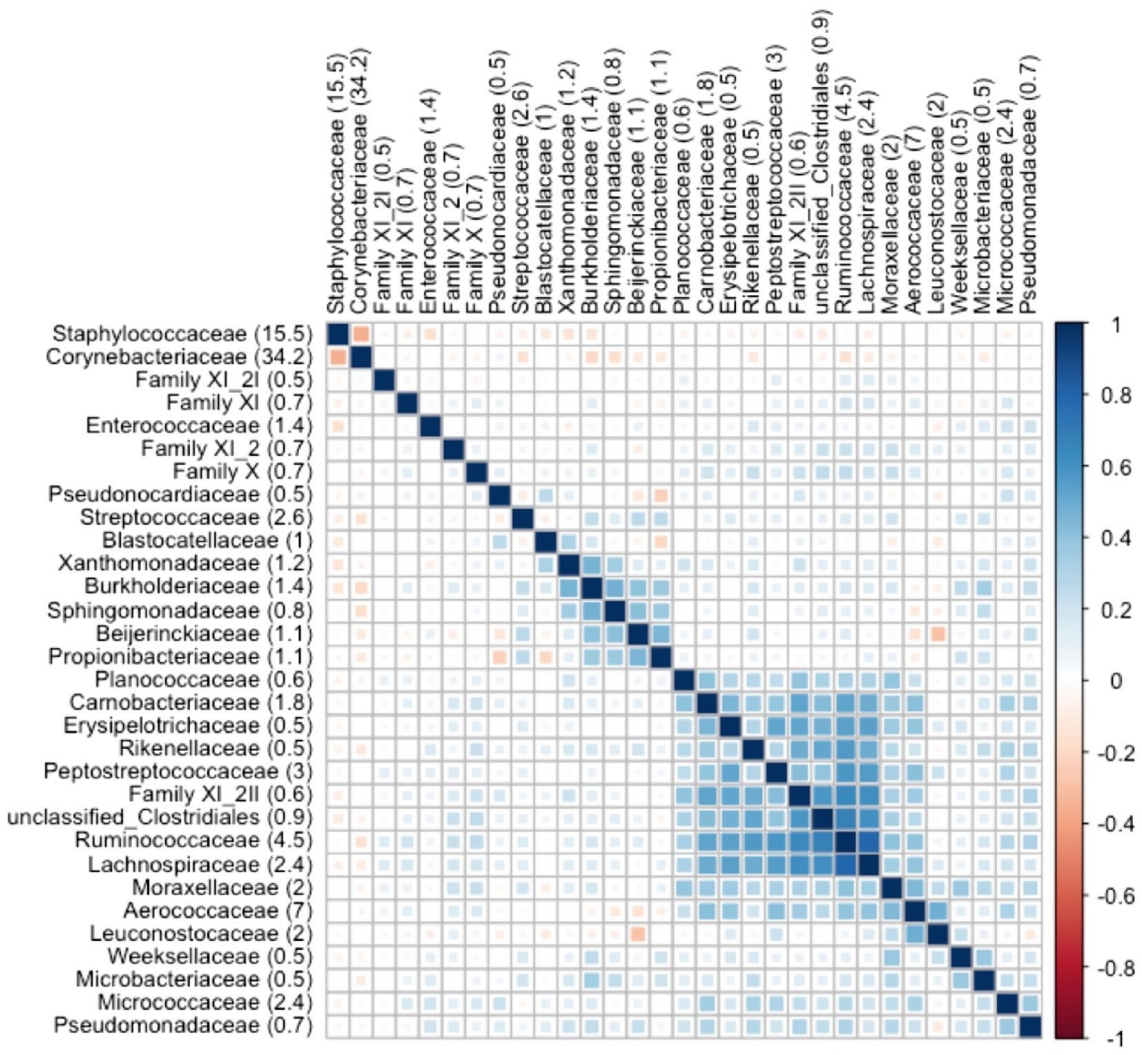
Correlation analysis between the most abundant families found in the all the milk samples. Values after taxa names are the percentage of abundance detected over the entire experiment.

Correlation analysis of the most abundant families were performed to highlight how the presence and abundance of the most abundant families were related to each other. The strongest negative correlation was found between the *Corynebacteriaceae* and *Staphylococcaceae* families (Fig. 3)*.* Positive correlation was found between the families of *Lachnospiraceae, Ruminococcaceae,* unclassified *Clostriales,* Family XI_2 and *Peptostreptococcaceae.* All these families are part of the *Clostridiales* order and accounted for 11.4% of the total reads in the study.

### Individual cow microbiota

**Figure 4:**
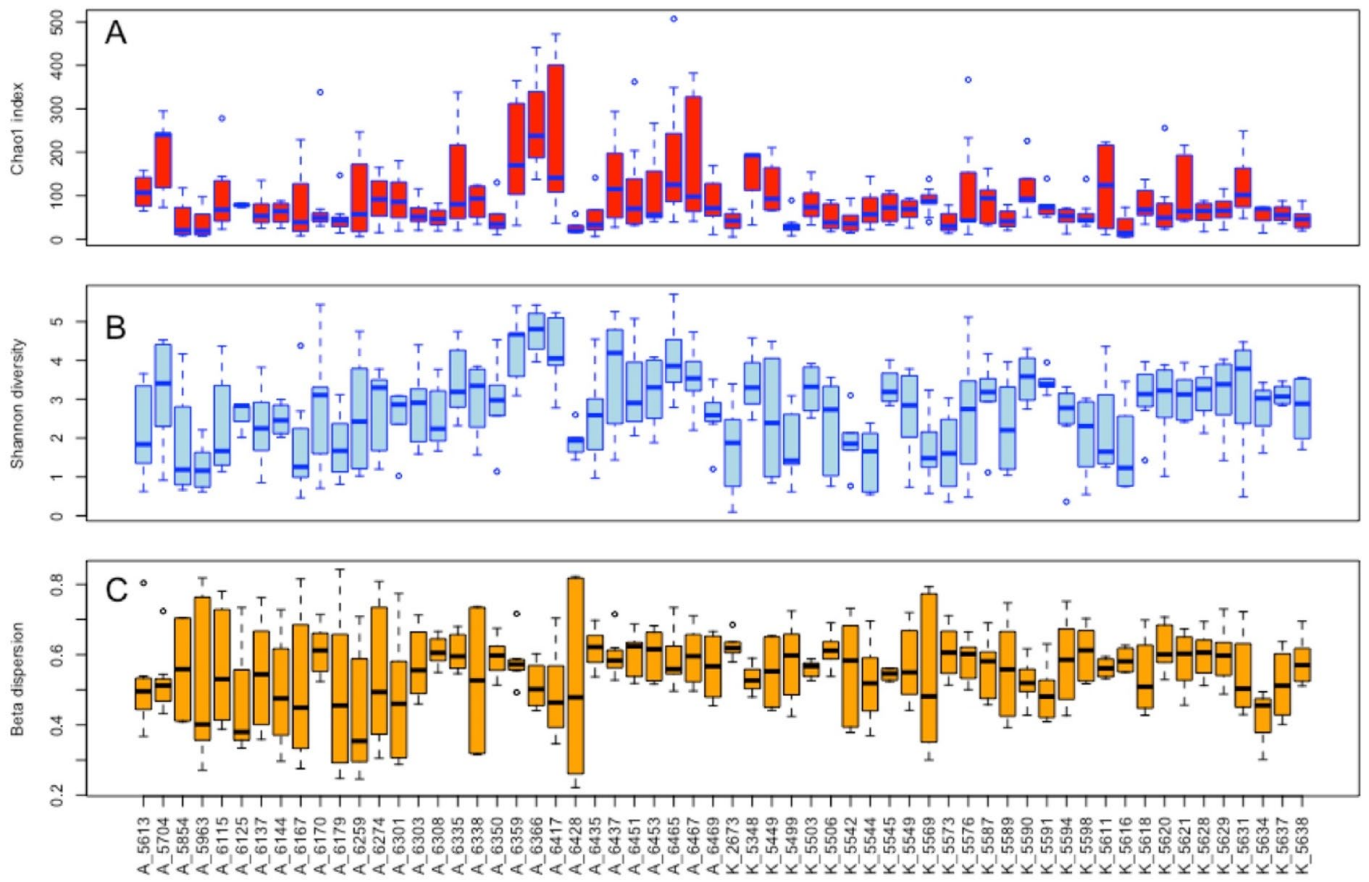
Boxplot of the alpha diversity indexes (**A**: chao1 estimate, **B**: Shannon diversity) grouped per individual cows. (**C**) Multivariate homogeneity of group dispersions obtained using the Bray–Curtis dissimilarity matrix and grouped per cow.

At single cow level, both the alpha diversity indexes (Chao1 estimate and Shannon diversity) were significantly different (*p* < 0.001) indicating that each cow contained a variable bacterial richness and diversity. Beta dispersion analysis of the microbiota within each individual cow indicated that phylogenetic and taxonomic assessments of the reads also revealed a diverse microbial population between quarters from the same cow (Fig. 4). However, the distribution of beta dispersion was larger between cows than within cows (Figure S1).

**Figure 5:**
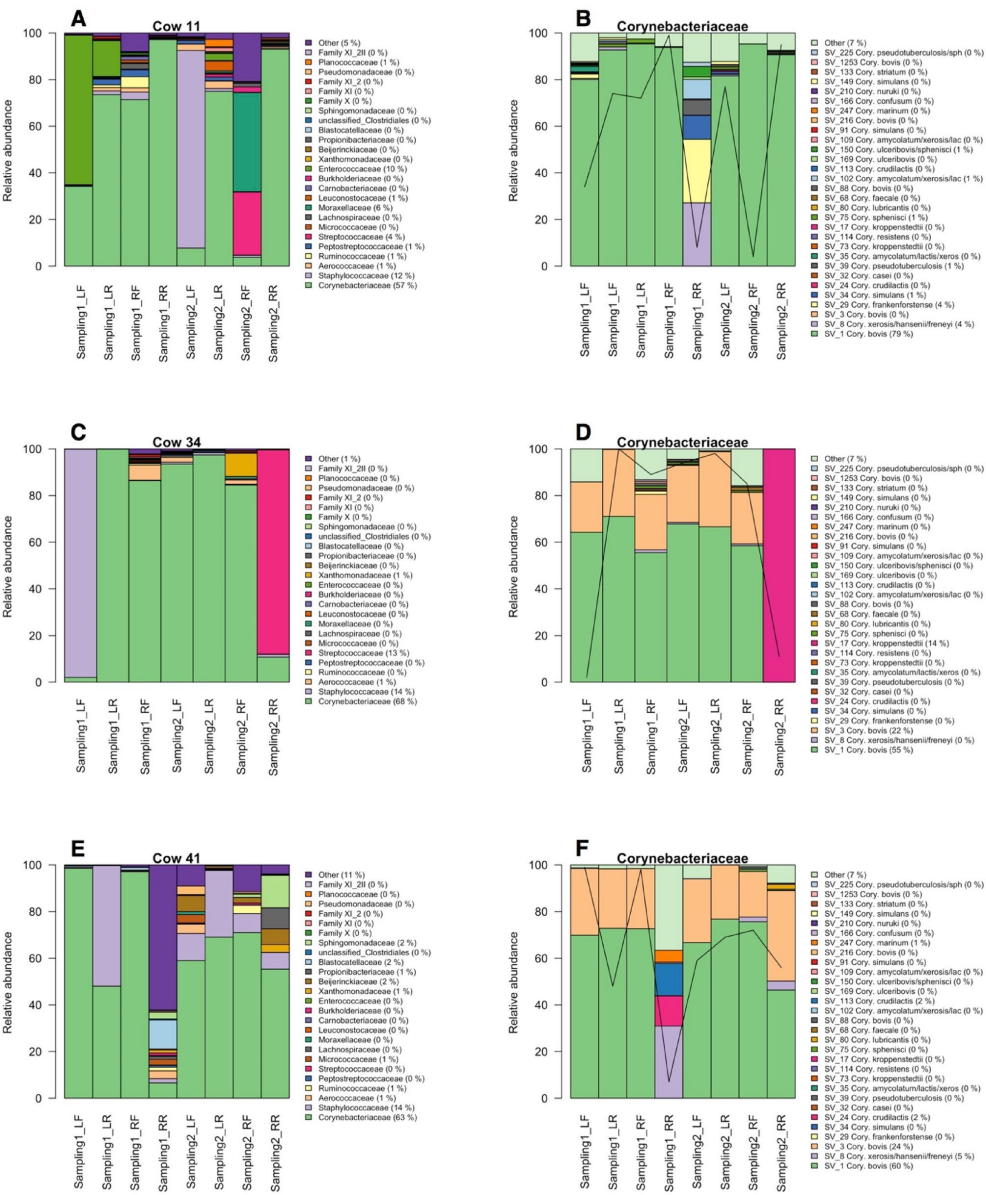
Relative abundance of the quarter microbiota of cow sampled in 2 different occasions (**A**, **C** and **E**) and relative abundance of the sequence variants assigned to the family Corynebacteriaceae for the same cows (**B**, **D** and **F**). Values after the family and Corynebacteriaceae taxa indicate the percentage abundance over the entire value of the plot. Black lines indicate the relative abundance of the Corynebacteriaceae over the total microbiota.

Relative abundance distribution of the quarter microbiota showed that there was a high microbial diversity between cows, between quarters of the same cow and between sampling times of the milk from the same cow (Figure S2). In the case of several cows, one or more quarters showed a microbiota markedly different from the other quarters and was defined here as dysbiotic. Here, we introduced a new concept of a dysbiotic quarter. We used a clear cut off from the relative abundance results of the microbiota and defined a quarter as dysbiotic when one of the taxa, at family level, increases to over 60% of the reads in one quarter milk sample (within each individual and in each sampling) while remaining less than 20% in all the other quarters (this was calculated only on cows where 3 or 4 quarter milk samples were available). In this study, we identified 36 dysbiotic quarters out of 106 analysis (where 3 or 4 quarter milk microbiota was available) and detected that *Staphylococcaceae* (Table S4) was the most common family (20) followed by *Streptococcaceae* (5) and *Enterococcaceae* (4). We also detected good correlation between the enriched family in the dysbiotic quarter and the species identified in the same quarter by culturing method and maldi-TOF analysis. In most of these cases of a dysbiotic quarter, the SV composition of the *Corynebacteriaceae* was also changed (examples in Fig. 5, Figure S2). This was, however, not the case on occasions where a dysbiotic milk sample was detected (examples in Fig. 5 C and D). The shift in *Corynebacteriaceae* composition was detected in at least 15% of the cows (Figure S2). Dysbiotic quarters presented an increased level of potential mastitis-pathogens such as *Streptococcaceae, Aerococcaceae, Enterococcaceae* and other families which were present in high abundance in the dysbiotic quarter but not in the other quarters (Figure S2). Examples of these dysbiotic microbiota are presented in Fig. 5. Two of the quarters of cow 34 (Fig. 5C,D) presented an increased abundance of *Staphylococcaceae* in quarter left front (LF) from the first sampling while the quarter right rear (RR) from sampling 2 was dominated by *Streptococcaceae.* The *Corynebacteriaceae* population was dominated by 2 SVs (assigned to *C. bovis*) in all the quarters except for the quarter containing *Streptococcaceae.* In this quarter these 2 SVs were not detected and the most abundant *Corynebacteriaceae* was assigned to *C. kroppenstedtii.* A further example of dysbiotic udder microbiota with impact on the *Corynebacterium* population was cow 1, where the presence of *Staphylococcaceae* in the quarter RR in sampling 1 was associated with a completely different *Corynebacteriaceae* population within the same quarter. The same pattern was found in cow 41 where the *Corynebacteriaceae* population was different in the dysbiotic quarter, concurrent with increased abundance of *Saccharopolyspora* sp. in quarter RR from the first sampling period.

### Culturing and identification of isolates

Each quarter milk sample was further analyzed by culturing methods using 3 different agar plates (TSA blood, M17 and MRS). The TSA blood agar plate was incubated both in anaerobic and anaerobic condition. Over 2000 isolates were collected and of these only 1150 were classified at species level by maldi-TOF (score > 2). We obtained the majority of the isolates from the TSA blood agar (513 in aerobic condition and 366 in anaerobic condition), while 219 and 52 were obtained from M17 and MRS, respectively (Table S2). Seventy-five different species were detected. The most frequently isolated species was *Aerococcus viridans* (25%), however at genus level *Staphylococcus* was the most abundant genus (45%) and 15 species were identified. *Staphylococcus epidermidis* (22% of the total isolates)*, Staph. chromogenes* (11%) and *Staph. haemolyticus* (4%) were the most abundant *Staphylococcus* identified. Nine species of *Corynebacterium* were identified, and these accounted for 5.5% of the total number of isolates. *Corynebacterium amycolatum* and *C. bovis,* were the 2 most abundant species within this genus. The majority of the *Corynebacterium* isolates (95%) were obtained from TSA blood agar under aerobic conditions.

In addition to isolation of bacteria, growth on TSA blood agar was also used to estimate the number of bacteria present and each quarter milk sample was then divided in different groups based on the number of colonies. We used the definition “A” from Dohoo et al.^14^ as valid for our study and identified quarters with possible intramammary infection if the number of colonies was > 1 per 0.01 mL (> 10 per 0.1 mL in our case). The total number of infected quarters was 173 (42%). By grouping the quarter milk samples in infected and not infected, the microbiota composition was not significantly different (adonis *p* value > 0.05), indicating poor correlation between the two types of analysis. However, the results from sequencing were confirmed by maldi-TOF species identification as the presence of species detected by maldi-TOF can be found in the microbiota at quarter level (Figure S2, Table S4). For example, in cow 11, the presence of several species of *Enterococcus*, *Staphylococcus* and *Streptococcus* was detected by maldi-TOF and a corresponding increase in the relative abundance of the family *Enterococcaceae, Staphylococcaceae* and *Streptococcaceae* was detected in some of the quarter milk samples (Table 2). In cow 34, the presence of *Strep. uberis* and *Staph. epidermidis* is highly correlated with the increase in *Staphylococcaceae* and *Streptococcaceae* in the microbiota of 2 of the quarter milk samples. For cow 41, only 2 quarter milk samples were infected and the maldi-TOF results identified the isolates as *Staphylococcus epidermidis* (in the same quarters). However, the quarter milk samples with dysbiotic microbiota (sampling 1 RR), showed increased abundance in a taxon included in the “other” (low abundance microbiota). Blast results of the SV with the highest abundance suggested the presence of *Saccharopolyspora rectivirgula* (99.51% identity).

**Table 2:** Quarter milk samples with identified dysbiotic microbiota for 3 selected cows (11, 34, 41) and results from the maldi-TOF identification of the isolates for the same milk samples. The microbiota composition of these selected cows is presented in Fig. 5.

Cow ID	Farm	Sampling period	Quarter	Species identified	Dysbiotic milk quarter	Taxonomical classification of enriched family (> 60%)
Cow 11	K	1	LF	*Corynebacterium amycolatum/Enterococcus gallinarum/Staphylococcus epidermidis*	x	*Enterococcaceae*
			LR	*Aerococcus viridans/Staphylococcus warneri*	x	*Staphylococcaceae*
			RF	*Aerococcus viridans/Staphylococcus epidermidis*		
			RR	*Aerococcus viridans/Bacillus pumilus/Enterococcus malodoratus/Lactobacillus plantarum/Staphylococcus epidermidis/Staphylococcus xylosus*		
		2	LF	*Citrobacter freundii/Enterococcus gallinarum/Staphylococcus equorum*		
			LR			
			RF	*Aerococcus viridans/Aeromonas hydrophila/Enterococcus gallinarum/Lactococcus raffinolactis/Streptococcus parauberis*		
			RR	*Aerococcus viridans/Corynebacterium bovis*		
Cow 34	A	1	LF	*Staphylococcus epidermidis*	x	*Staphylococcaceae*
			LR			
			RF	*Aerococcus viridans*		
			RR	*Aerococcus viridans/Streptococcus uberis*	x	*Streptococcaceae*
		2	LF	*Aerococcus viridans*		
			LR			
			RF	*Aerococcus viridans/Pantoea agglomerans*		
			RR	*Streptococcus uberis*		
Cow 41	A	1	LF			
			LR	*Staphylococcus epidermidis*		
			RF			
			RR			
		2	LF			
			LR	*Staphylococcus epidermidis*		
			RF			
			RR	0	x	*Pseudonocardiaceae*

## Discussion

The growing interest in understanding the complex bovine udder microbiome has resulted in several published studies in recent years. These studies aimed to uncover how this microbiome influences the udder health and possibly has an important role during mastitis. In this study, we contribute to this knowledge by exploring the milk microbiota with a cross-sectional study of the milk microbiota as elucidated from over 400 quarter milk samples obtained from 60 lactating cows.

Sampling of udder milk for microbiome analysis is difficult and challenging^15,16^. In order to accomplish a more representative overview of the milk microbiota that colonize the upper interior part of the udder, the sampling method used in this study was different to those previously used to study milk microbiota^2,6^. The sampling was performed after the cow was regularly milked with the intent to remove microbial contaminations from the teat apex and to avoid the sampling of milk present in the cistern which might contain bacteria able to enter the udder between milking. The complete removal of contaminations from the teat apex or the environment cannot be ensured by the results obtained in this study, and additional experiments are needed to compare microbiota from milk samples obtained pre- and post-milking. However, although some taxa associated with the environment, such as *Bacillaceae* and *Pseudomonadaceae,* were detected in the dataset their abundance was much lower (< 1%) than that found in milk collected from bulk farm tanks which included organisms present in cows as well as milk equipment and the farm environment^17–19^. Interestingly, by using this sampling regime, we obtained a microbiota that was highly dominated by two families (*Corynebacteriaceae* and *Staphylococcaceae*). Several other taxa commonly associated with mastitis and as part of the milk microbiota were also detected. These included *Aerococcaceae*, families within the order *Clostridiales, Streptococcaceae* and *Enterococcaceae.* The sampling regime used in the study resulted in a similar or lower bacterial richness in our samples compared to previous studies^20–22^. However, comparisons with other studies cannot be conclusive due to different sampling procedures, filters and bioinformatics pipelines used between the studies. In particular, the method used for SV inferences increases the richness detected of microbiota studies compared to open-reference OTU clustering methods^23^.

The most common taxa found in the quarter milk samples are frequently reported in previous milk microbiota studies^2,20^. The family *Corynebacteriaceae*, which includes only the genus *Corynebacterium,* has been previously described as part of the skin-associated microbiome in humans and it is also found in other bovine body sites, such as the teat canal and uterus^24–27^. Therefore, it was not unexpected to find a high abundance and frequency of *Corynebacteriaceae* in quarter milk samples. This was similar to previous reports of milk microbiota^2,6^. Surprisingly, this taxon was found in almost all the quarter milk samples from two different farms and was detected in all the 60 cows. This suggests that *Corynebacterium* is part of the udder core microbiota revealed in this study. The role of *Corynebacterium* within the udder is still unknown and this might include a protective role against mastitis pathogens. Competition for niche adaptation between *Corynebacterium* and other taxa and inhibition of pathogen bacteria has been previously reported in microbiome studies^28,29^. Of particular interest was the interaction between *Corynebacterium* and *Staphylococcus.* Here, we found that the two main taxa of this study were negatively correlated with regards to their abundance. It is not possible to know if this negative correlation arose from the increase in abundance of one of the two taxa in the quarter or the decrease of one of them because of the lack of information of the absolute number of bacteria. However, competition between *Corynebacterium* and *Staphylococcus* has been shown previously in different environments and the negative correlation found in this study is further confirmation^28,30,31^. Another indication of a possible protective role of *Corynebacterium* against dysbiosis can be drawn from the analysis of the composition of the *Corynebacterium* population itself. The *Corynebacterium* population was different in dysbiotic quarters and this was shown in several cows. In most of these cases, this dysbiosis was caused by the presence of mastitis pathogens, such as *Streptococcaceae* and *Enterococcaceae,* which might outcompete and replace the dominant *Corynebacterium* SVs found in the other quarters of the same cow.

Several species of *Corynebacterium* inhabit the udder microbiome and Hoque et al.^11^ identified 12 of them using a metagenomics approach. In this study, the most common *Corynebacterium* SVs were assigned to *Corynebacterium bovis,* but several other species were detected by amplicon sequencing and MALDI-TOF identification of isolates. *Corynebacterium bovis* was previously described as part of the teat canal microbiota and Hiitio et al.^12^ also detected this species in the upper part of the gland*. Corynebacterium bovis* is classified as a minor mastitis pathogen and usually targeted during routine testing by mastitis laboratories. In our study, we found that *C. bovis* was present in a large number of quarter milk samples and several isolates were also obtained by culturing. By using standard culturing methods, Goncalves et al.^32^ detected *C. bovis* in only 15.8% of composite milk samples and using sequencing methods Taponen et al.^2^ detected that *C. bovis* covered between 50 and 73% of all *Corynebacterium* reads in milk samples that were PCR positive for *C. bovis*. The presence of *C. bovis* in milk has also been linked to an increase number of somatic cells in the cow’s milk and to the changed milk composition in subclinical mastitis^32^.

The genus *Staphylococcus* contains one of the most frequent and well-known mastitis pathogens, *Staph. aureus.* The samples analyzed in this study were obtained from cows with no signs of clinical mastitis and this pathogen was infrequently found in our samples and was not the most abundant *Staphylococcus* species isolated. On the other hand, the non-aureus staphylococci (NAS), which included several species of skin-related and the minor mastitis pathogens, were the most abundant group within the *Staphylococcaceae* family. Similar to *Corynebacterium,* NAS are commonly found in milk microbiota and in other microbiomes such as the bovine teat canal and skin^12,33^.

High correlations between the incidence of several families of the order *Clostridiales* were detected. These families accounted for more than 10% of the total microbiota in this study and are known to inhabit the cow rumen^34,35^. Their high positive correlation might indicate that their presence in the milk microbiota was connected. Rumen-related families within the Clostridiales order are often detected in milk microbiota studies. Vasquez et al.^5^ found that members of the *Clostridiales* such as *Ruminococcaceae* and *Lachnospiraceae* were among the dominant taxa in milk microbiota. However, the presence of these families in milk might originate from contamination during sampling procedure from the farm environment, the teat canal or from the translocation of cow microbiota through the hypothesized endogenous entero-mammary pathway^3,36^. Another aspect might be that families of the order *Clostridiales* are able to colonize the interior of the udder due to favorable anaerobic conditions and presence of nutrients. None of the over 1000 isolates obtained were classified within the order *Clostridiales*. This could be due to their fastidious growth requirements as this order includes obligative anaerobe spore-forming genera.

In this study, we collected milk samples from each quarter and a considerable difference in microbiota was found for samples collected from the same cow. This was detected in several individuals. Despite factors pertaining to the individual cow, such as immunity and genetics, that are known to influence the udder microbiota^37^, our results indicated that within-individual microbial variation is frequent. This is supported by the fact that certain taxa were often detected in only one quarter, rather than in all four quarters. Examples of these are found in this study where, in particular, the presence of *Streptococcaceae* and *Enterococcaceae* was detected in some quarters of the same cow but not in all four.

The milk microbiota structure was found to be influenced by farm and sampling period and several of the most abundant taxa were found to be influenced by these factors. The reasons for this variation in composition of quarter microbiota between the two farms studied may be mainly due to different farming practices, animal variability and genetics. Farming practices, such as bedding material, milking systems, housing system have previously shown to have an impact on the milk microbiota^38,39^. Whether or not the farming practices used in both farms in this study (e. g. cows regularly milked in an automatic milking system, open barn environment) influenced the microbiota inside the udder cannot be fully confirmed. However, since hygienic conditions have been found to be important for the transmission of mastitis pathogens within the herd and it can therefore be hypothesized that taxa able to colonize the udder can be spread within the herds, thus increasing the differences in diversity between farms^40^.

The variation in microbiota between sampling periods can be attributed both to the season when the sampling occurred and to the days of lactation of the animals. Interestingly, *Streptococcaceae* were significantly more abundant in the second sampling which was performed between late Aug-Sep. Species belonging to the *Streptococcaceae* family, such as *Strep. dysgalactiae, Strep. uberis* and *Strep. agalactiae* are common mastitis pathogens. Previous studies showed that cows have a greater risk of *Streptococcus* mastitis during the summer months compared to the winter months^41,42^. Therefore, it is not unexpected that this taxon was more abundant in the second sampling period compared to the first one. The presence of *Streptococcus* in milk samples was confirmed by the identification of the isolates.

The term dysbiosis can be defined as an alteration of the normal microbiota due to loss of diversity, reduction of commensal microbes and usually presence of pathogens which outcompete the existing microbiota. Several studies showed that disruption of diversity in udder microbiota is linked to a change in the health status of the udder and, in case of mastitis, the microbiota is dominated by the pathogen^11,12^. An important challenge for defining dysbiosis is the definition of a “normal” or “baseline” microbiota. Here, we decided to compare the microbial composition within the same individual and sampling to identify the “normal” microbiota and the presence of dysbiosis in only one quarter. By using this approach, we were therefore not able to identify dysbiosis that might occur in more than one quarter. However, we identified a high prevalence of dysbiosis in the bovine udder and a good correlation between the detected dysbiosis and isolation of bacterial species (within the same taxonomy) identified by maldi-TOF.

While the data presented here further support that a microbiota is present in the udder and might play a protective role, a few limitations of this study need to be considered. The somatic cell counts (SCC), used as indicator for the udder health, were not recorded at the time of sampling and indications of the health status of the quarters was obtained with culturing methods, which are biased by the conditions used for culturing and might therefore not reflect the true status. However, samples were collected from lactating cows, milked during regular farm practices with no sign of clinical mastitis during the collection days. Most microbiota studies suffer from the lack of absolute number of bacteria and results are presented in relative abundance where each feature is constrained to each other. Changes in abundance of one taxon will therefore impact the relative abundance of the others. The microbiota study presented here was performed using short-read sequencing, a method which achieves a lower resolution during taxonomical assignation of the reads. Furthermore, milk from cows with no sign of clinical mastitis harvest a low abundance microbial biomass and in case of an introduction of one taxon into the udder, this might have a big impact on the abundance of the resident microbiota of the udder. The cow breed has previously shown to be an important factor shaping the milk microbiota^22^. In this study, we selected 60 cows of Norwegian Red breed and therefore our results need to take into consideration possible breed-to-breed diversity when compared to other studies where more popular breeds are used (e. g. Holstein or Friesian). The microbiota of milk from the udder of Norwegian Red cows has not previously been reported.

## Conclusion

The understanding of the complexity of the udder microbiome is enriched with the increased number of studies and some additional conclusions can be drawn from this work. The milk collected from mammary quarters contained a complex microbiota which was composed primarily by the two taxa, *Corynebacteriaceae* and *Staphylococcaceae.* These two taxa were detected in all the cows and are thus identified as part of the core microbiota with potential implications for the health of the udder. Evidence of interactions between *Corynebacteriaceae* and *Staphylococcaceae* for niche adaptation are also shown by the negative correlations of their abundances. Disruption of “normal” microbiota (dysbiosis) was detected frequently in quarters showing, in most of the cases, the presence of mastitis-related pathogens. This occurred in cows used for regular milk production and our results provide evidences that the microbiome balance within the udder is dynamic and very fragile and subject to several shaping factors both from the host and from the environment. This study is an additional step to decipher the potential role of the udder microbiome in cow health.

## Materials and methods

### Study animals

Sixty Norwegian Red cows selected from two dairy herds in the south-eastern part of Norway were included in this study. Thirty cows were sampled from the “Centre for livestock production” at the Norwegian University of Life Sciences (farm A) and thirty cows were selected from the “Kalnes Upper Secondary School” farm (farm K). Both farms operate under the regulations of the Norwegian Food Safety Authority regarding food production and animal care. The farm owners provided their permission for the sampling and for the use of their information in this study. Information about farm management is presented in Table S1. Samples were collected without the use of invasive procedures. Metadata from each individual cow were obtained from the Norwegian Cattle Health Recording System^43^. Cows were considered healthy and included in the study if (1) no sign of clinical mastitis were detected by the personnel, (2) there was no record of mastitis (reported by the farmers or the veterinarians) in the Norwegian Cattle Health Recording System 2 weeks before the sampling and (3) the cows were not under any antimicrobial treatment.

### Sample collection

Milk samples were collected from each quarter in two occasions (January–March 2018 and late August–September 2018) corresponding to the early and late lactation period. At each day of sampling, 5 cows were selected thus obtaining 20 quarter milk samples which were used for analysis. Only 2 samplings per week were performed. On the day of sampling, collection of the milk was performed towards the end of the regular milking routine by trained personnel. After removal of the milking apparatus, milk (50 mL) was collected following the “Procedure for Collecting Milk Samples” of the National Mastitis Council (NMC, www.nmconline.org). The sample tubes were immediately stored in ice and transported to the laboratory within 2 h from the last sampling and immediately prepared for analysis. A total of 430 samples were successfully collected during the experiment. Samples were not included in the analysis if the cow showed signs of mastitis or the cow was no longer part of the herd during the second sampling.

### Microbiological analysis and DNA extraction

Milk samples for microbiota analysis (40 mL) were centrifuged in an Heraeus Multifuge X3R centrifuge (ThermoFischer Scientific, Massachusetts, United States), using a FIS-6X100Y Fiberlite Rotor (ThermoFischer Scientific) at 8000 rpm for 10 min. Sterile foam coated Critical Swab cotton swabs (VWR, Pennsylvania, United States) were used to remove the cream layer. The supernatant was discarded, and the milk pellet resuspended and collected in 1.5 mL Eppendorf tubes that were immediately frozen at − 20 °C awaiting further processing. DNA extraction was performed using the DNeasy PowerFood Microbial Kit (Qiagen, Düsseldorf, Germany) starting from step 2 in the detailed protocol DNeasy Powerfood Microbial Kit Handbook. For increased efficiency in lysis of difficult species an additional incubation for 10 min at 65 °C on a heat block was performed at step 4 in the protocol, before proceeding with the rest of the protocol from step 5. Elution of DNA was performed in 50 µl of elution buffer before samples were placed in storage at − 20 °C.

For growth and identification of isolates, 100 µl raw milk were plated on de Man, Rogosa, and Sharpe agar (MRS; Difco Laboratories, Detroit, MI), M17 agar (Merck, Darmstadt, Germany) and TSA blood agar plates (ThermoFischer Scientific, Massachusetts, United States). Plates with MRS and M17 were incubated at 30 °C under anaerobic conditions for two days, while blood plates were incubated at 37 °C under both aerobic and anaerobic conditions for 24 h. Airtight containers and AnaeroGen 3.5L sachets (ThermoFischer Scientific) were used to achieve anaerobic conditions. After incubation, up to 10 phenotypically distinct bacterial colonies (representing the most phenotypic diversity on plate and not the proportion of the total isolates on the plate) were streaked out on new plates and re-incubated. All strains were collected and stored at -80 °C in cryomedia with 5% DMSO and 15% glycerol. TSA blood agar plate were also used to group the milk quarter milk samples based on the number of colonies present. The plates were grouped in 0 colonies, 1 < 10, 10 < 100, 100 < 1000 and > 1000 colonies. We considered the definition “A” from Dohoo et al.^14^ as valid for our study and identified quarters with intramammary infection if the number of colonies was > 1 per 0.01 mL (> 10 per 0.1 mL in our case) in one of the two conditions used for TSA blood agar (aerobic, anaerobic).

### Library preparation and sequencing

DNA concentration was measured for all samples using the NanoDrop ND-1000 spectrophotometer (NanoDrop Technologies, Inc, Wilmington, DE, USA). The V3 and V4 region of the 16S rRNA was amplified using the primers Uni340F (CCTACGGGRBGCASCAG) and Bac806R (GGACTACYVGGGTATCTAAT). The PCR reaction contained 1× of the Q5 Hot Start High-Fidelity 2× Master Mix (New England Biolabs, Massachusetts, United States), 1× of EvaGreen Dye 20× in water (Biotium, California, United States), 0.5 µM of each primers and three microliter of DNA in a final volume of 20 µL. Amplification was performed in a LightCycler 480 Instrument II (Roche, Basel, Switzerland) using initial denaturation at 98 °C for 30 s, followed by 35 cycles of denaturation at 98 °C for 15 s, annealing at 53 °C for 30 s and elongation at 72 °C for 20 s. The final elongation was performed at 72 °C for 10 min. The PCR product was then purified using 0.7× of Agencourt AMPure XP beads (Beckman Coulter, Inc, Brea, CA, USA) according to the manufacturer's instruction. A second PCR was performed using the purified PCR product (4 µL) to incorporate the primers with adapters and the P5 and P7 Nextera indexes (Illumina, San Diego, CA, USA). The PCR conditions were the same as above except that annealing temperature was 55 °C and a total of 10 cycles were used. Libraries were cleaned and normalized using the SequalPrep Normalization Plate (96) Kit (ThermoFischer Scientific) and pooled together. The final library concentration was then measured using Qubit 2 with the dsDNA HS kit (ThermoFischer Scientific) and quantitated using the KAPA Library Quantification kit (Illumina) before being sequenced on an Illumina MiSeq platform (Illumina) using the 2× 300 bp V3 kit (Illumina). Sequence data that support the findings of this study have been deposited in the EBI data with accession number: PRJEB35792.

### Sequence data analysis

Sequence data quality were evaluated using FastQC version 0.11.5 (https://www.bioinformatics.babraham.ac.uk/projects/fastqc). Reads were quality filtered and trimmed using the Dada2 package^44^ using truncating of forward reads set to 265 bases and truncating of reverse reads set to 220 bases. The error model in Dada2 was created using 1 million random filtered reads. Sequence variants (SV) was inferred using the DADA2 algorithm^44^ and removal of chimeras was performed using the function “removeBimeraDenovo” in the Dada2 R package. Sequence variants shorter than 375 base pairs were removed from the final table. Taxonomy was assigned using the Decipher R package^45^ against the SILVA SSU database^46^. Samples with less than 2000 reads and SV with less than 10 sequences were removed from the table. Normalization of the SV table was performed using the metagenomeSeq R package using the cumNorm function^47^. Individual SV sequences were also queried against the BLAST non-redundant (nr) database for taxonomical identification of the species or group of species. The sequences with the highest % identity and lowest e-value where used to assign the name of the species (or group of species if similar results were obtained) to the SV. This was done for SV assigned to the genus *Corynebacterium* and *Staphylococcus* (Table S3)*.* The most abundant sequence variants assigned to the genus *Staphylococcus* were classified as non-aureus staphylococcus (NAS) if they obtained the highest identity with one of the main NAS species identified in mastitis cases (*Staph. chromogenes, Staph. simulans, Staph. epidermidis, Staph. haemolyticus* and *Staph. xylosus*). Negative and positive controls were included for each sequencing run. Negative controls were included during (1) sampling by using a sterile 50 mL falcon tubes with 20 mL ultraclean PCR grade water Sigma W45002 (Merck, Germany) (2) DNA extraction using only extraction kit reagents and plastic were also included (3) library preparation using PCR grade water. The positive controls were used during library preparation and for each round of sequencing and consisted of a serial dilution of the ATCC MSA 2002, 20 Strain Even Mix Whole Cell Material (ATCC, Virginia, United States). Positive and negative controls sequence data were used to filter potential contamination from the data. The R package “decontam” was used to remove SV which were identified as contamination during each sequence run. In addition, SVs present in positive controls but not included in the list of bacteria of the ATCC MSA2002 were also removed from the final dataset. In total 4592 SV were removed from the experiment and of these 72.3% were not identified at family level (Table S5) and accounted for 10.6% of the total reads.

### Statistical analysis

All statistical analyses were performed using the R software^48^. Chao1 estimate and Shannon diversity were chosen to evaluate the alpha diversity and were calculated using the R package Vegan^49^. Pairwise comparison of the alpha diversity indexes between group levels was performed using the Wilcoxon rank-sum test. Multivariate homogeneity of group dispersions was calculated using the function “betadisper” available in the R package Vegan. Permutational analysis using dissimilarity matrix (“adonis” function from the R package Vegan) was used to test differences in the composition of the community between groups of samples (n. of permutation 999). Bray–Curtis and Jaccard dissimilarity matrixes were selected as input for ordination analysis using non-metric multidimensional scaling (NMDS) and the results of these two ordinations were compared using Procrustes analysis^50^. Linear discriminant analysis effect size^51^ was used to identify families which were significantly more abundant among groups of samples. Correlation analysis between the 30 most abundant families was performed using Pearson correlation coefficient for all individual taxa based on their relative abundance.

### Isolates identification

The MALDI-TOF Biotyper system (Bruker, Bremen, Germany) was used for identification of the isolates at species level. Fresh cultures were prepared from the frozen stock by inoculation in the same media of isolation. A single colony was collected and applied to ground steel Maldi target plates (Bruker) using sterilized wooden toothpicks and then 1 µL 70% formic acid was added for on-plate direct extraction before applying 1 µL HCCA matrix (Bruker) and identification. Automated data analysis of the raw spectra was performed using the MALDI BioTyper 1.1 software (Bruker) with default settings to create a list of the most significant peaks of the spectrums. Each spectrum was automatically compared with the MALDI Biotyper database using the parameter of the pattern-matching algorithms. Identification of the bacterial species was considered only for results > 2.

## Supplementary information

Supplementary Captions.

Supplementary Figure 1.

Supplementary Figure 2.

Supplementary Table 1.

Supplementary Table 2.

Supplementary Table 3.

Supplementary Table 4.

Supplementary Table 5.
