# Supplementary material for: A core microbiota dominates a rich microbial diversity in the bovine udder and may indicate presence of dysbiosis
Source: Sci Rep. 2020 Dec 10;10:21608. doi: 10.1038/s41598-020-77054-6 (PMC7729973; doi:10.1038/s41598-020-77054-6)
Supplement: Supplementary file 1 — Supplementary Captions. [file 41598_2020_77054_MOESM1_ESM.docx]

**TITLE: A core microbiota dominates a rich microbial diversity in the bovine udder and may indicate presence of dysbiosis**

**Authors: Davide Porcellato** ^*1^**, Roger Meisal** ^1,2^**, Alberto Bombelli** ^1^**, Judith A. Narvhus** ^1^

^1^ Faculty of Chemistry, Biotechnology and Food Science, the Norwegian University of Life Sciences, P.O. Box 5003, N-1432 Ås, Norway.

^2^ Present address: Møreforsking Ålesund AS, Ålesund, Norway

***corresponding Author:**

Davide Porcellato, Postbox 5003, 1432 Ås, Norway

Tel.: +4764965143

E-mail address: [davide.porcellato@nmbu.no](mailto:davide.porcellato@nmbu.no)

**List of supplementary materials**

Table S1. Farm management practices, average day in milking and percentage of parity among the cow used in the experiment grouped for sampling and farms.

Table S2. Bovine milk isolates identified at species level using MALDI-TOF-MS.

Table S3. Taxonomical identification of the 30 most abundant sequences assigned to the family *Corynebacteriaceae* and *Staphylococcaceae*. Sequences were blasted against the NR database (NCBI) and the genus and species names were obtained from the best hits with highest identity, and lowest e-value. Only hits with identity greater than 98.5 % were kept.

Table S4. Presence of dysbiotic quarters milk samples grouped per cow (with family taxon) and maldi-TOF identification of the different isolates in the four quarter for each cow. Dysbiotic quarter was identified as one quarter having an increased % of one (or more) taxa over 60% while the same taxa was present in less than 20% in all the other quarters of the same cow during the same sampling period. Only sampling period with more than 3 quarter included in the microbiota results were analyzed. NA indicated that there were less than 3 quarter available for analysis and therefore it was not possible to identify the presence of dysbiosis.

Table S5. Number of read obtained in the whole experiment during bioinformatics analysis and distribution of taxonomical classification of sequence variants removed from the experiment because present in negative controls

Figure S1. Boxplot of the beta dispersion analysis of the microbiota between (centroid values were used) and within each individual cows.

Figure S2. Relative abundance of the bovine quarter microbiota (left), *Corynebacteriaceae* population (center) and somatic cells count (SCC) for each cow during the year of sampling (2018). Values after the family and *Corynebacteriaceae* taxa indicate the percentage abundance of each taxa on the plot. Black lines indicate the relative abundance of the *Corynebacteriaceae* over the total microbiota. Only cows with 6 or more quarters available for analyses were used for plotting. Values after the sample names were the recorded number of isolates (from blood agar aerobic or anaerobic). * indicate the samples classified as infected in this study. Vertical red lines on the SCC plot indicate the date of sampling and horizontal lines indicate the SCC values (in 1000 SCC per mL of milk). The table under the SCC plot is the distribution of the bacterial species isolated and identified by maldi-TOF.
