# Supplementary figures and images for: A core microbiota dominates a rich microbial diversity in the bovine udder and may indicate presence of dysbiosis
Source: Sci Rep. 2020 Dec 10;10:21608. doi: 10.1038/s41598-020-77054-6 (PMC7729973; doi:10.1038/s41598-020-77054-6)

**Distance between cows**

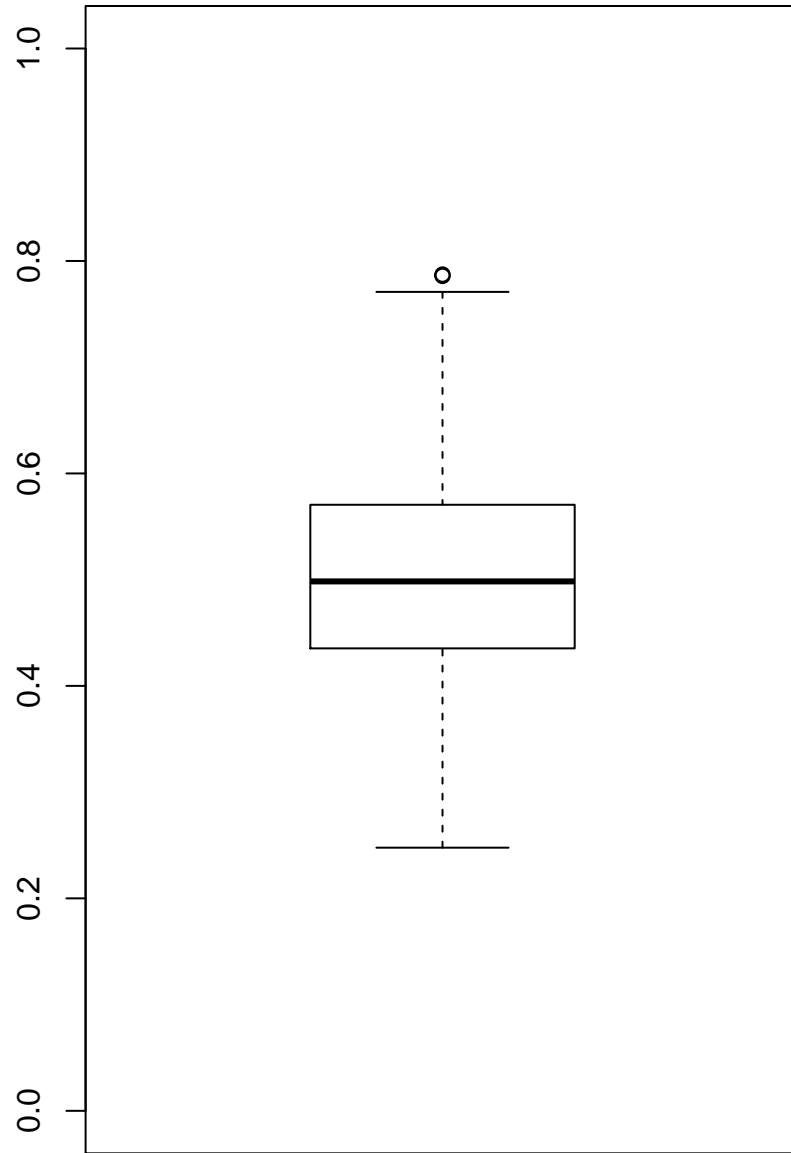

**Distances within cows**

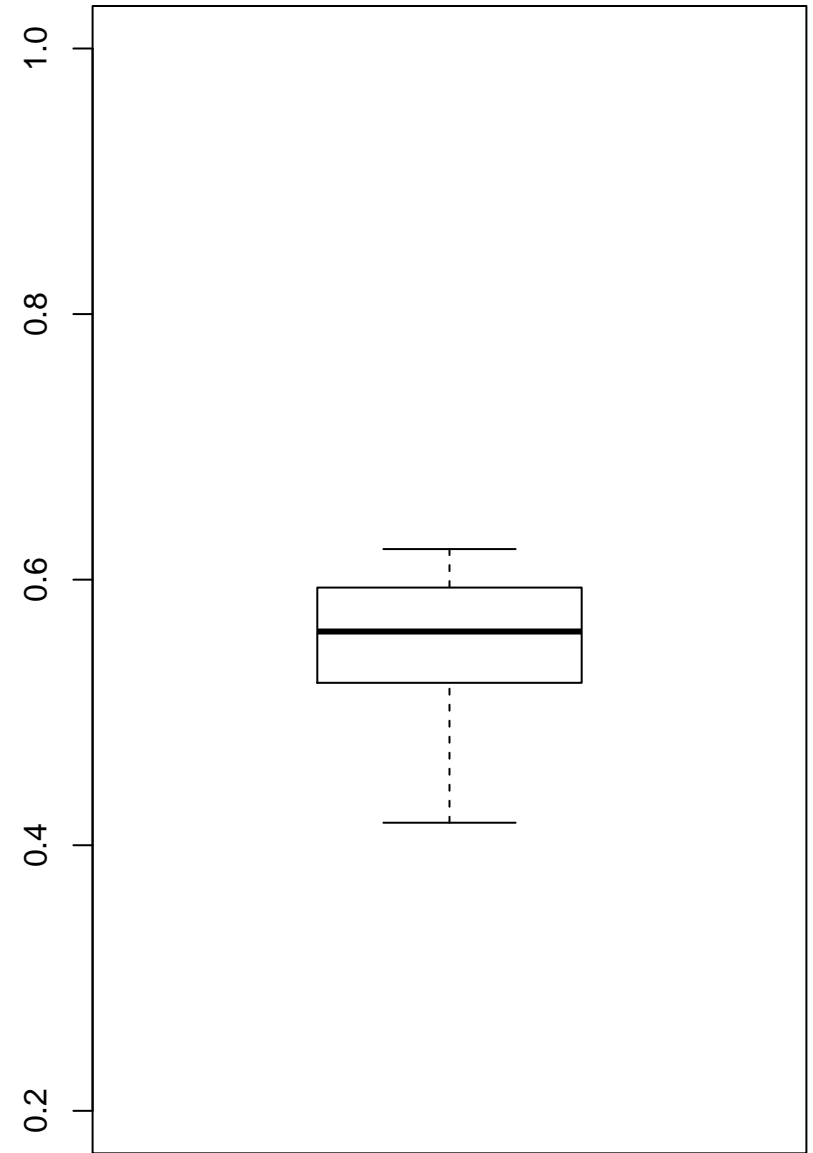

Supplement: Supplementary file 2 — Supplementary Figure 1. [file 41598_2020_77054_MOESM2_ESM.pdf]
