# Supplementary material for: A core microbiota dominates a rich microbial diversity in the bovine udder and may indicate presence of dysbiosis
Source: Sci Rep. 2020 Dec 10;10:21608. doi: 10.1038/s41598-020-77054-6 (PMC7729973; doi:10.1038/s41598-020-77054-6)
Supplement: Supplementary file 3 — Supplementary Figure 2. [file 41598_2020_77054_MOESM3_ESM.pdf]

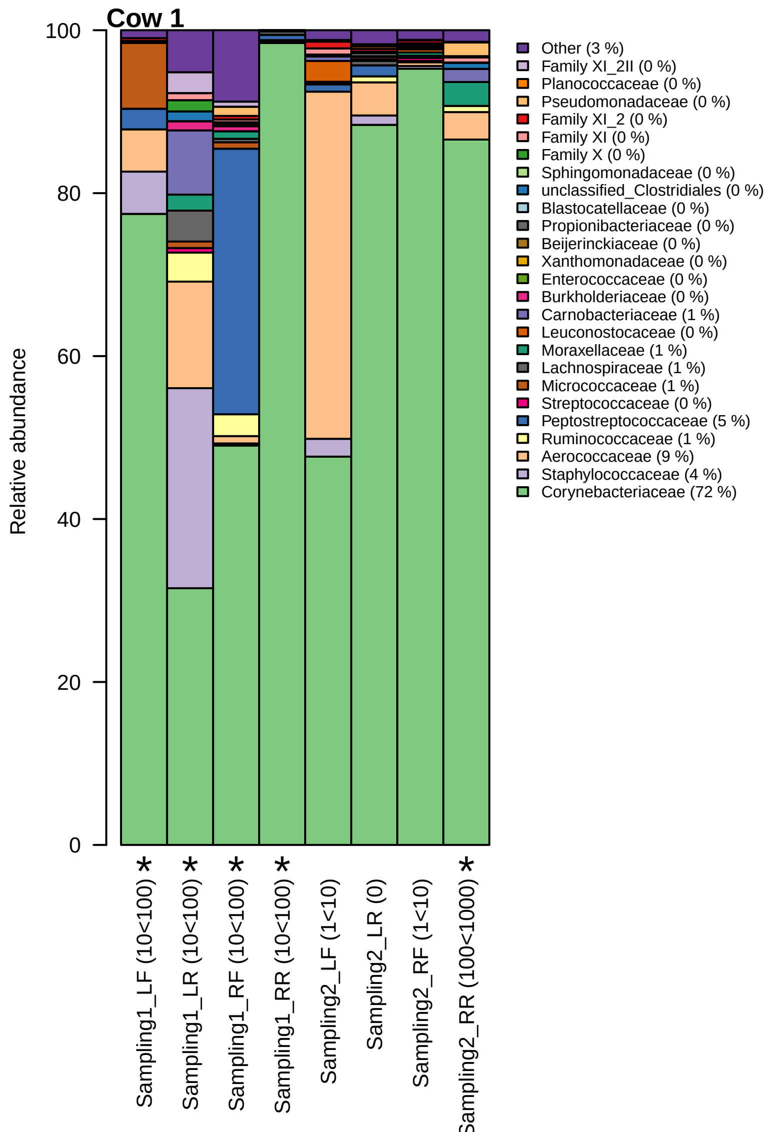

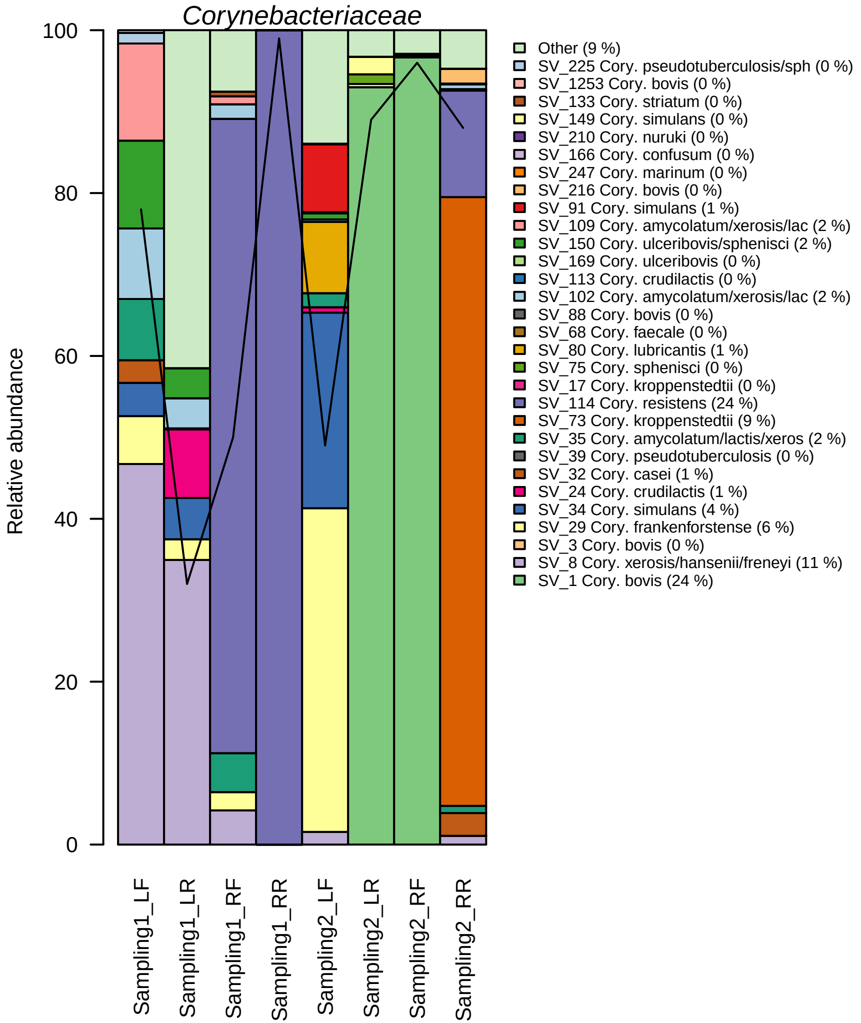

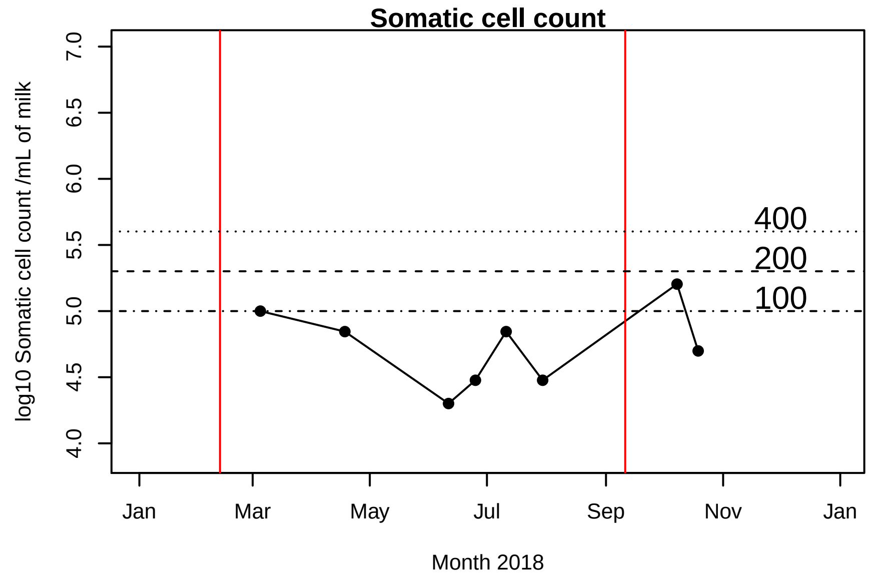

	# quarter	Sampling 1	Sampling 2
**Aerococcus viridans**	4	6	2
**Bacillus licheniformis**	1	1	0
**Bacillus pumilus**	2	2	0
**Corynebacterium amycolatum**	2	2	1
**Corynebacterium sp**	1	1	0
**Corynebacterium xerosis**	1	1	0
**Staphylococcus epidermidis**	4	4	0
**Staphylococcus haemolyticus**	1	1	0
**Staphylococcus hominis**	1	0	1

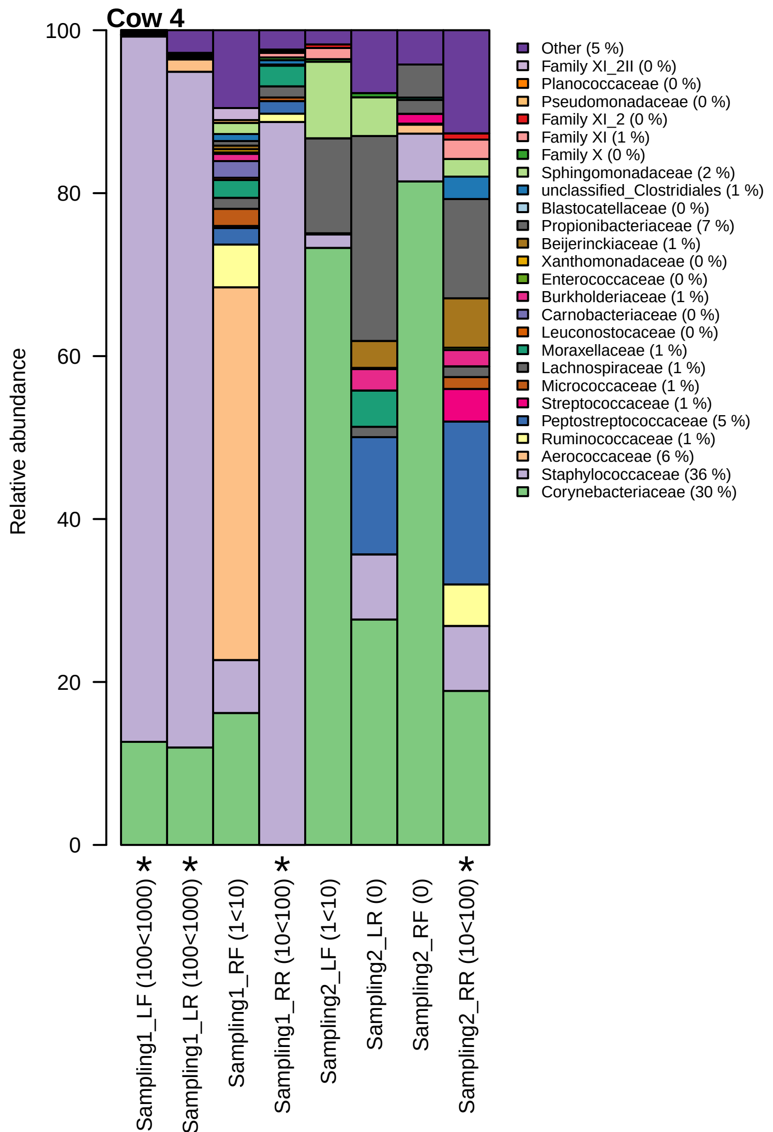

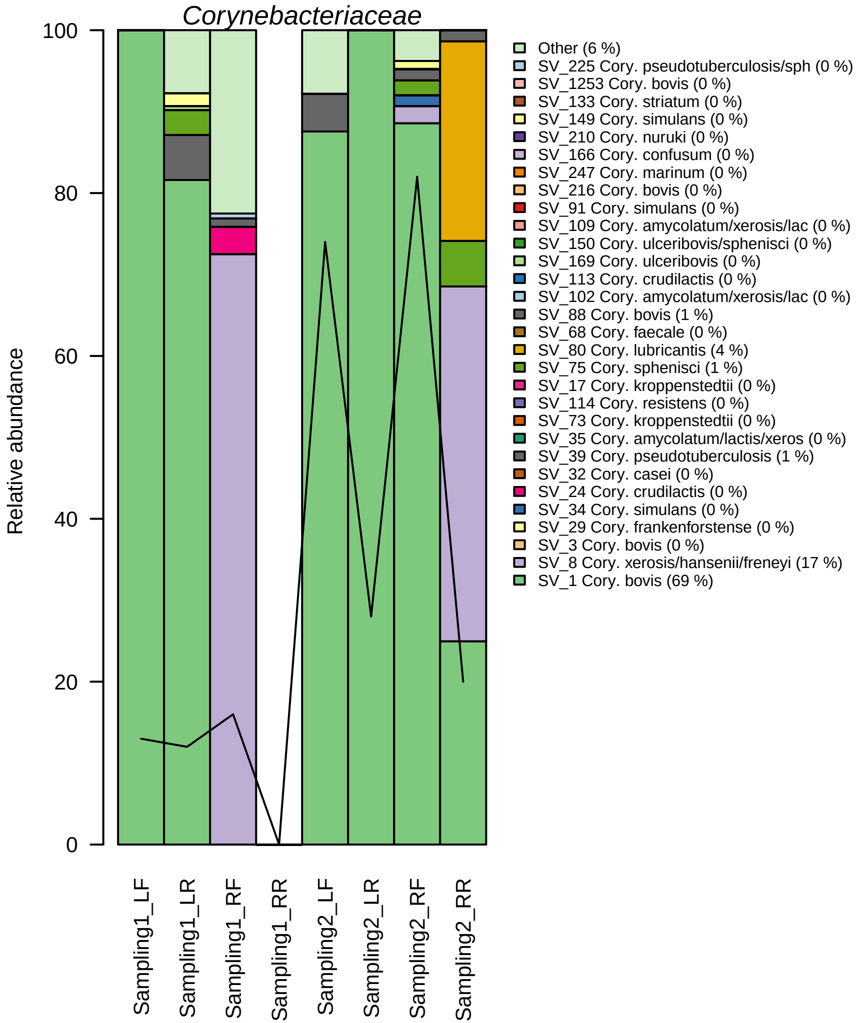

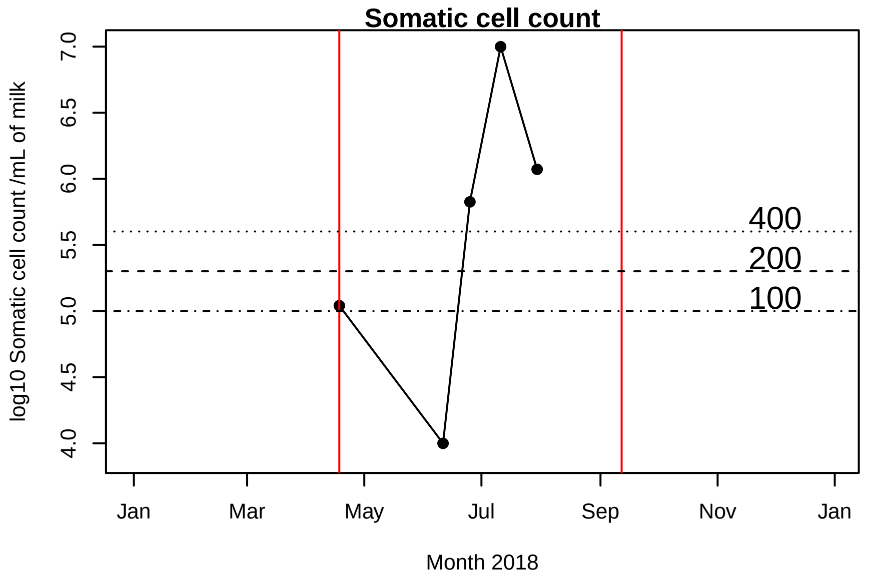

	# quarter	Sampling 1	Sampling 2
**Aerococcus viridans**	3	3	0
**Staphylococccus haemolyticus**	1	0	1
**Staphylococcus epidermidis**	3	12	0
**Staphylococcus haemolyticus**	4	4	4

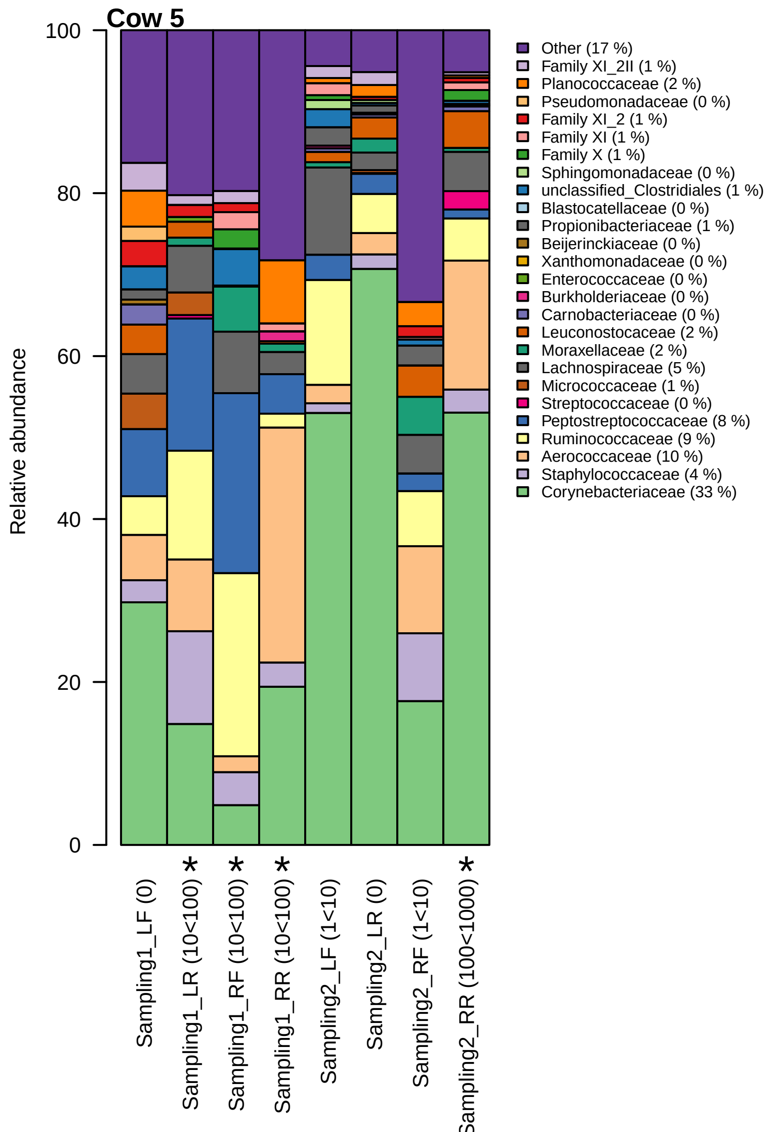

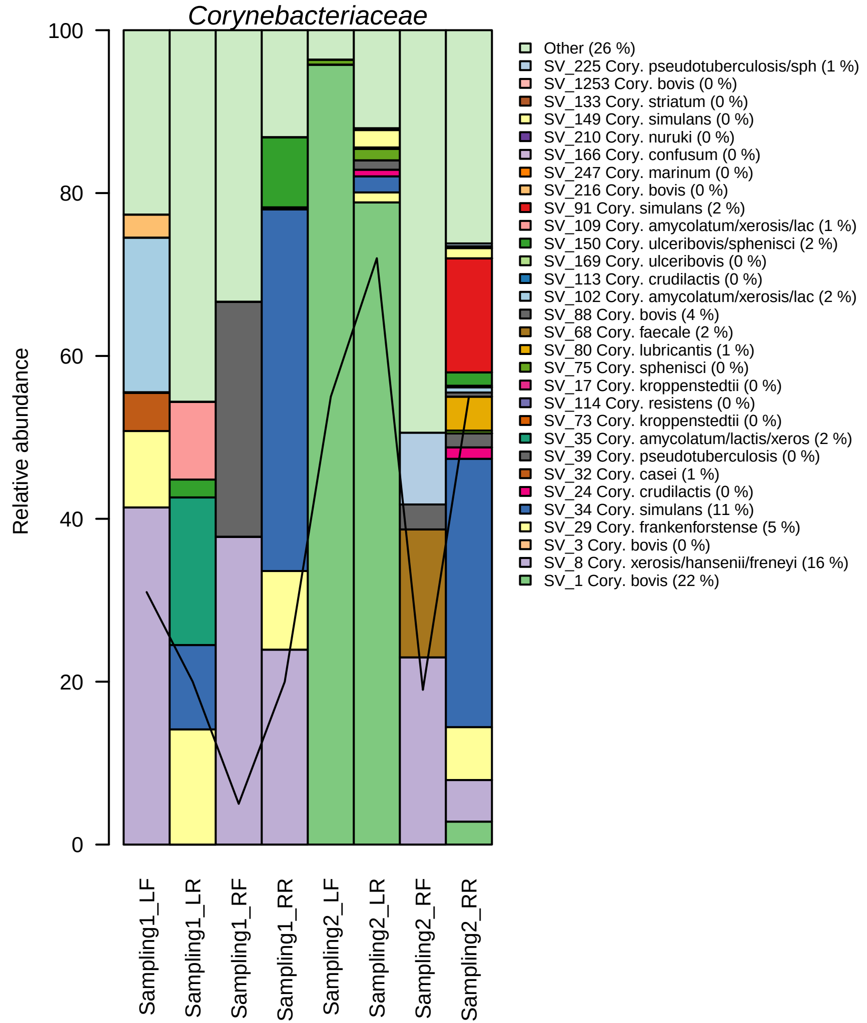

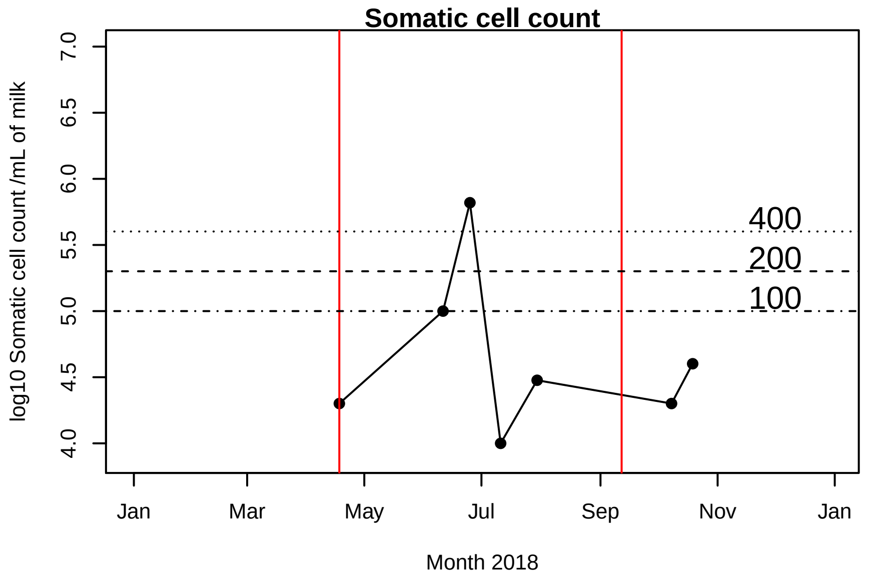

	# quarter	Sampling 1	Sampling 2
**Aerococcus viridans**	3	2	3
**Bacillus pumilus**	1	1	0
**Corynebacterium amycolatum**	1	1	0
**Pediococcus pentosaceus**	2	1	1
**Staphylococcus epidermidis**	3	4	1
**Staphylococcus hominis**	1	1	0
**Staphylococcus microti**	1	1	0
**Staphylococcus saprophyticus**	1	0	1
**Staphylococcus sciuri**	1	0	1
**Staphylococcus xylosus**	1	1	0
**Streptococcus lutetiensis**	1	0	1

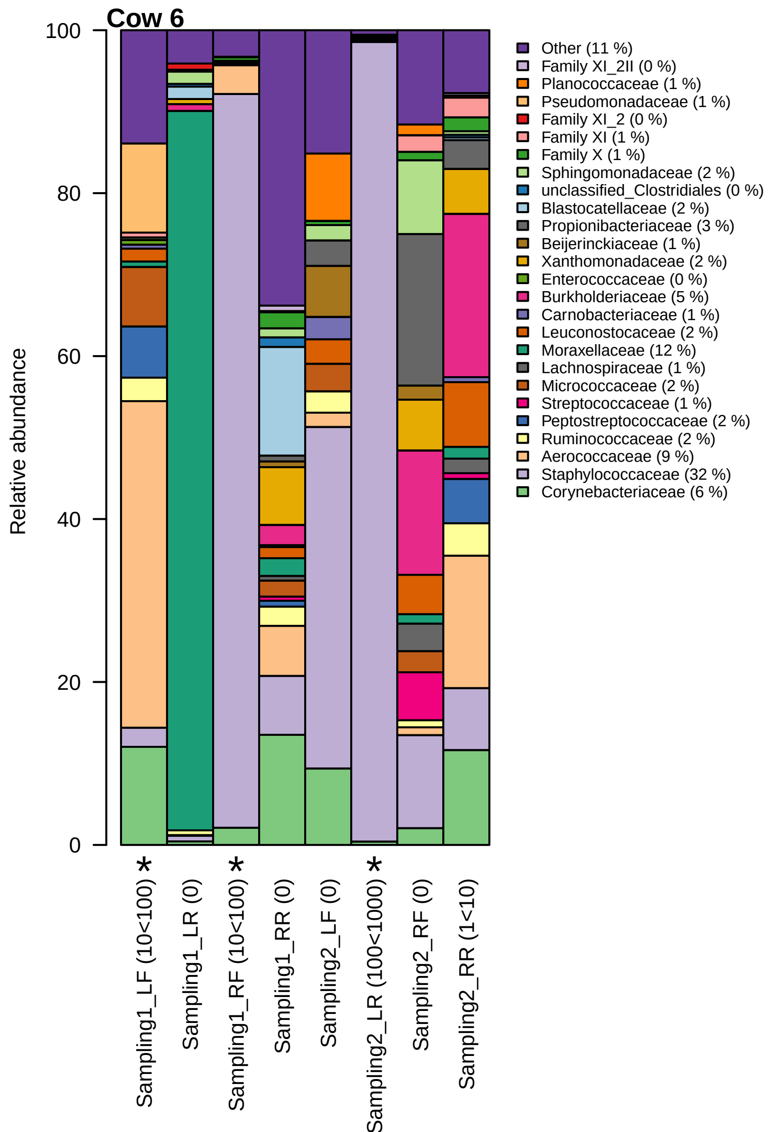

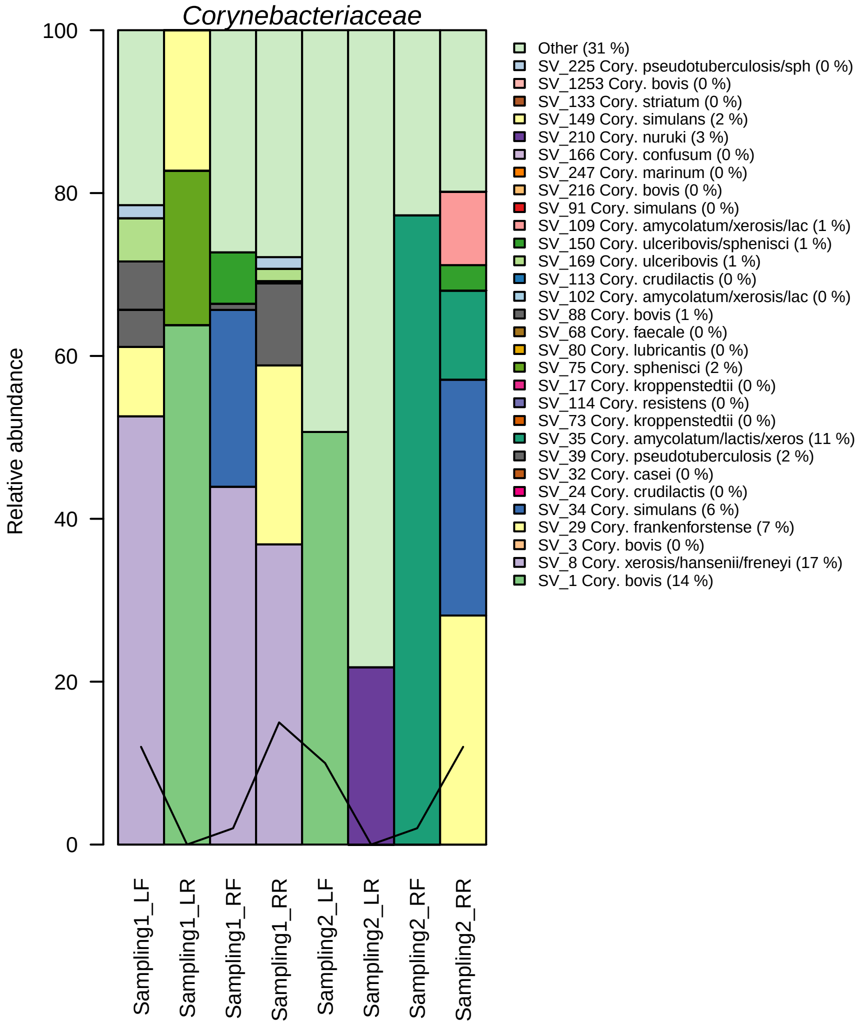

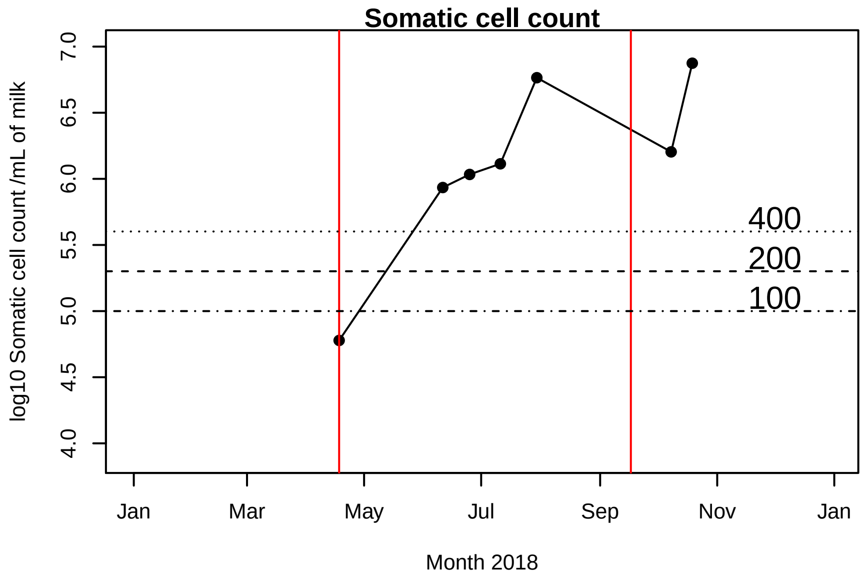

	# quarter	Sampling 1	Sampling 2
**Aerococcus viridans**	4	3	1
**Bacillus pumilus**	1	1	0
**Pediococcus pentosaceus**	1	1	0
**Staphylococcus epidermidis**	3	4	4
**Staphylococcus haemolyticus**	2	1	1

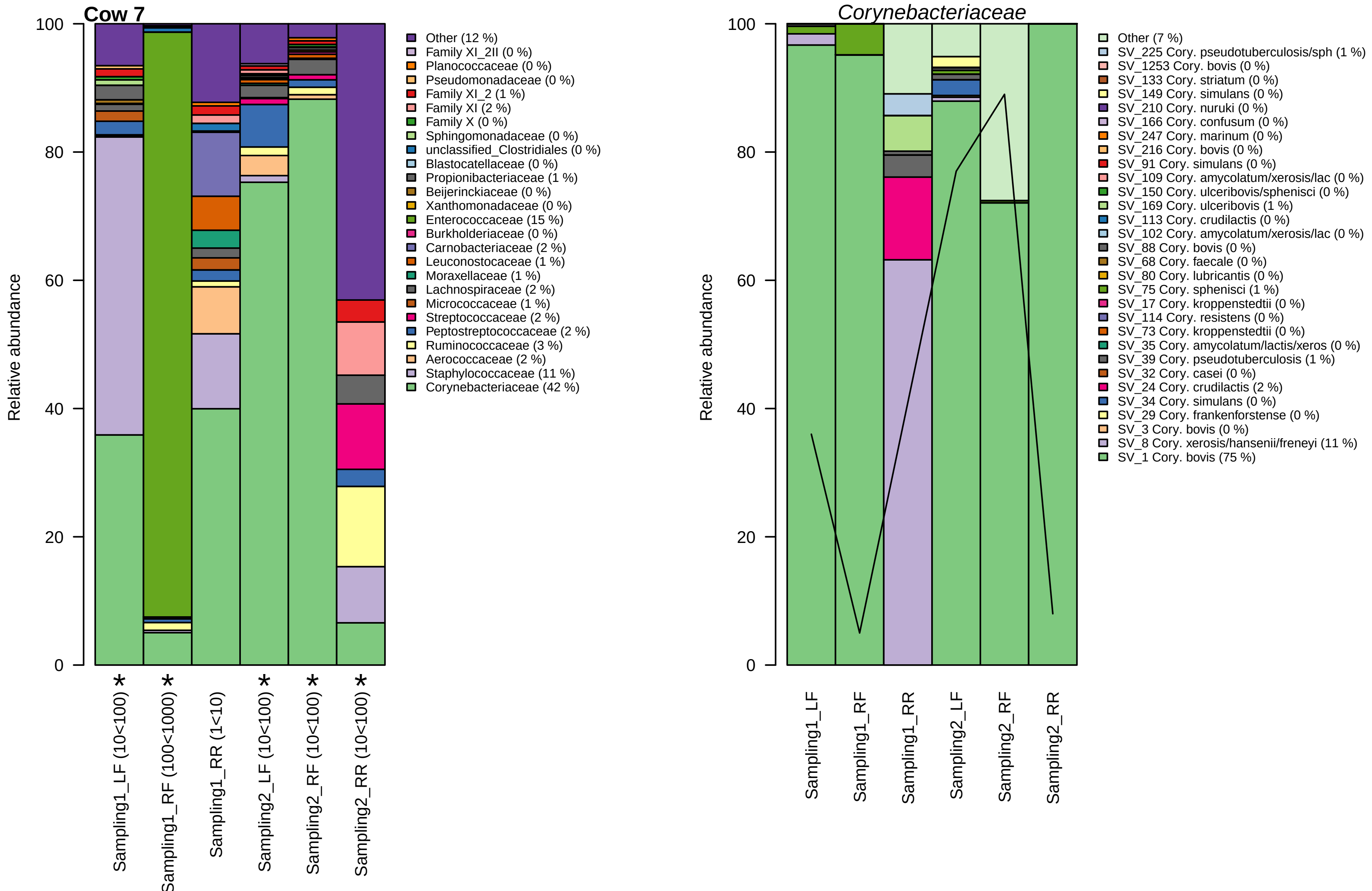

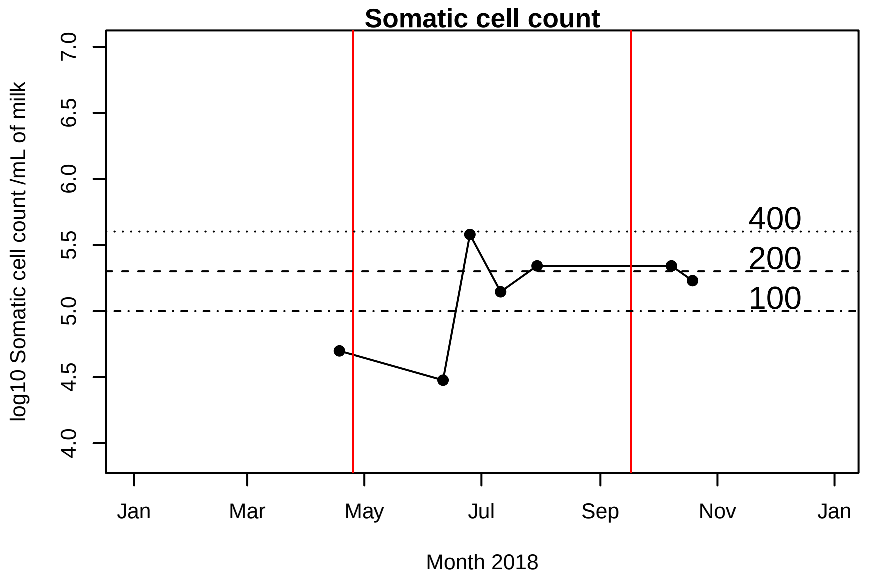

	# quarter	Sampling 1	Sampling 2
**Aerococcus viridans**	4	3	3
**Bacillus licheniformis**	1	1	0
**Bacillus pumilus**	1	1	0
**Corynebacterium bovis**	1	0	1
**Enterococcus gallinarum**	1	6	0
**Staphylococcus chromogenes**	2	1	2
**Staphylococcus epidermidis**	4	10	5
**Staphylococcus hominis**	2	0	2
**Streptococcus lutetiensis**	2	0	3

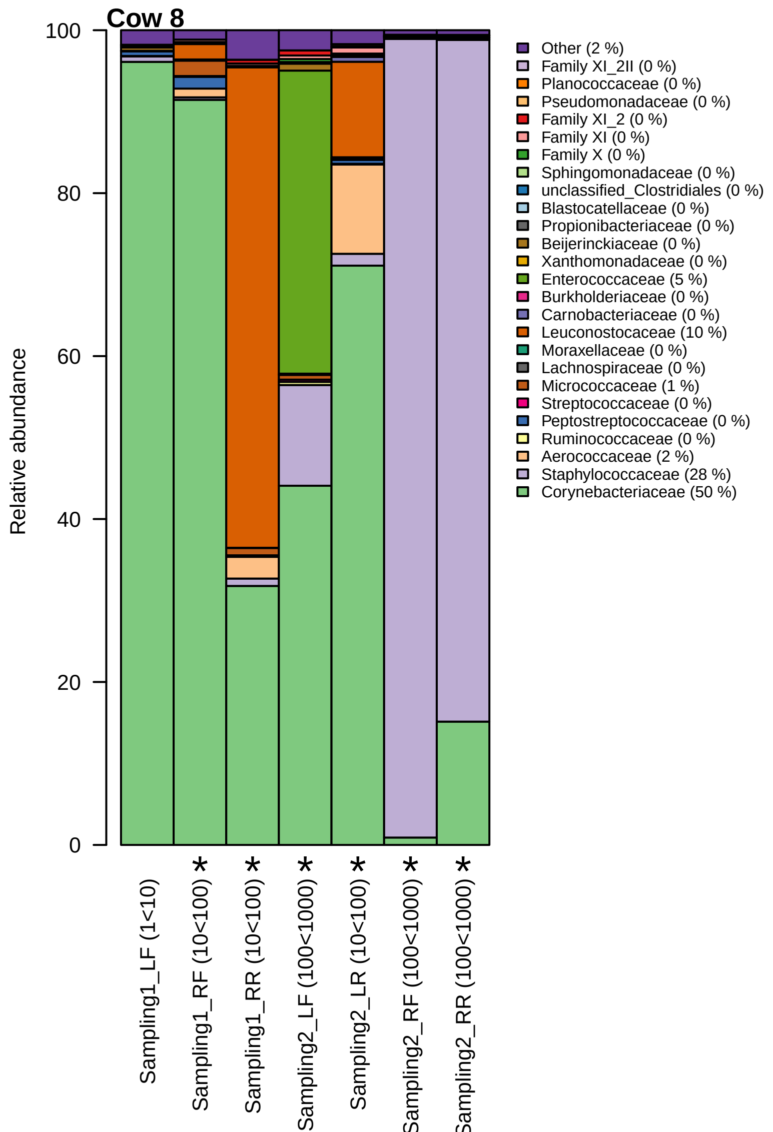

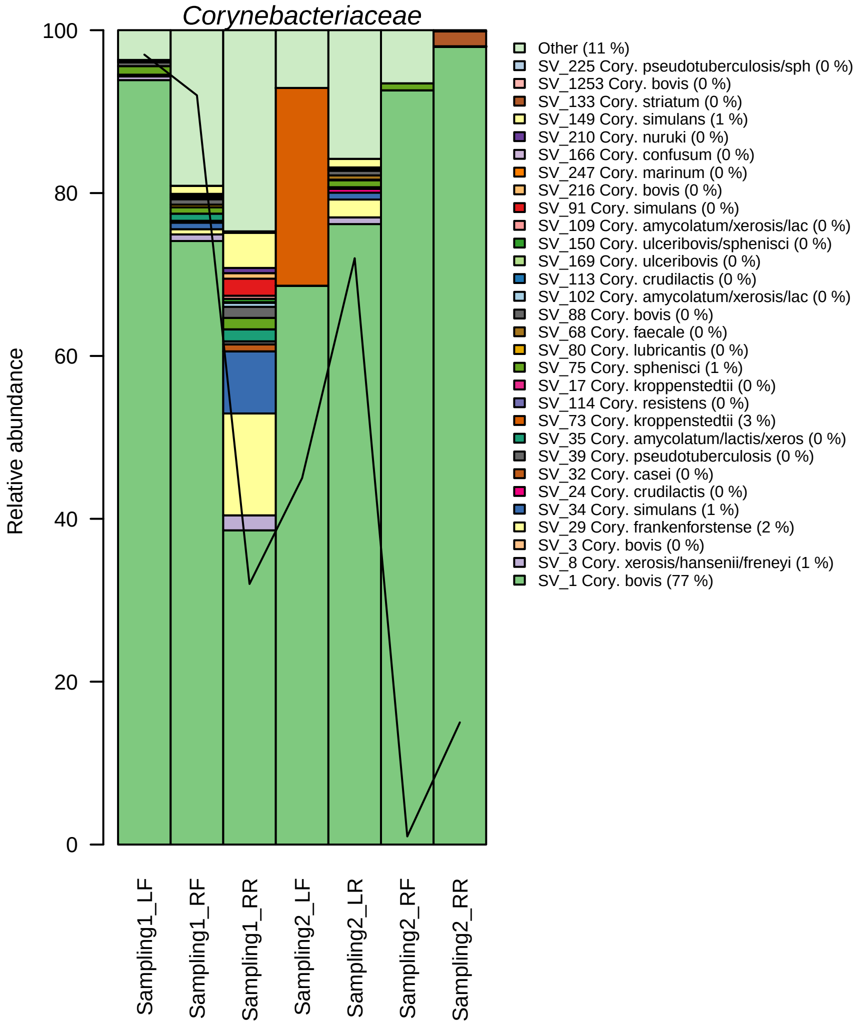

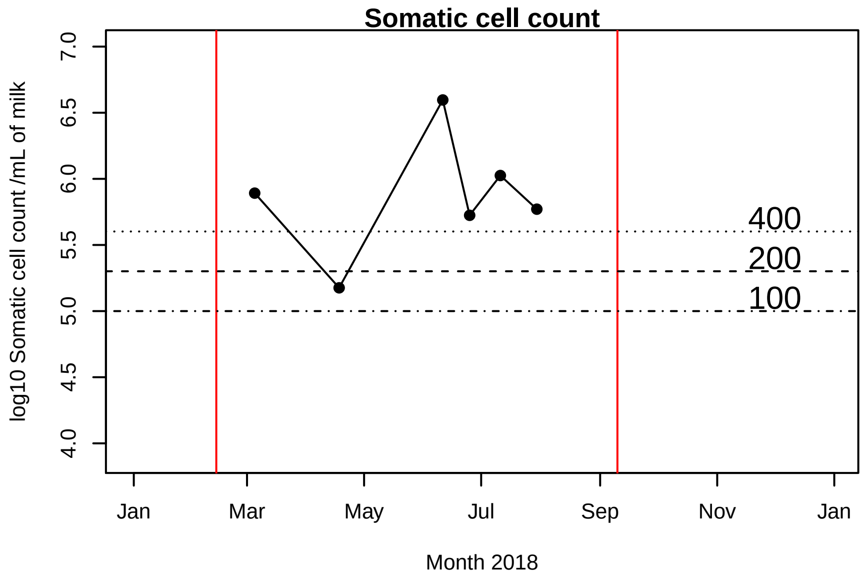

	# quarter	Sampling 1	Sampling 2
**Aerococcus viridans**	3	4	2
**Corynebacterium amycolatum**	1	1	0
**Corynebacterium sp**	1	1	0
**Enterococcus gallinarum**	1	0	2
**Raoultella ornithinolytica**	1	1	0
**Staphylococcus epidermidis**	5	4	10
**Staphylococcus equorum**	1	1	0
**Staphylococcus haemolyticus**	1	1	0
**Streptococcus parauberis**	1	1	0
**Streptococcus uberis**	1	3	0

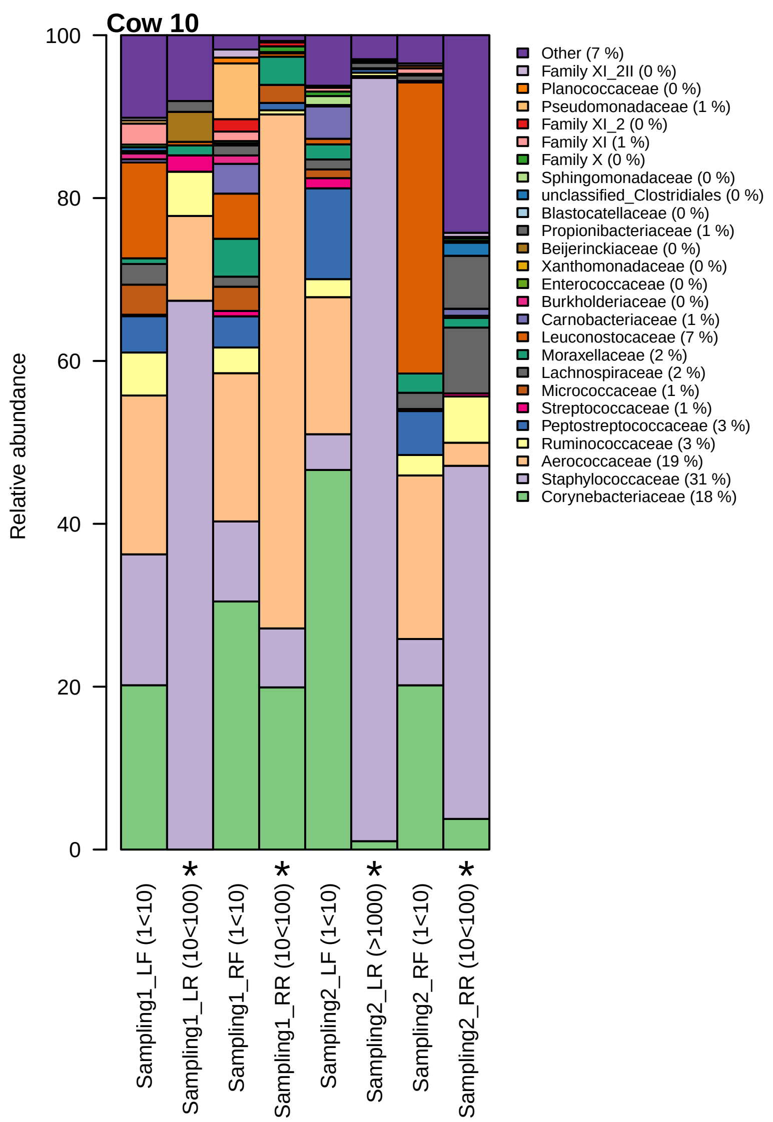

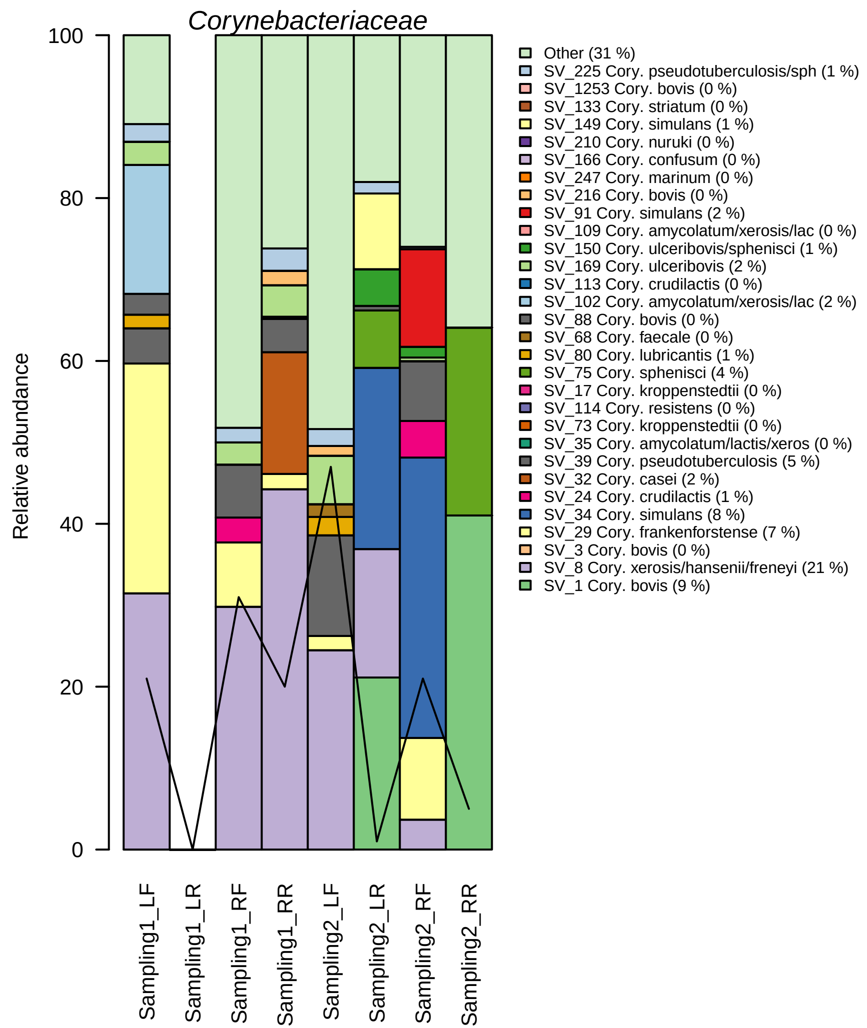

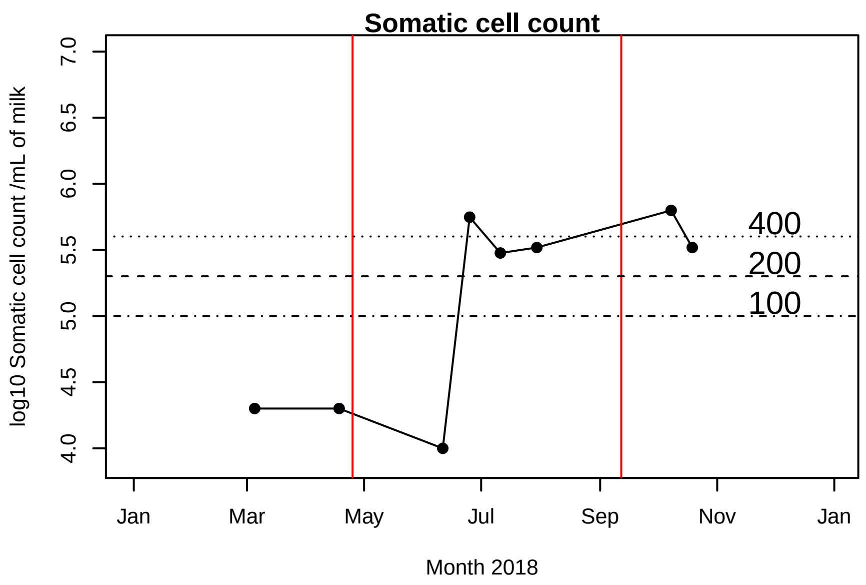

	# quarter	Sampling 1	Sampling 2
**Aerococcus viridans**	6	8	2
**Bacillus cereus**	1	0	1
**Bacillus pumilus**	1	1	0
**Pediococcus pentosaceus**	1	1	0
**Staphylococcus chromogenes**	2	4	4
**Staphylococcus epidermidis**	5	9	5
**Staphylococcus haemolyticus**	1	0	1
**Staphylococcus hominis**	2	0	2

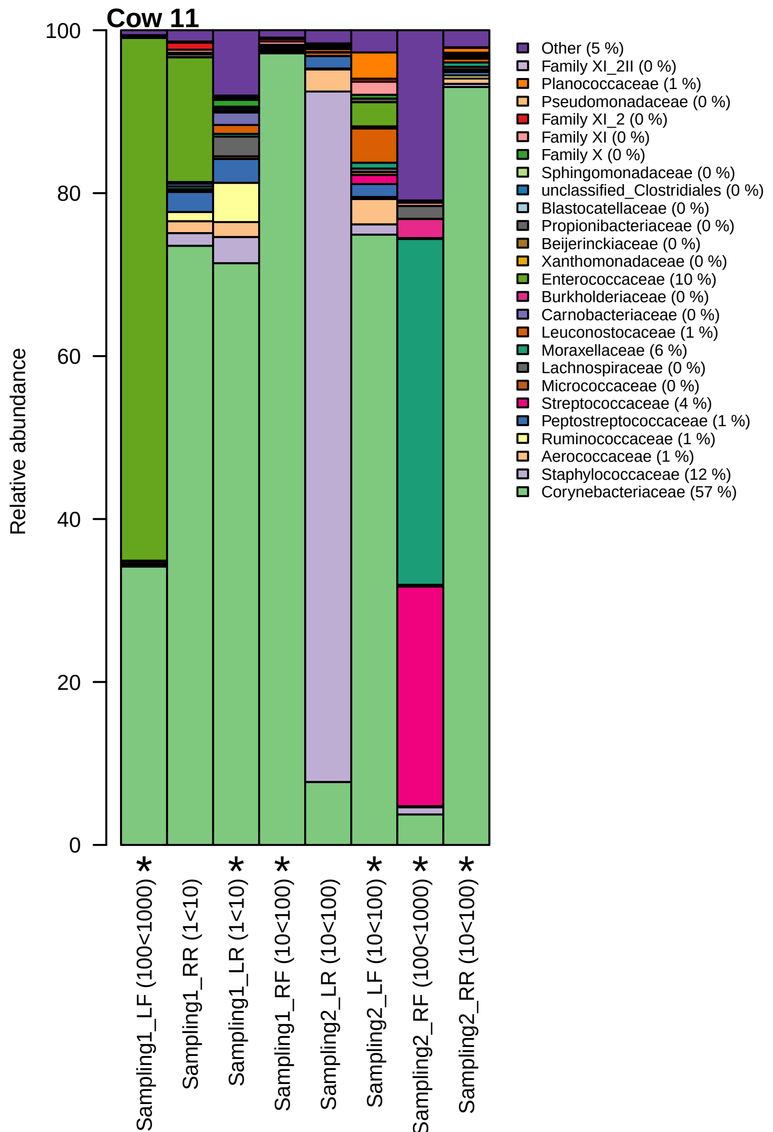

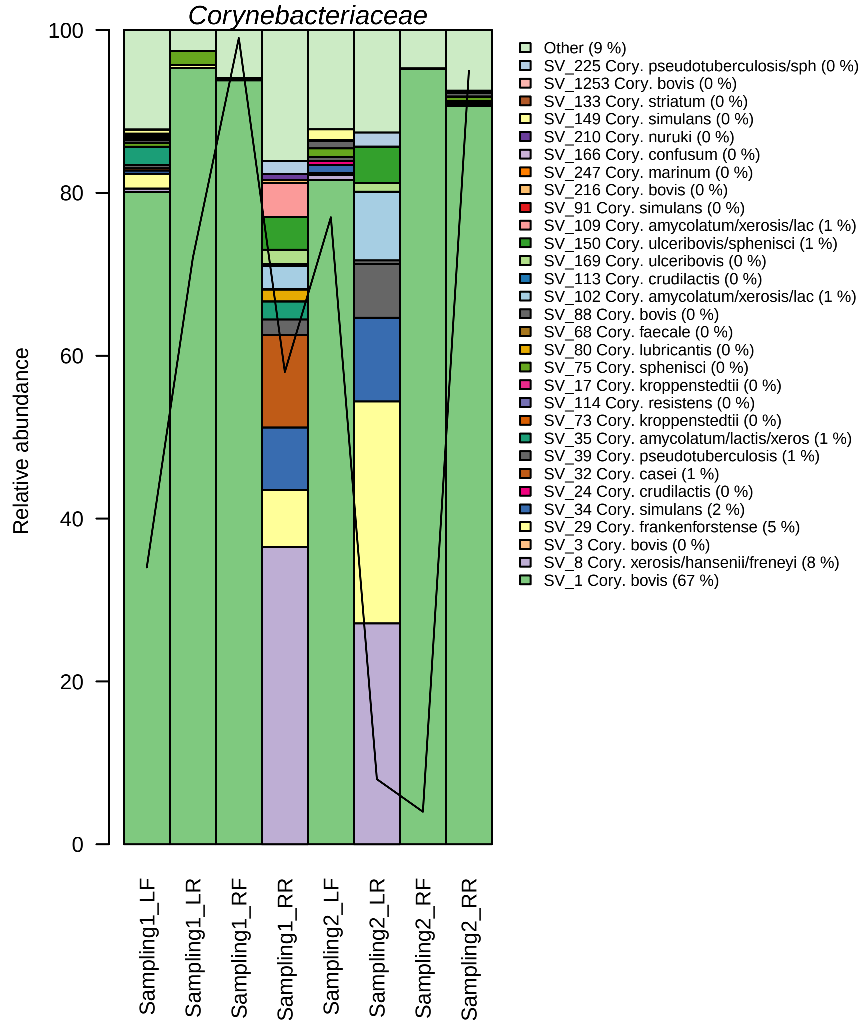

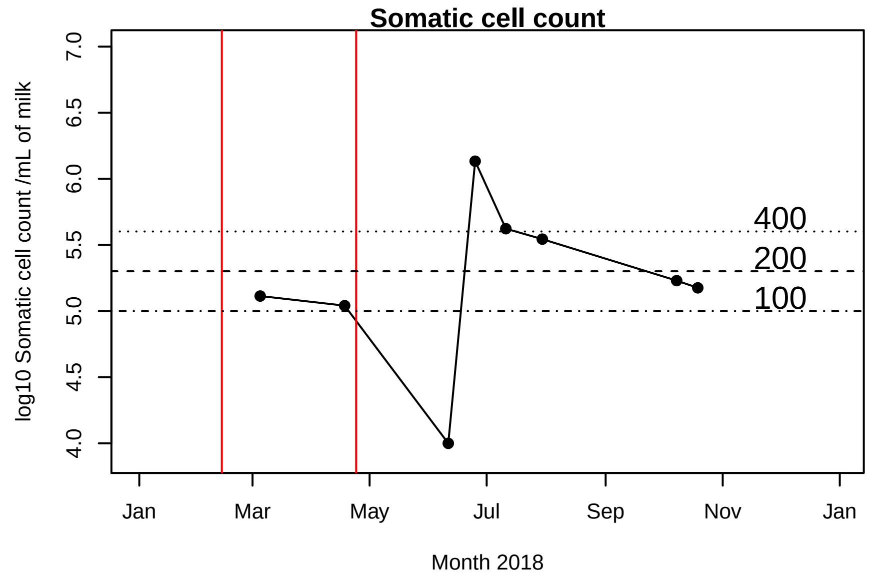

	# quarter	Sampling 1	Sampling 2
**Aerococcus viridans**	5	4	4
**Aeromonas hydrophila**	1	0	1
**Bacillus pumilus**	1	1	0
**Citrobacter freundii**	1	0	1
**Corynebacterium amycolatum**	1	1	0
**Corynebacterium bovis**	1	0	1
**Enterococcus gallinarum**	4	9	3
**Enterococcus malodoratus**	1	1	0
**Lactobacillus plantarum**	1	1	0
**Lactococcus raffinolactis**	1	0	1
**Staphylococcus epidermidis**	3	5	0
**Staphylococcus equorum**	1	0	1
**Staphylococcus warneri**	1	1	0
**Staphylococcus xylosus**	2	11	0
**Streptococcus parauberis**	1	0	2

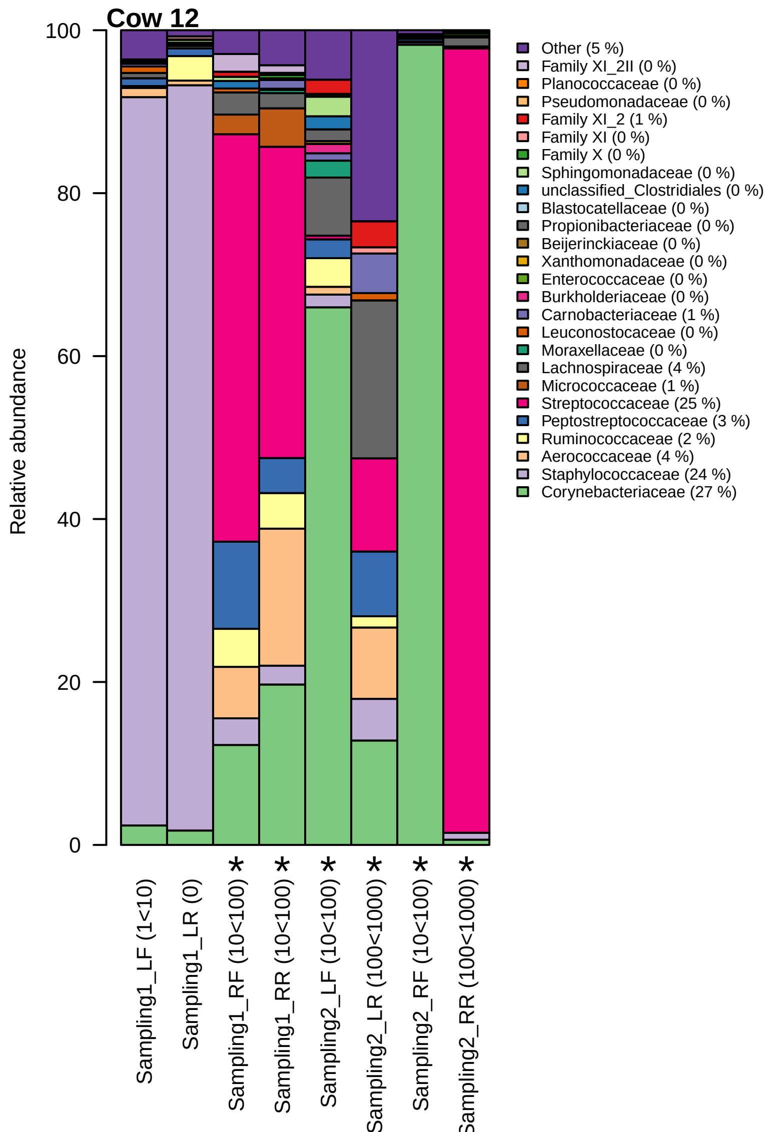

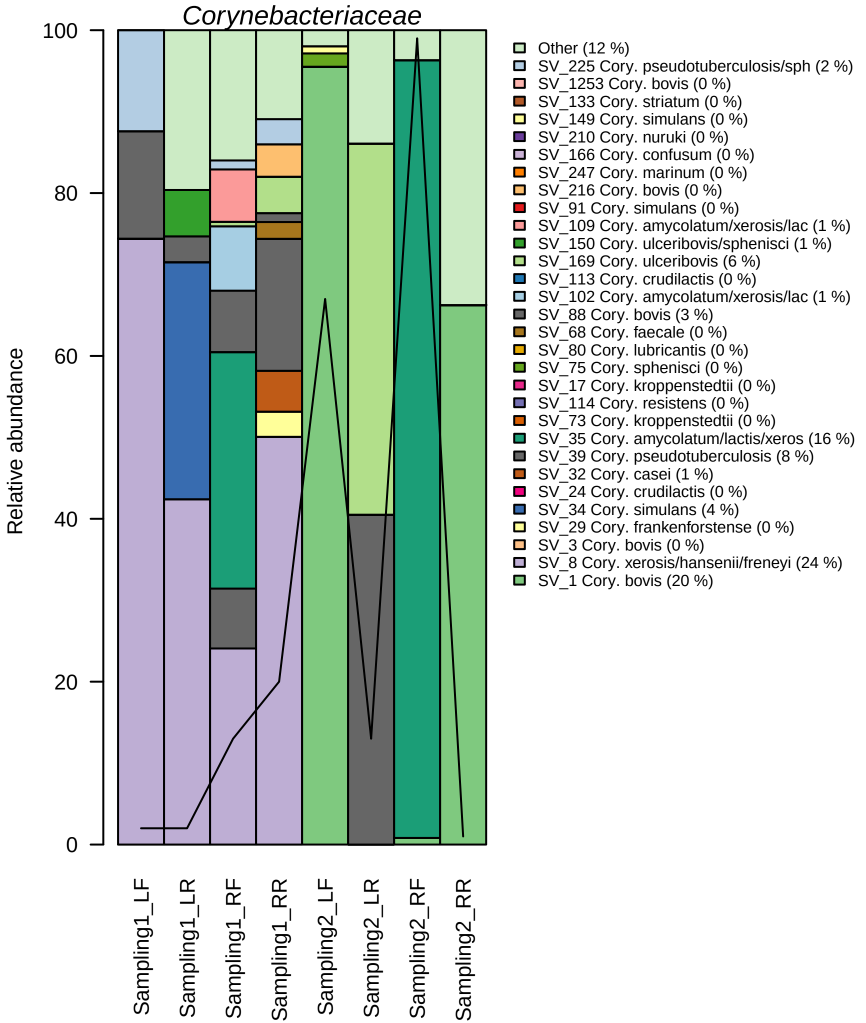

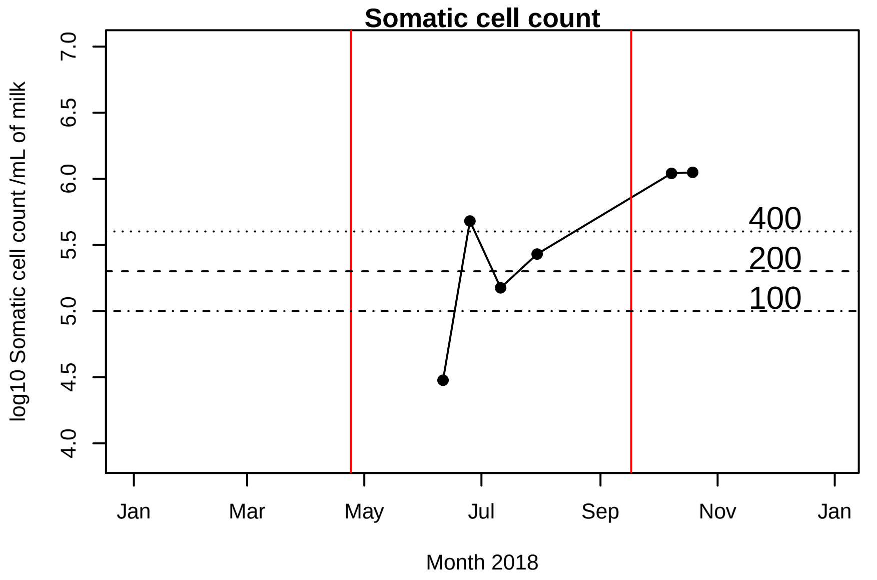

	# quarter	Sampling 1	Sampling 2
**Aerococcus viridans**	4	3	2
**Bacillus licheniformis**	1	1	0
**Bacillus pumilus**	1	1	0
**Corynebacterium amycolatum**	3	1	2
**Corynebacterium bovis**	1	0	1
**Enterococcus faecalis**	2	4	0
**Pediococcus pentosaceus**	1	1	0
**Staphylococcus aureus**	1	0	1
**Staphylococcus epidermidis**	3	5	0
**Staphylococcus haemolyticus**	1	0	1
**Staphylococcus warneri**	1	1	0
**Streptococcus canis**	4	4	3
**Streptococcus dysgalactiae**	1	0	1

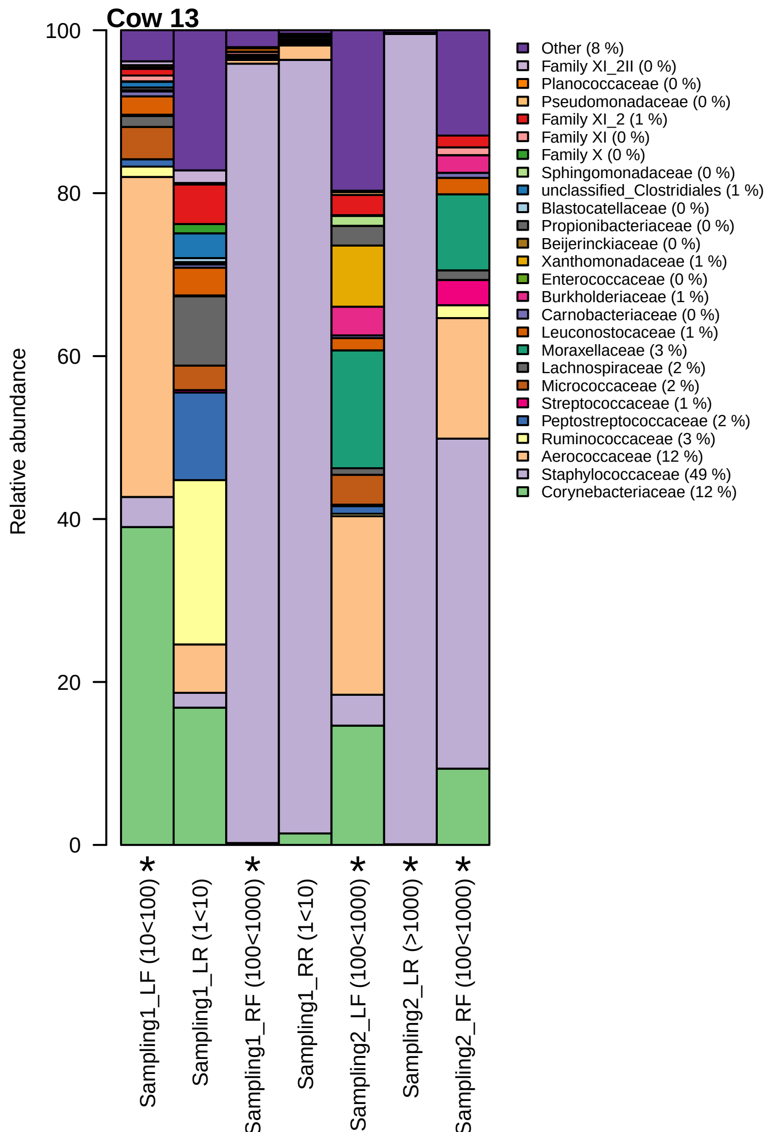

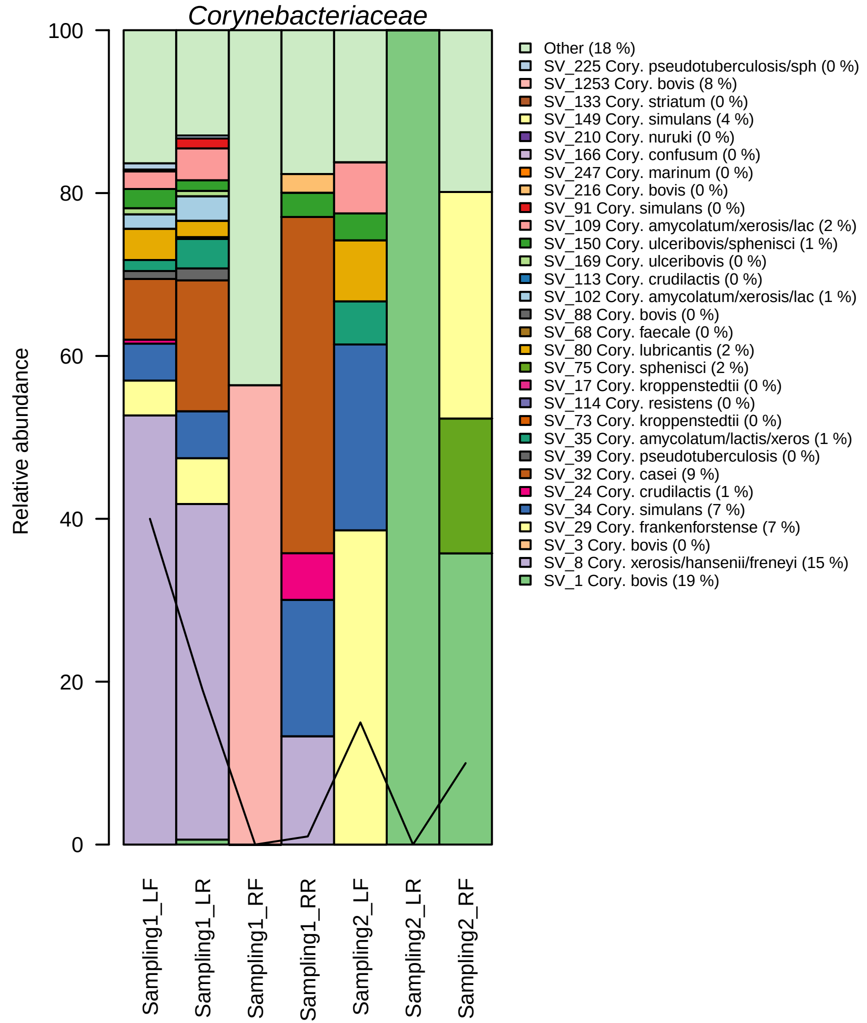

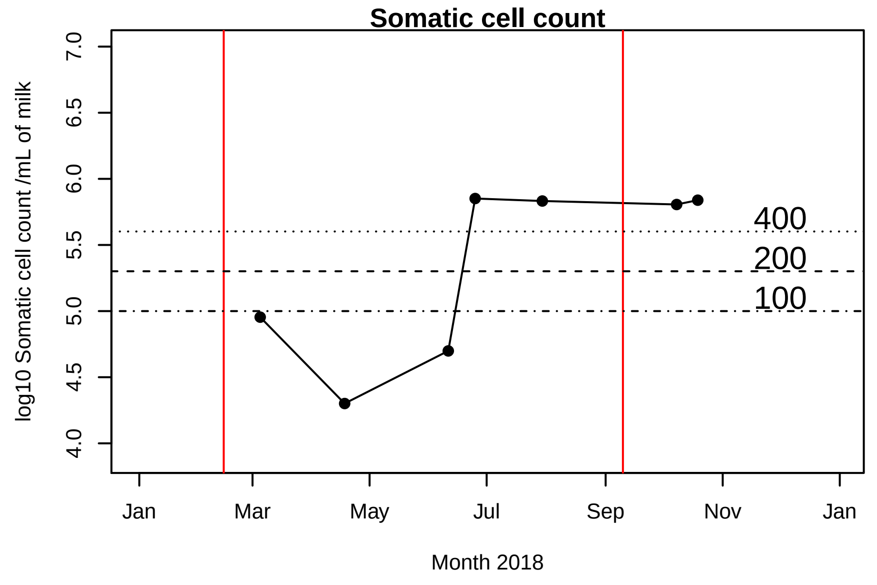

	# quarter	Sampling 1	Sampling 2
**Aerococcus viridans**	5	3	9
**Bacillus cereus**	1	0	1
**Bacillus licheniformis**	2	2	0
**Citrobacter gillenii**	1	0	2
**Corynebacterium amycolatum**	2	1	1
**Corynebacterium stationis**	1	1	0
**Enterococcus gallinarum**	1	3	0
**Escheria coli**	1	0	2
**Lactobacillus plantarum**	1	0	1
**Lactococcus lactis**	1	1	0
**Staphylococcus epidermidis**	6	12	5
**Staphylococcus haemolyticus**	3	0	3
**Staphylococcus simulans**	2	0	2
**Streptococcus parauberis**	1	0	1
**Tetragenococcus solitarius**	1	1	0

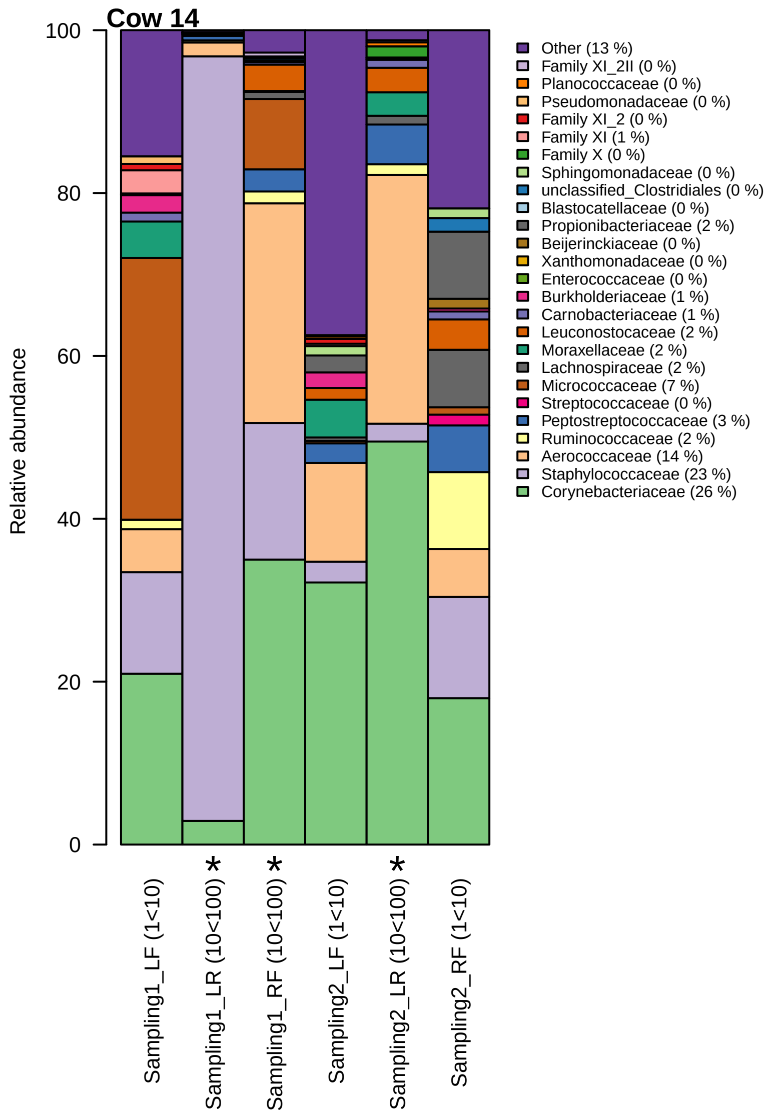

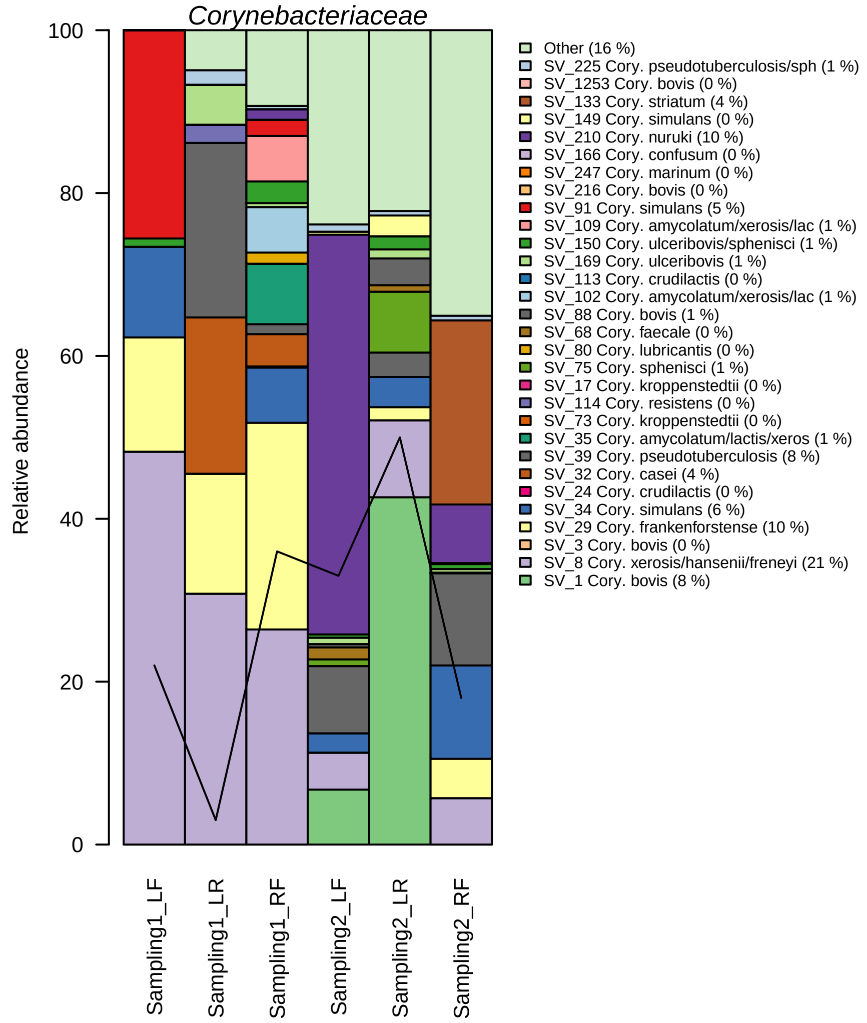

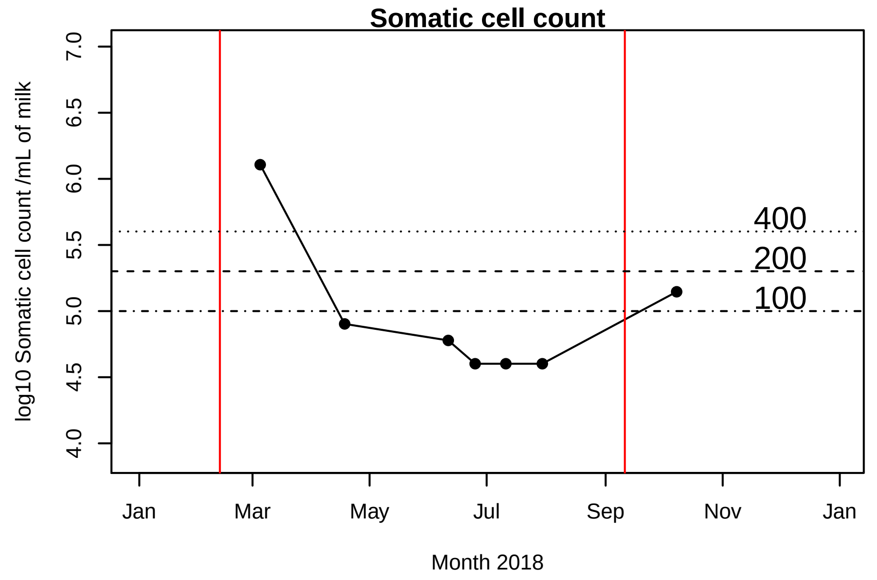

	# quarter	Sampling 1	Sampling 2
**Aerococcus viridans**	4	4	5
**Bacillus licheniformis**	1	1	0
**Bacillus pumilus**	1	1	0
**Corynebacterium amycolatum**	1	1	0
**Staphylococcus chromogenes**	1	2	0
**Staphylococcus epidermidis**	2	2	0
**Staphylococcus haemolyticus**	1	0	1
**Staphylococcus hominis**	1	0	1
**Staphylococcus saprophyticus**	2	0	2
**Staphylococcus sciuri**	1	1	0
**Staphylococcus warneri**	1	1	0

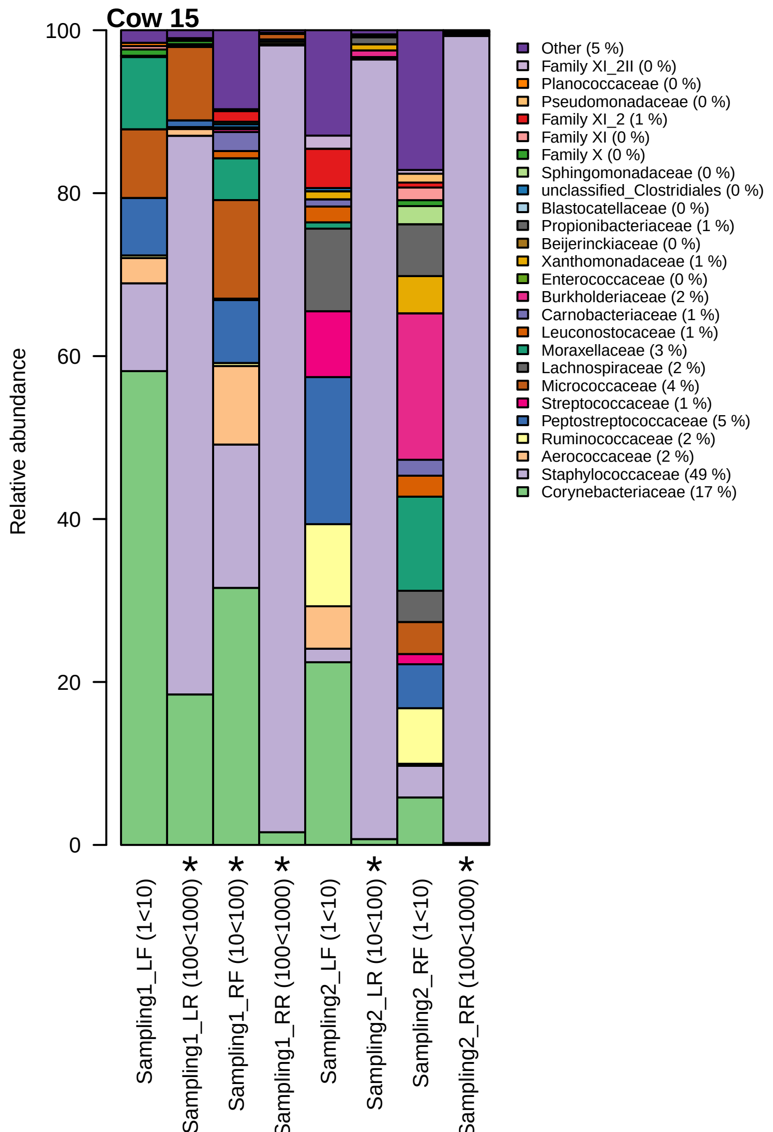

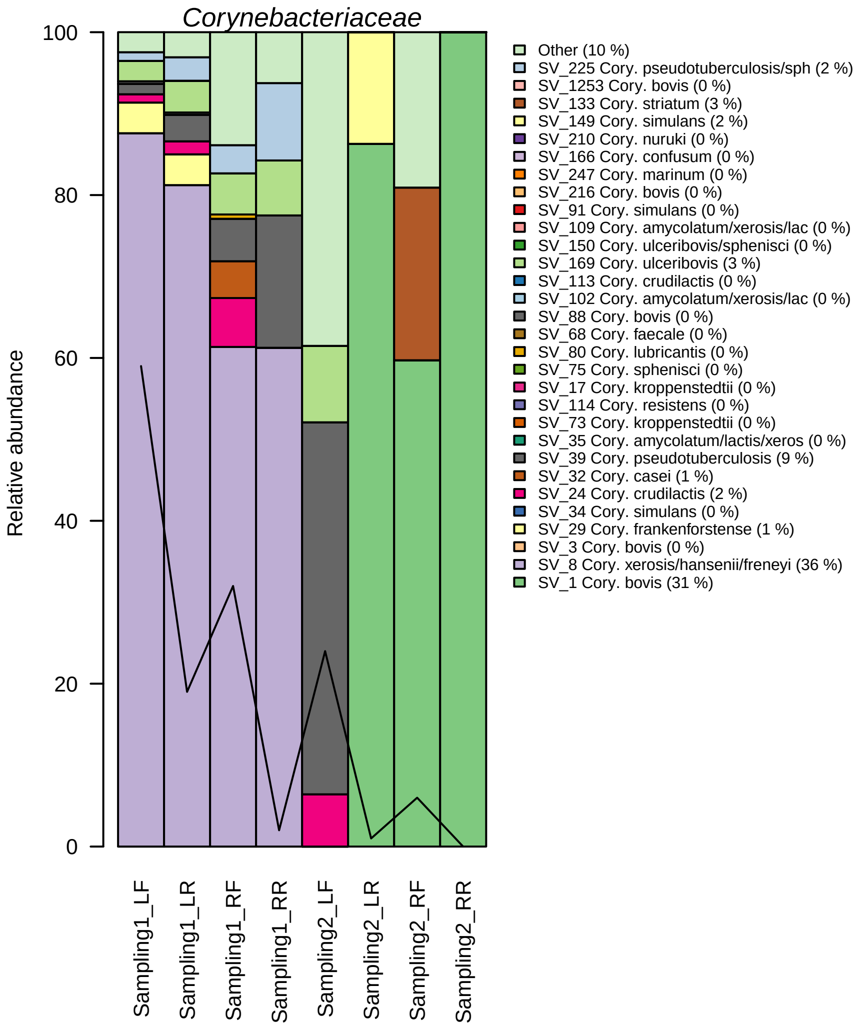

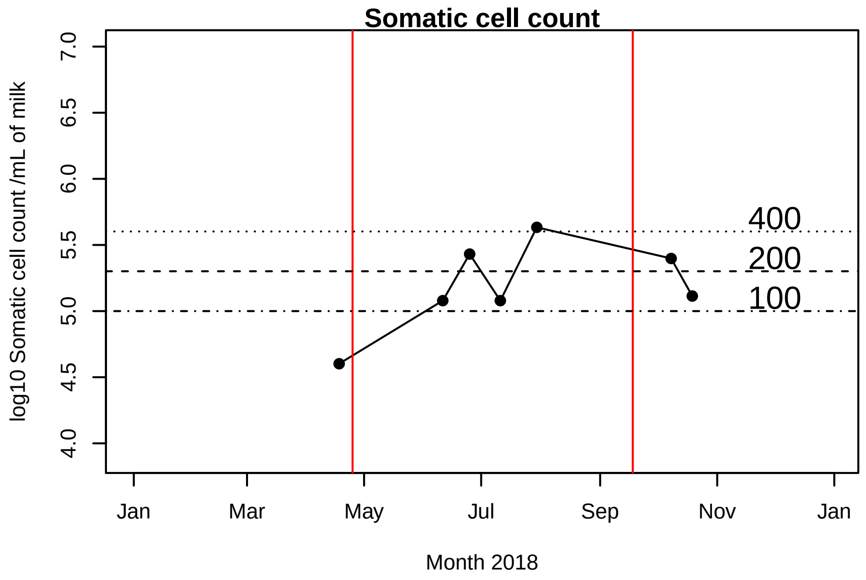

	# quarter	Sampling 1
**Aerococcus viridans**	2	3
**Bacillus licheniformis**	1	2
**Bacillus pumilus**	1	1
**Kluyvera intermedia**	1	3
**Lactococcus lactis**	1	2
**Staphylococcus chromogenes**	2	7
**Staphylococcus equorum**	1	1
**Staphylococcus hominis**	1	1
**Streptococcus parauberis**	1	1

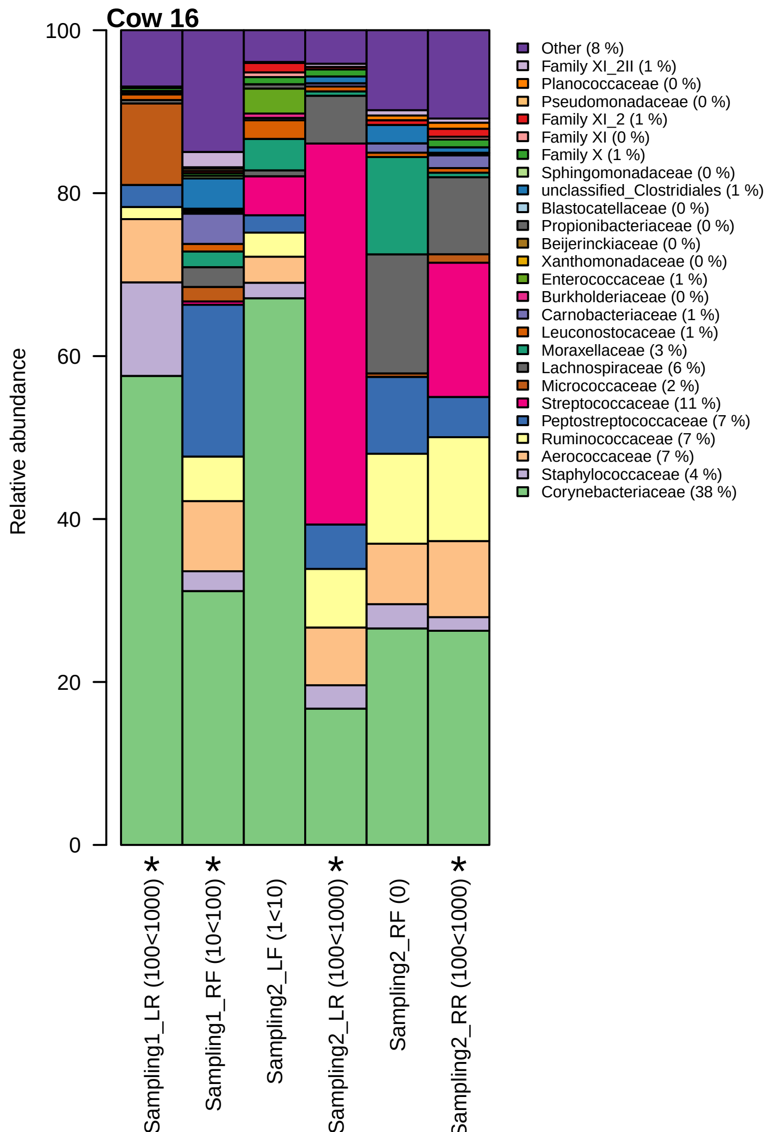

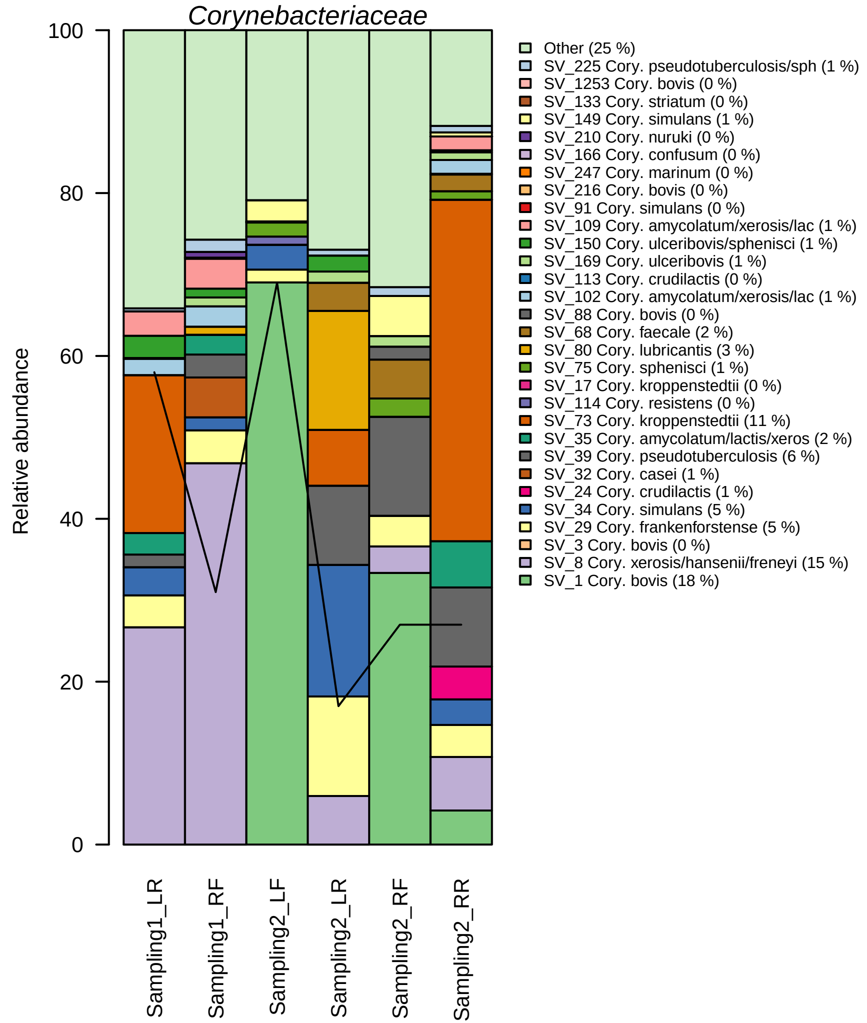

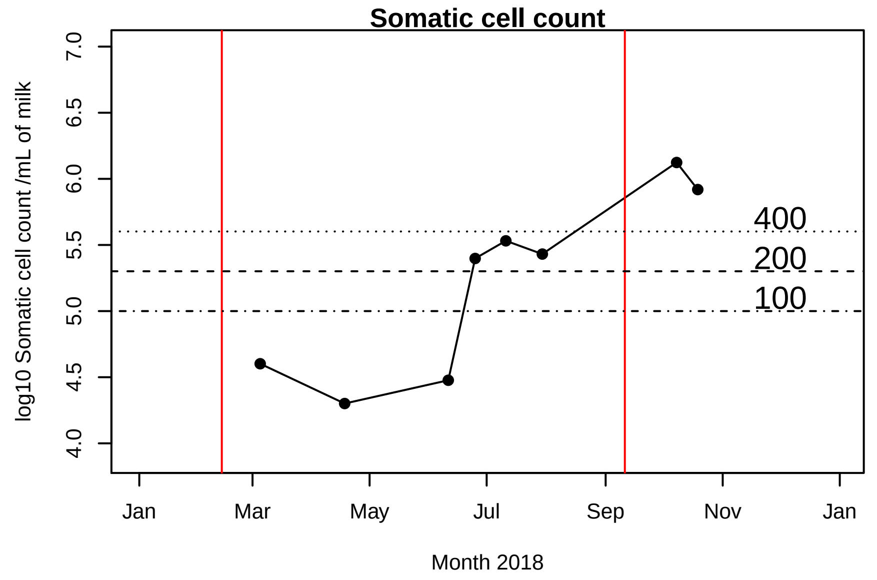

	# quarter	Sampling 1	Sampling 2
**Aerococcus viridans**	5	8	2
**Bacillus licheniformis**	3	3	0
**Bacillus pumilus**	1	1	0
**Citrobacter koseri**	1	1	0
**Corynebacterium freneyi**	1	1	0
**Corynebacterium sp**	1	0	1
**Enterococcus faecalis**	1	5	0
**Lactococcus garvieae**	1	0	2
**Pediococcus pentosaceus**	1	1	0
**Staphylococcus epidermidis**	5	8	4
**Staphylococcus equorum**	2	2	0
**Staphylococcus sciuri**	2	2	0
**Streptococcus dysgalactiae**	1	0	3
**Streptococcus parauberis**	2	1	1
**Streptococcus uberis**	1	0	2

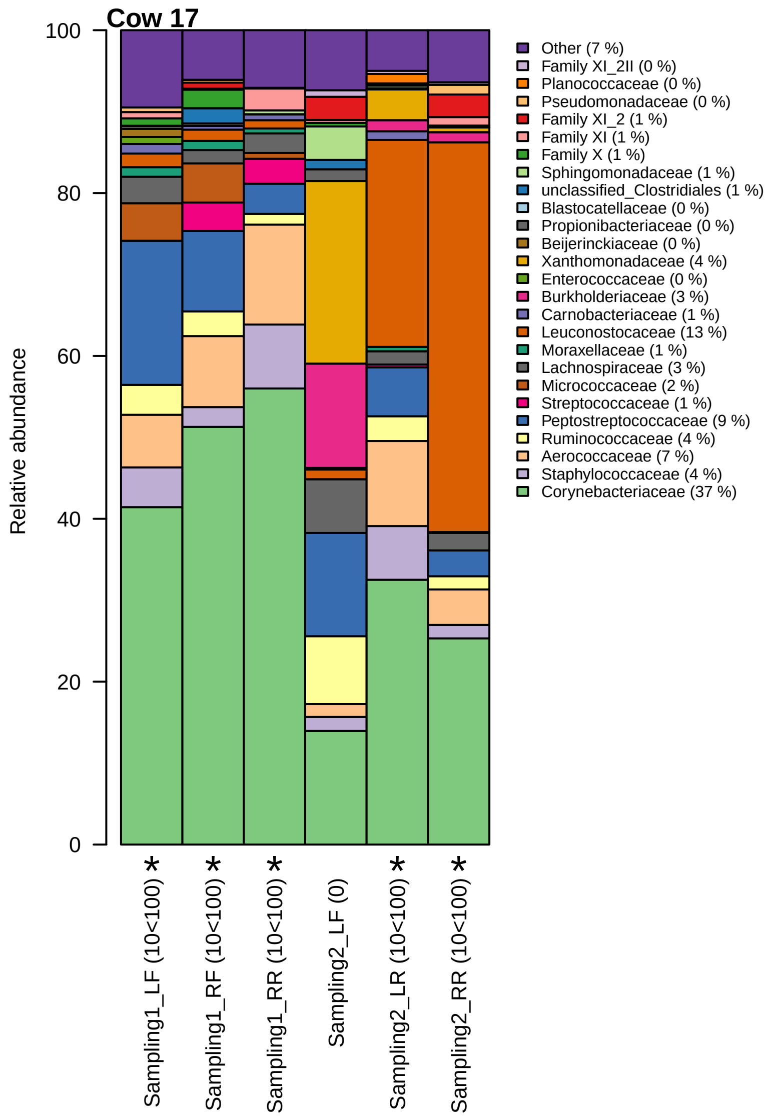

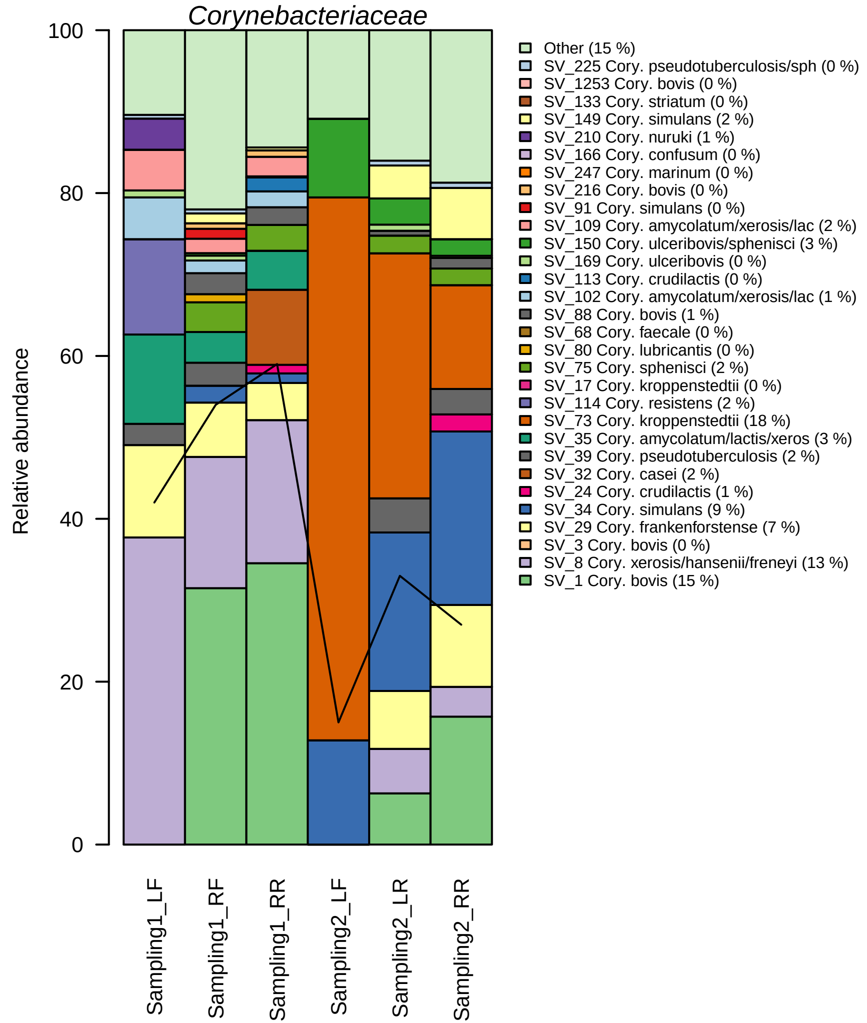

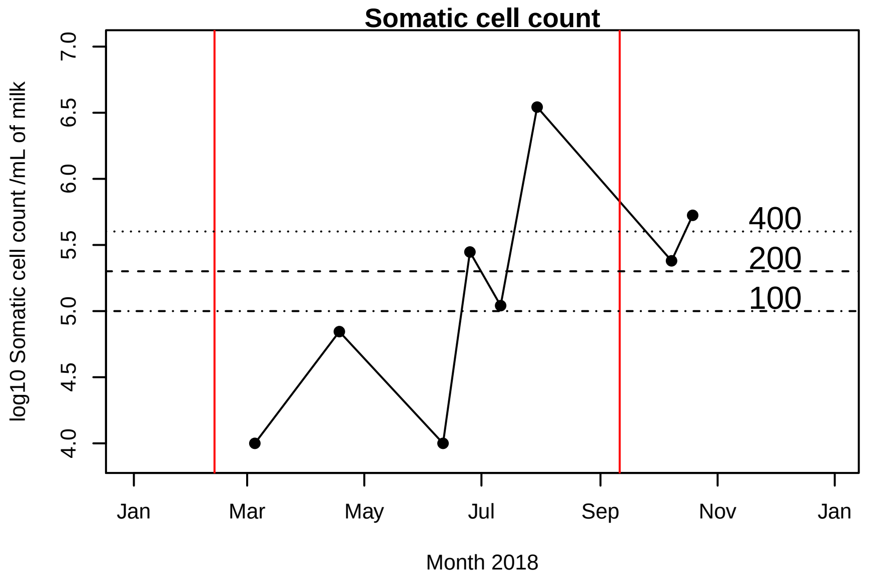

	# quarter	Sampling 1	Sampling 2
**Aerococcus viridans**	5	4	3
**Aeromonas eucrenophila**	1	1	0
**Bacillus pumilus**	2	2	0
**Lactobacillus plantarum**	1	1	0
**Pediococcus pentosaceus**	2	3	0
**Staphylococcus epidermidis**	3	3	0
**Staphylococcus haemolyticus**	2	1	1
**Staphylococcus hominis**	3	1	2
**Staphylococcus piscifermentans**	1	0	1
**Streptococcus parauberis**	1	1	0
**Tetragenococcus solitarius**	1	1	0

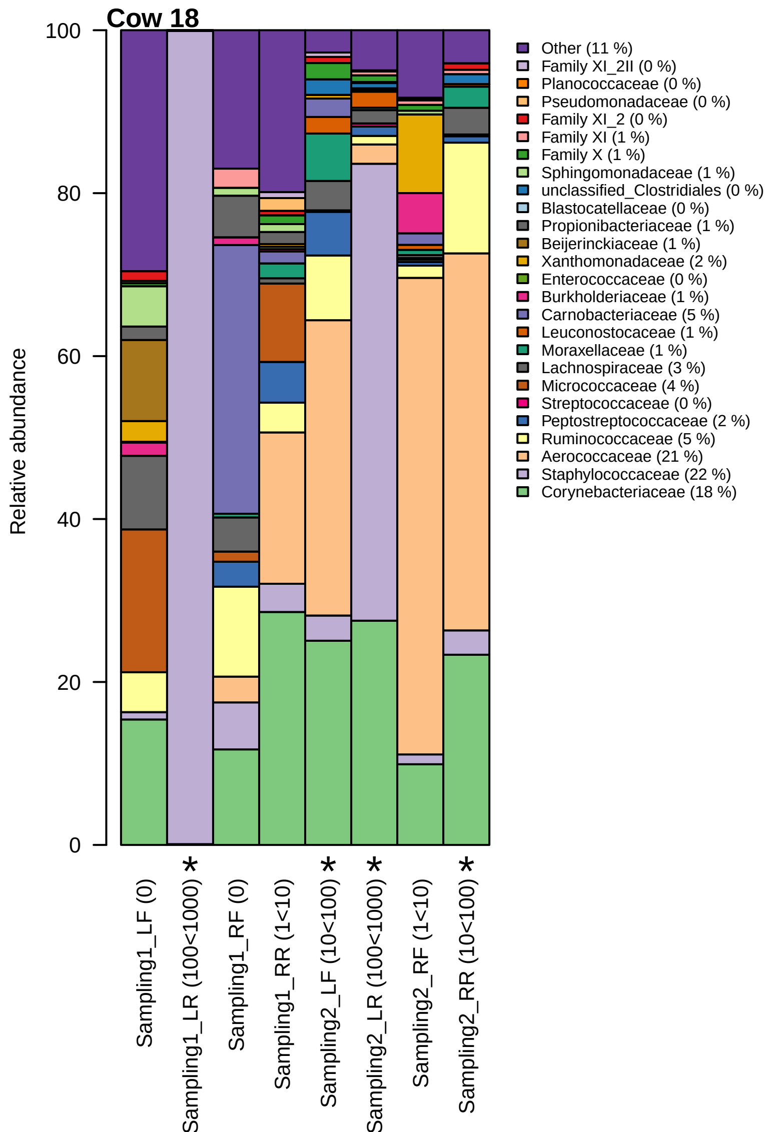

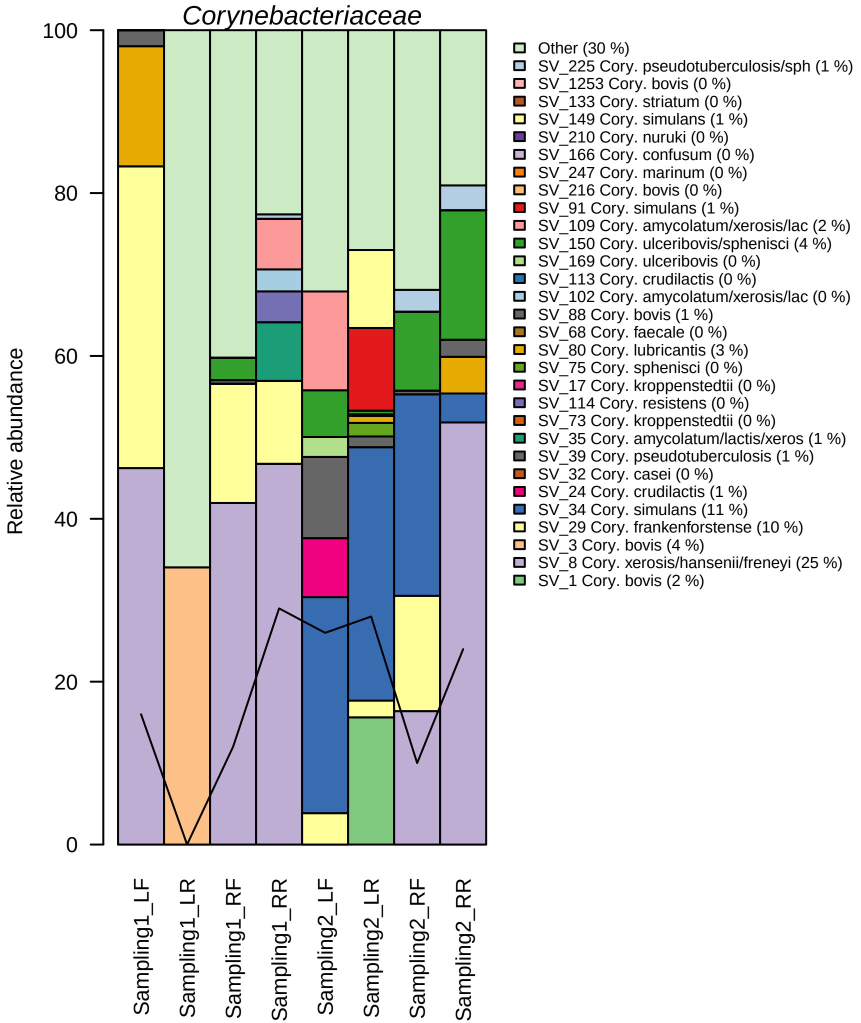

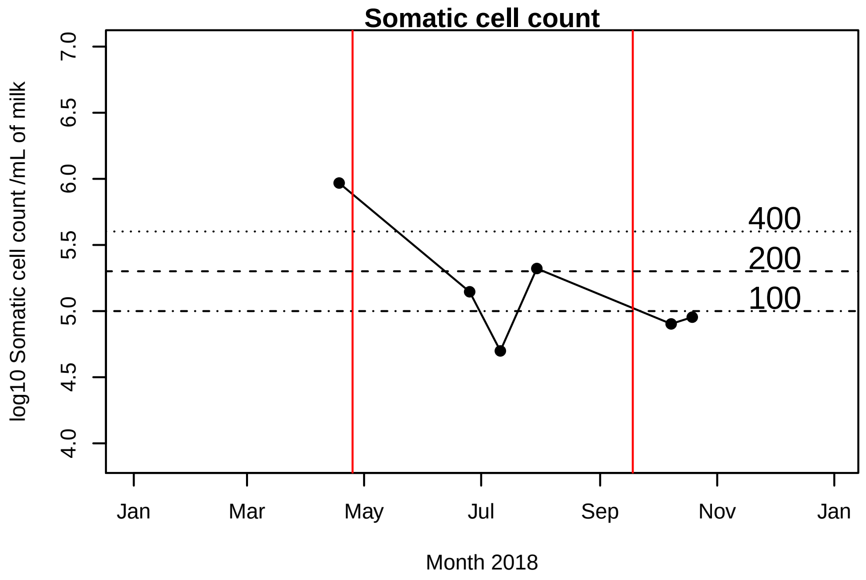

	# quarter	Sampling 1
**Aerococcus viridans**	1	1
**Corynebacterium amycolatum**	1	2
**Corynebacterium aurimucosum**	1	1
**Staphylococcus epidermidis**	3	7
**Streptococcus parauberis**	1	1

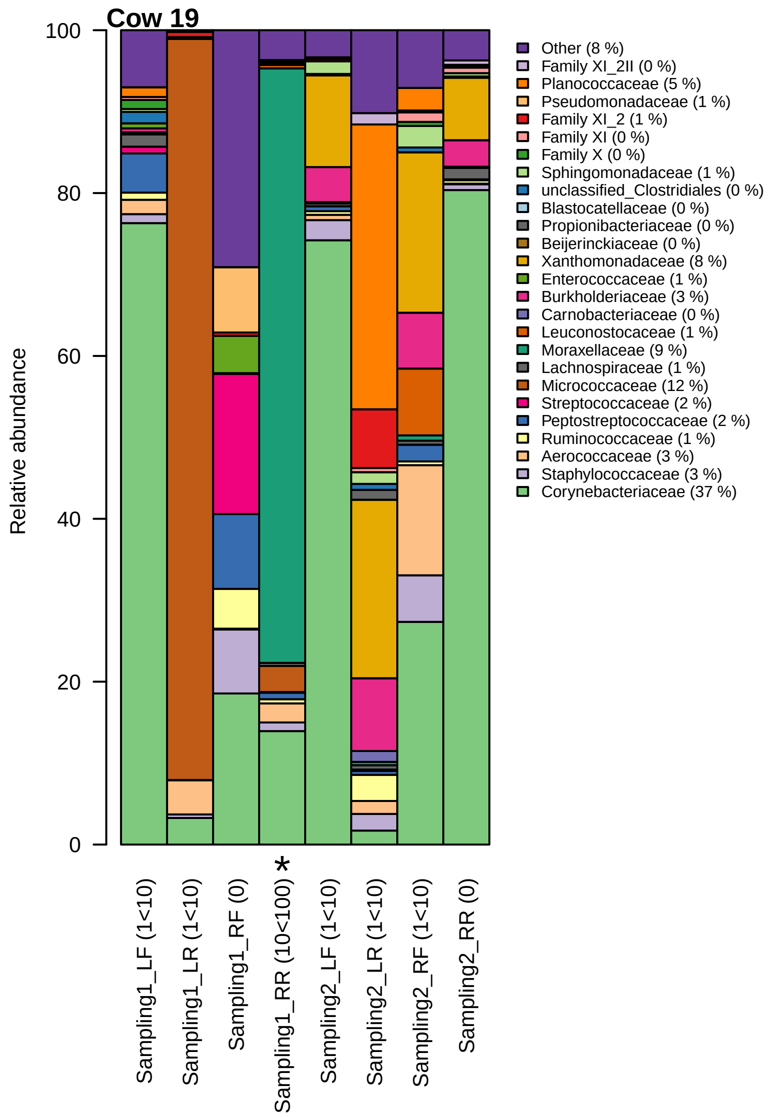

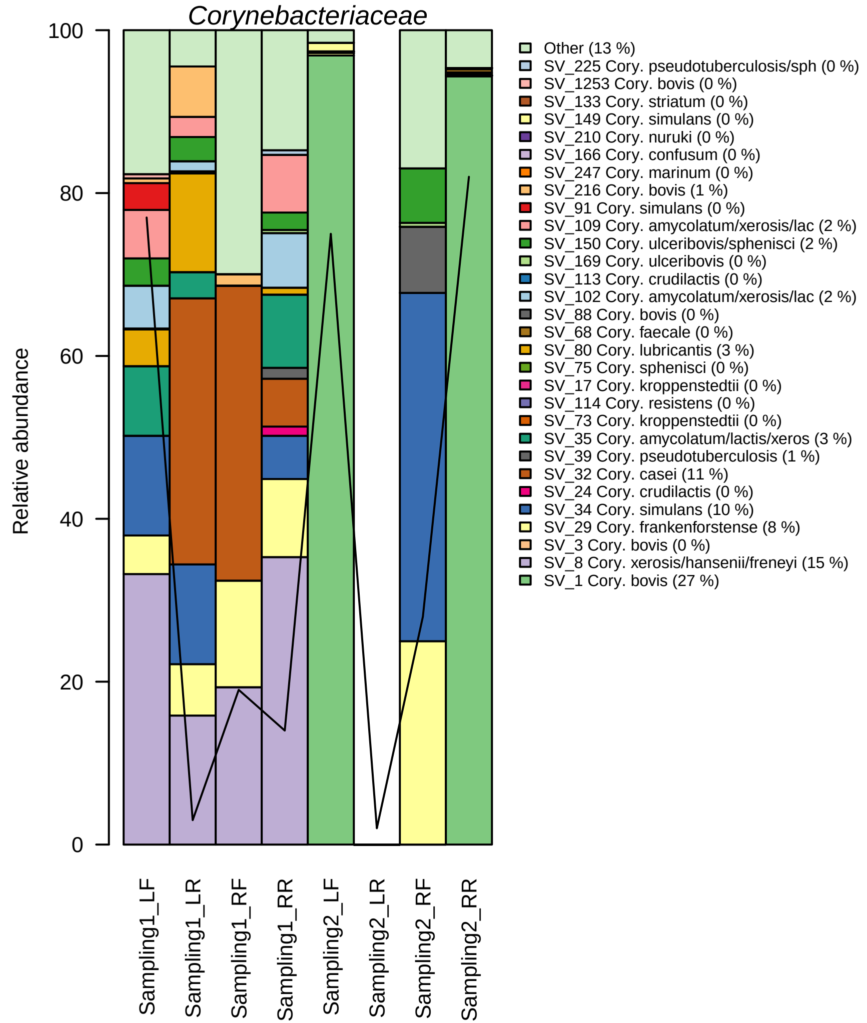

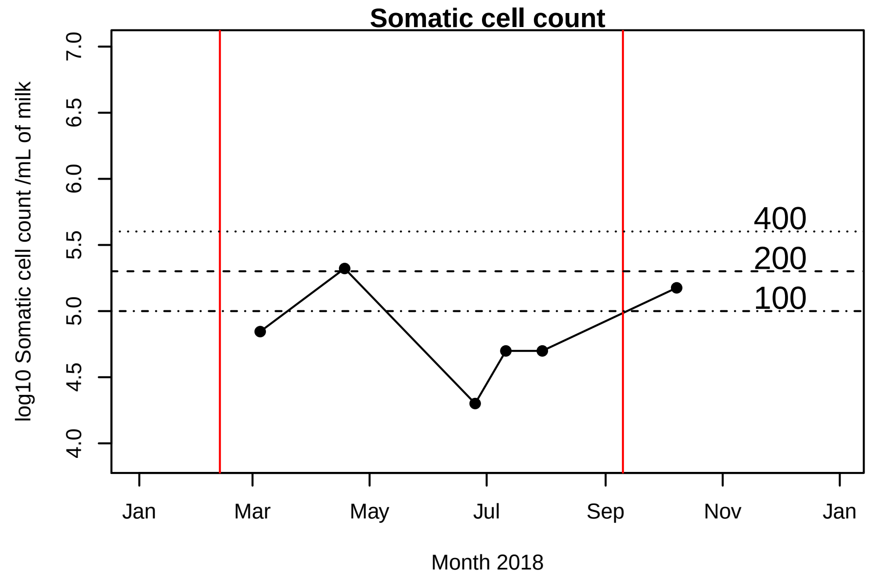

	# quarter	Sampling 1	Sampling 2
**Aerococcus viridans**	5	3	3
**Bacillus pumilus**	1	1	0
**Staphylococcus epidermidis**	2	2	0
**Staphylococcus haemolyticus**	1	1	0
**Staphylococcus hominis**	1	0	1

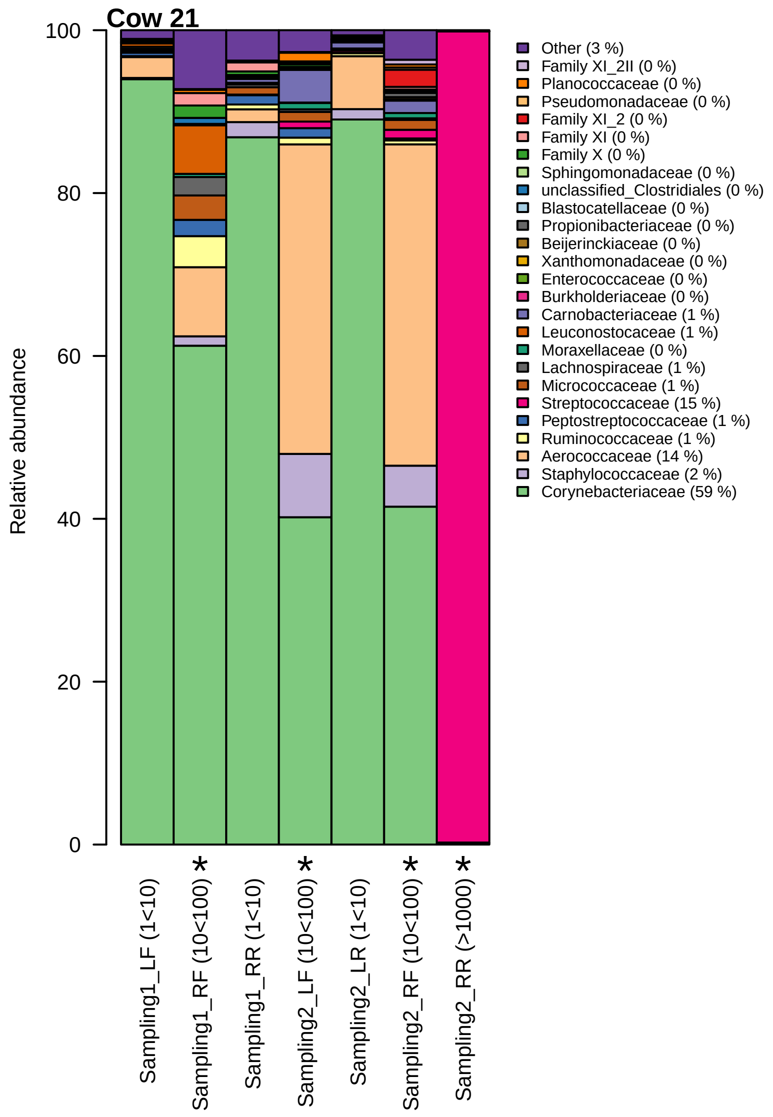

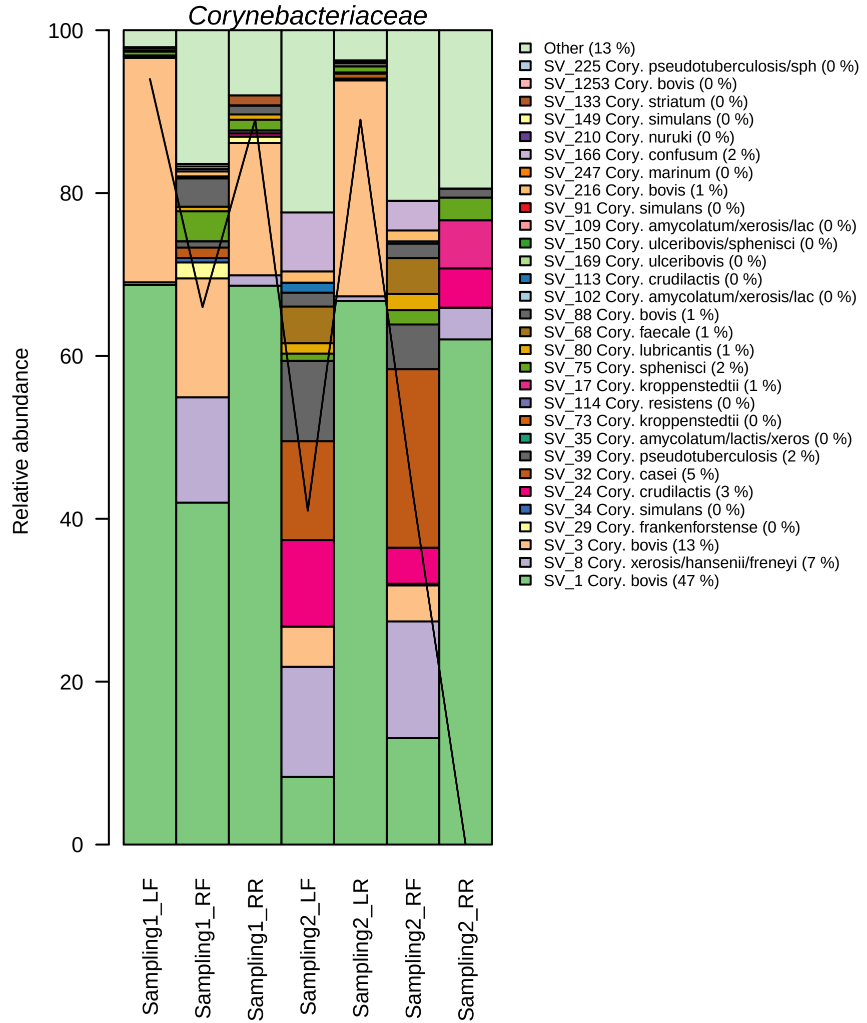

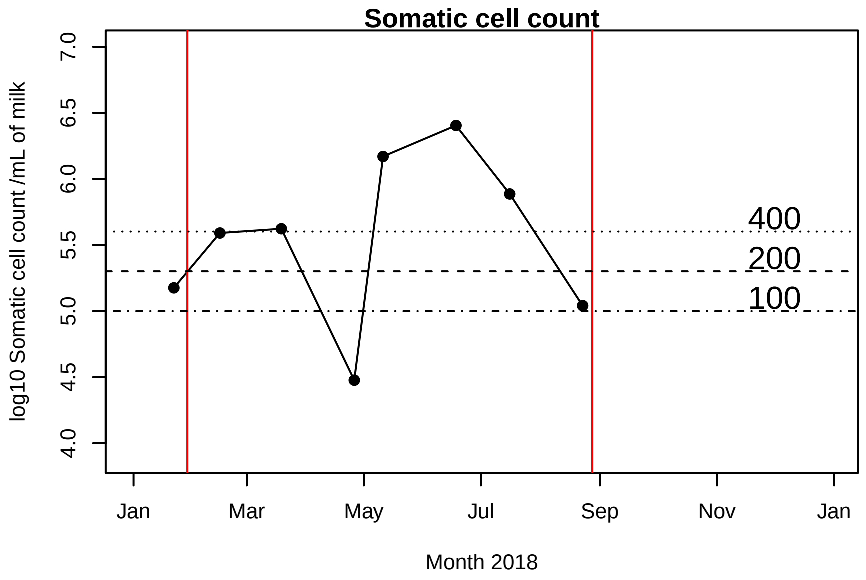

	# quarter	Sampling 1	Sampling 2
**Aerococcus viridans**	6	7	9
**Corynebacterium confusum**	1	0	1
**Corynebacterium stationis**	1	0	1
**Enterococcus faecium**	1	0	1
**Pediococcus pentosaceus**	1	1	0
**Staphylococcus aureus**	1	0	1
**Staphylococcus epidermidis**	1	1	0
**Staphylococcus hominis**	2	0	3
**Staphylococcus saprophyticus**	2	1	1
**Streptococcus canis**	2	1	1
**Streptococcus dysgalactiae**	2	3	4
**Streptococcus oralis**	1	1	0

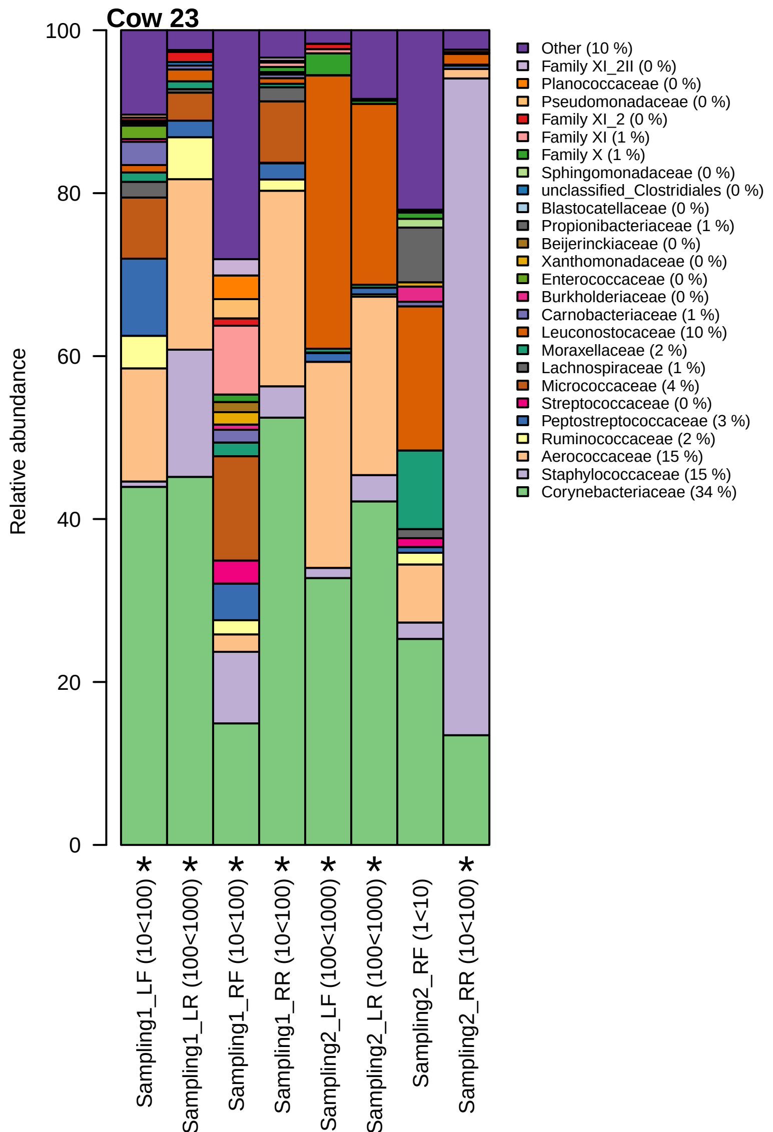

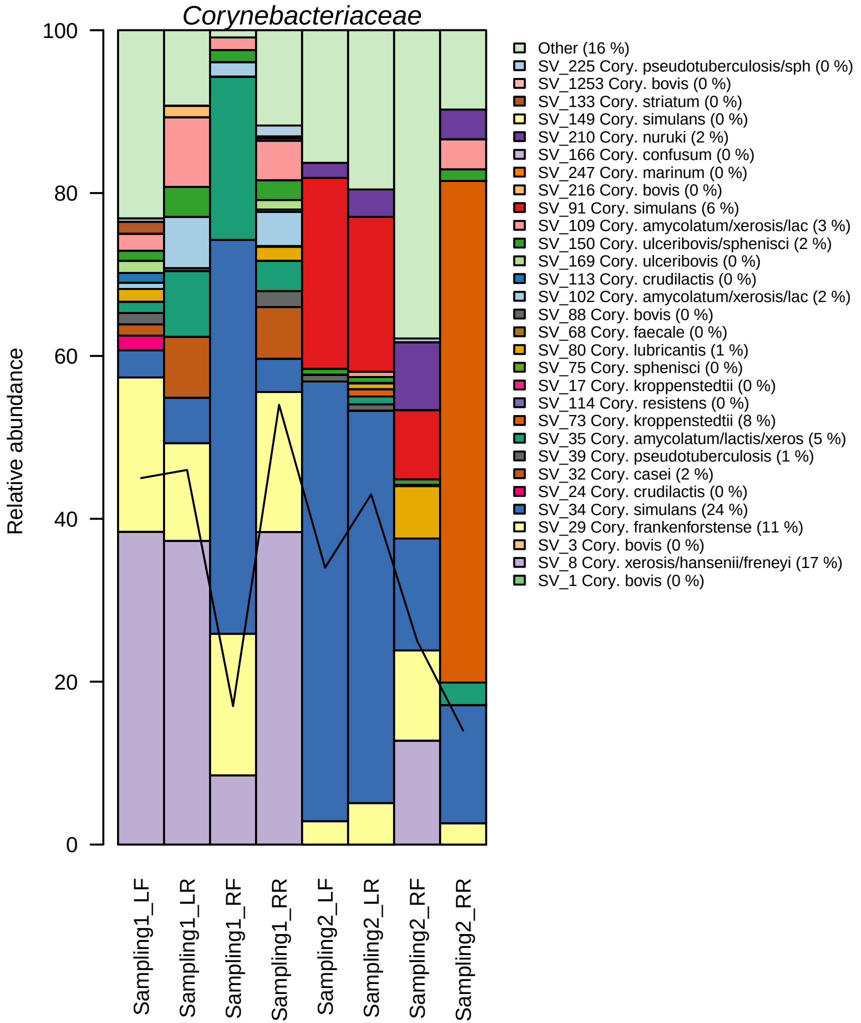

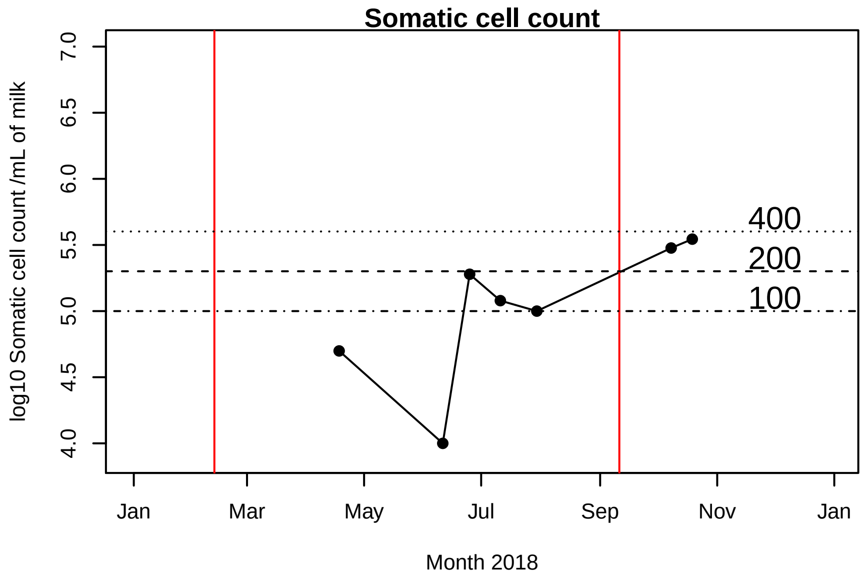

	# quarter	Sampling 1	Sampling 2
**Aerococcus viridans**	8	6	8
**Bacillus cereus**	2	0	2
**Bacillus clausii**	1	0	1
**Bacillus licheniformis**	2	0	2
**Bacillus pumilus**	3	2	1
**Corynebacterium amycolatum**	2	2	0
**Corynebacterium sp**	1	1	0
**Enterococcus gallinarum**	1	0	1
**Pediococcus pentosaceus**	3	2	1
**Staphylococcus epidermidis**	6	4	5
**Staphylococcus haemolyticus**	2	0	2
**Staphylococcus hominis**	3	0	3

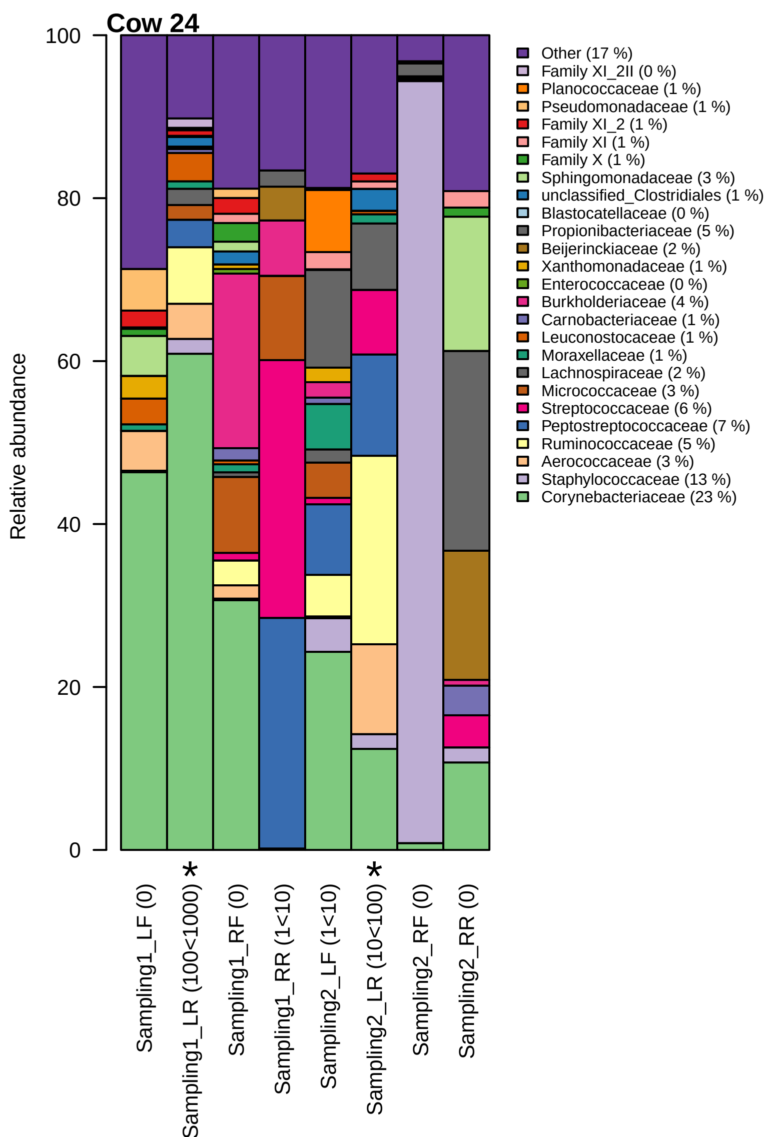

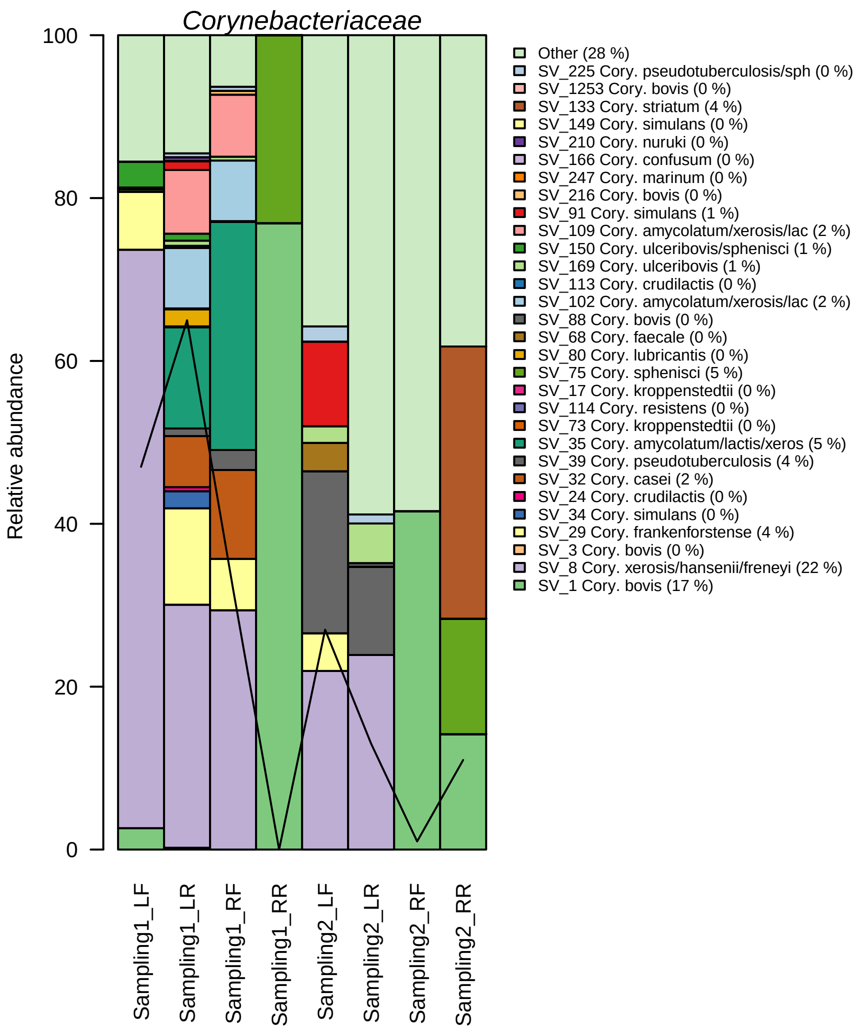

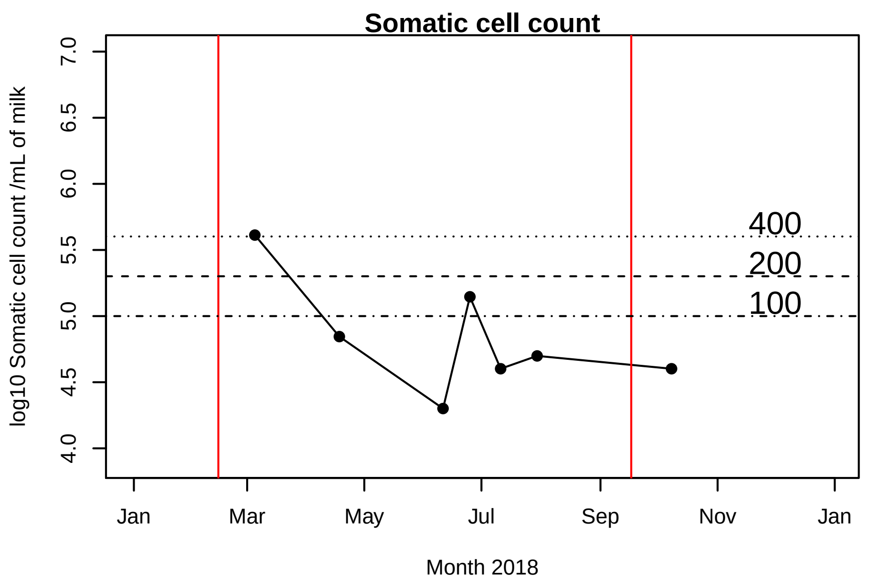

	# quarter	Sampling 1	Sampling 2
**Aerococcus viridans**	2	3	1
**Corynebacterium amycolatum**	2	3	0
**Escheria coli**	1	0	1
**Lactobacillus pentosus**	1	1	0
**Staphylococcus epidermidis**	2	3	2
**Staphylococcus saprophyticus**	1	1	0
**Streptococcus lutetiensis**	1	0	1
**Tetragenococcus solitarius**	1	1	0

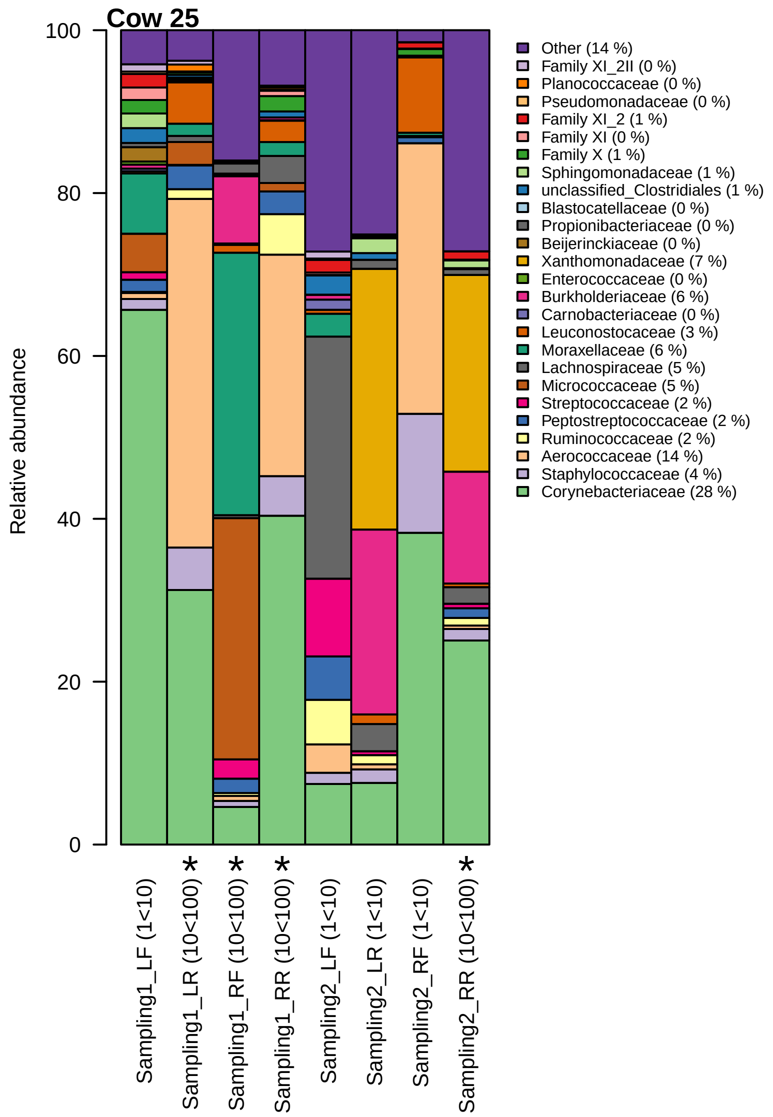

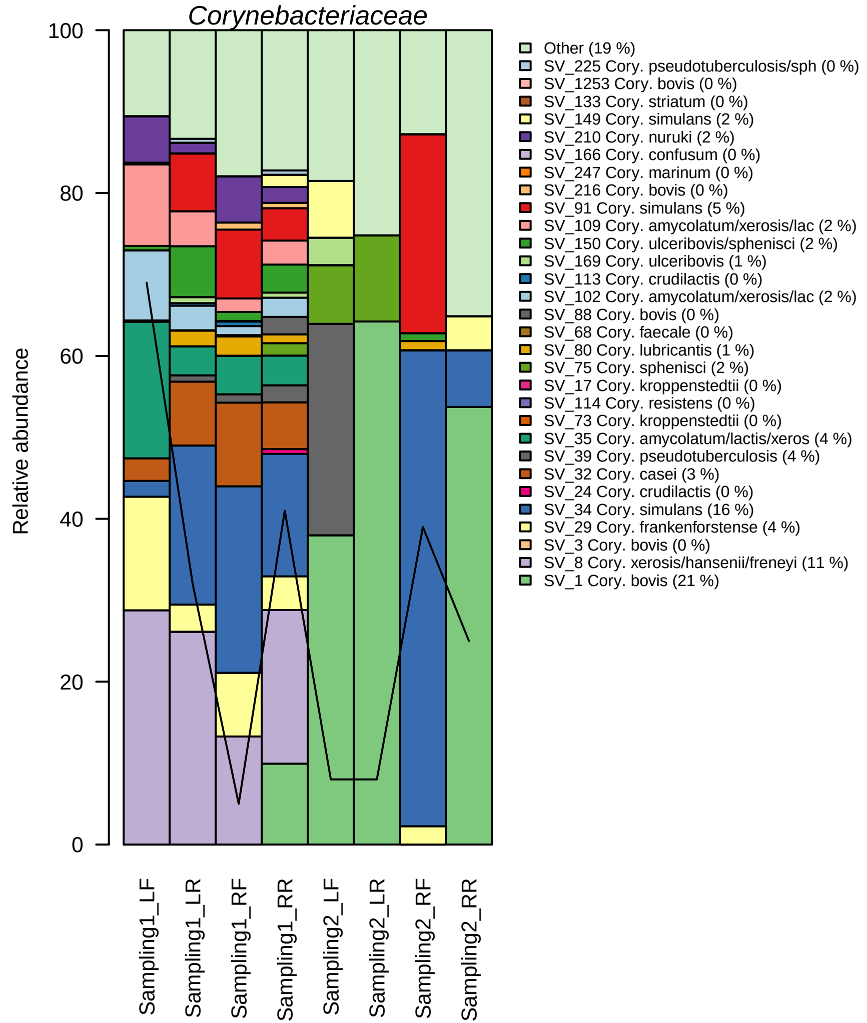

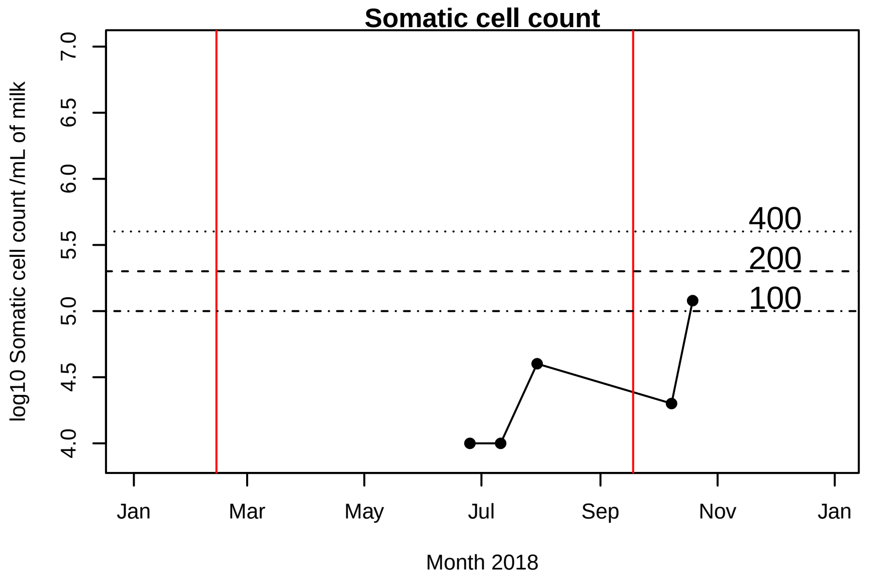

	# quarter	Sampling 1
**Acinetobacter guillouiae**	1	1
**Acinetobacter parvus**	1	1
**Aerococcus viridans**	2	5
**Bacillus licheniformis**	2	3
**Bacillus pumilus**	1	1
**Corynebacterium casei**	1	1
**Globicatella sulfidifaciens**	1	1
**Lactobacillus paracasei**	1	1
**Pediococcus pentosaceus**	1	1
**Staphylococcus capitis**	1	1
**Staphylococcus epidermidis**	2	4
**Staphylococcus hominis**	2	2
**Staphylococcus sciuri**	1	1
**Staphylococcus warneri**	1	1
**Streptococcus parauberis**	1	1

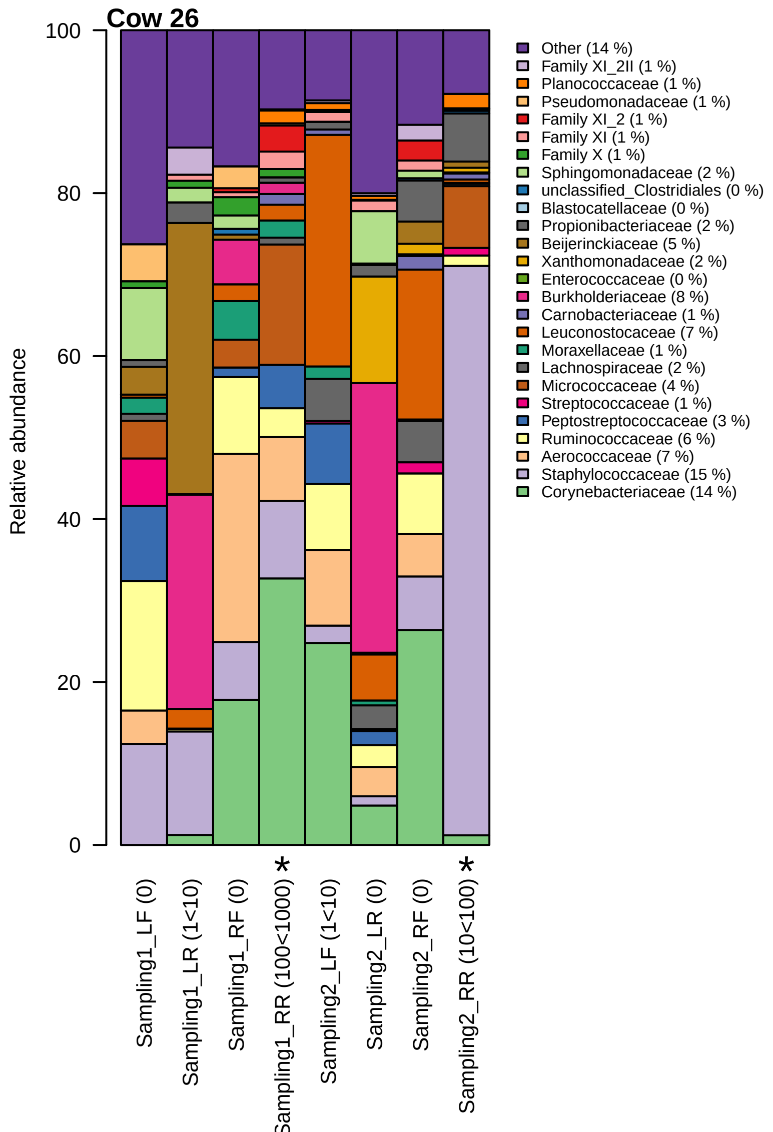

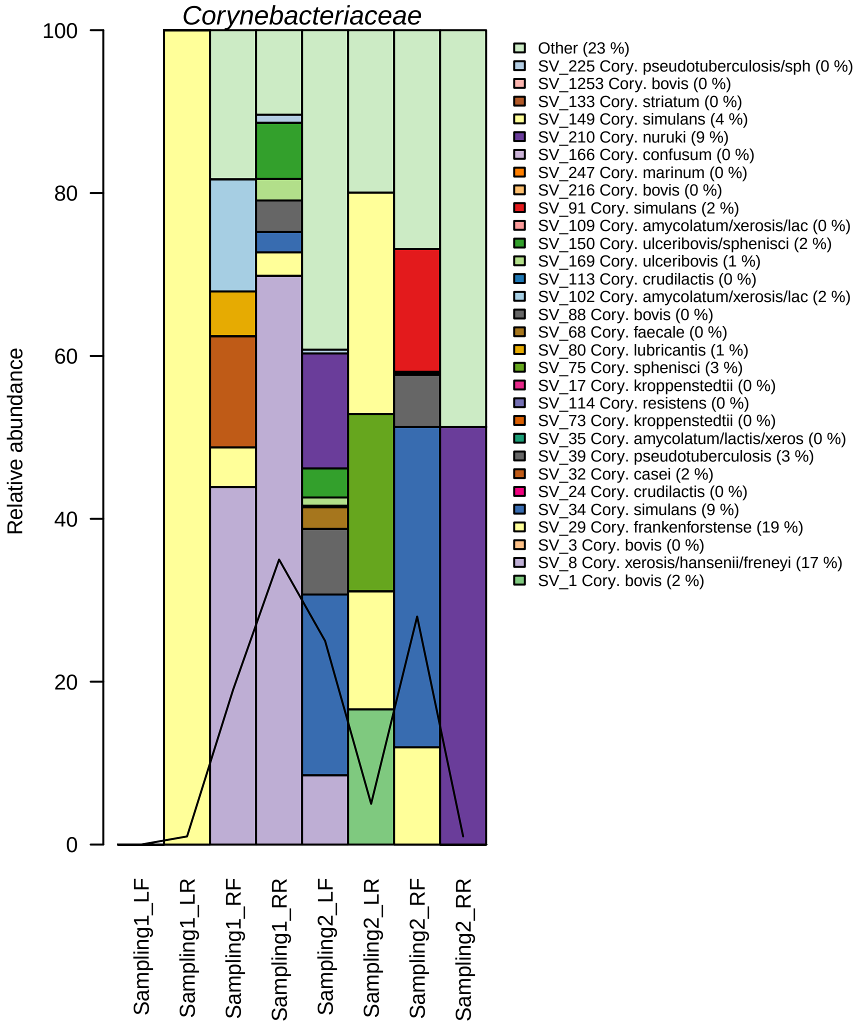

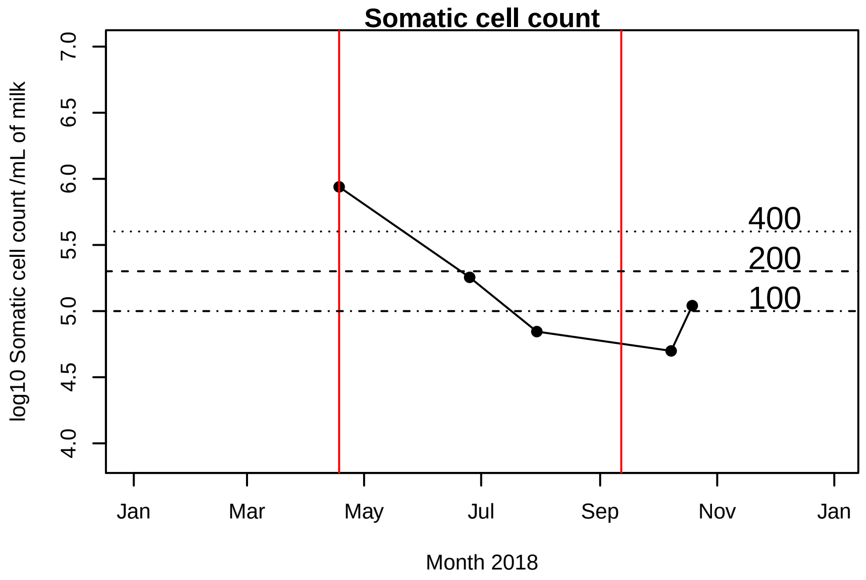

	# quarter	Sampling 1	Sampling 2
**Aerococcus viridans**	2	2	0
**Bacillus licheniformis**	1	2	0
**Bacillus pumilus**	1	2	0
**Pediococcus pentosaceus**	2	1	1
**Staphylococcus chromogenes**	2	2	4
**Staphylococcus hominis**	1	1	0
**Staphylococcus simulans**	1	1	0

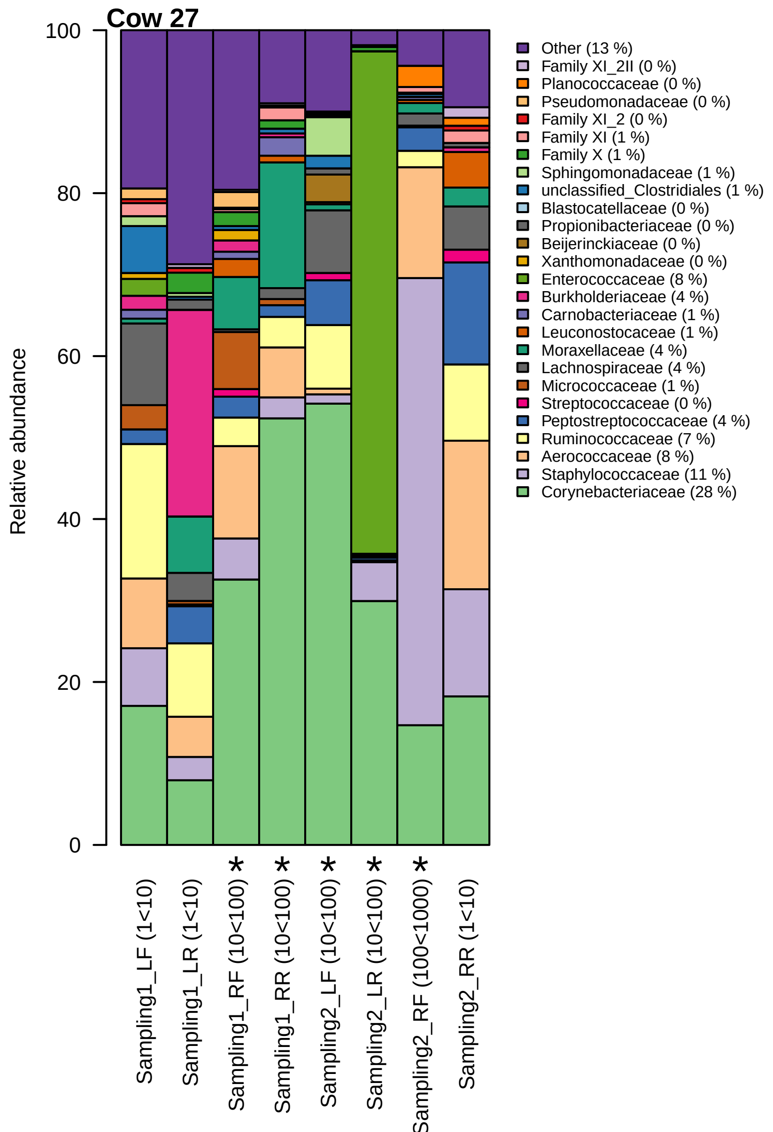

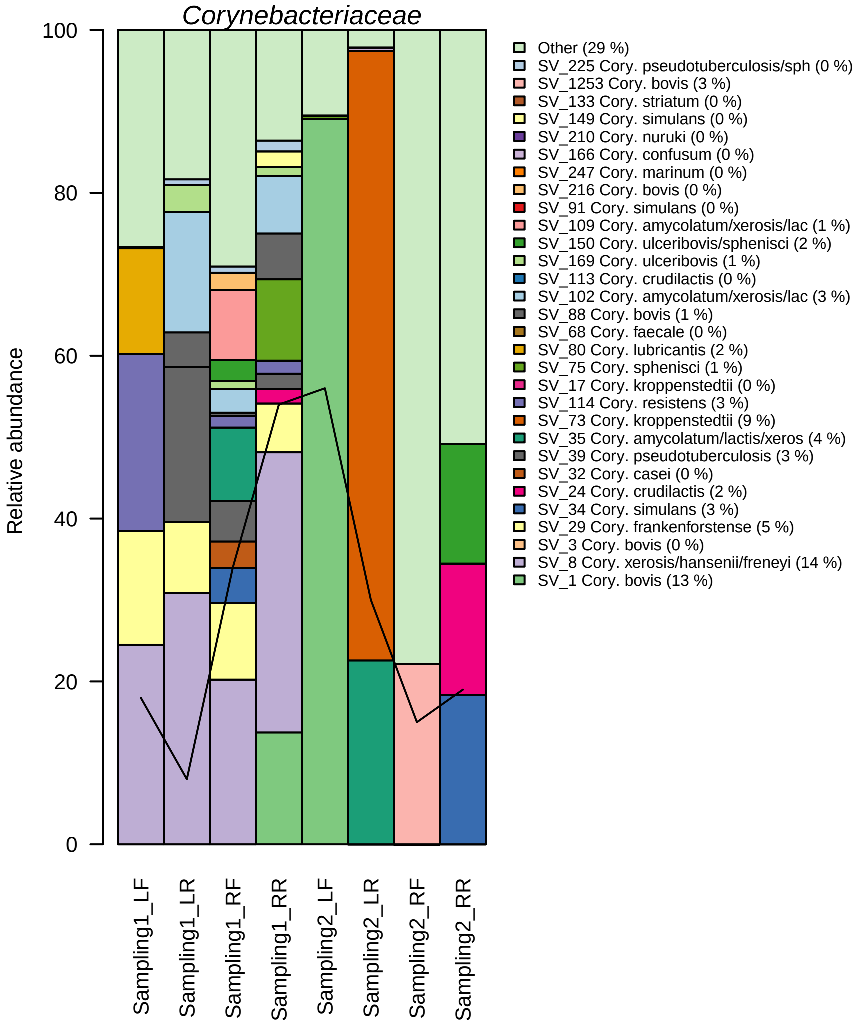

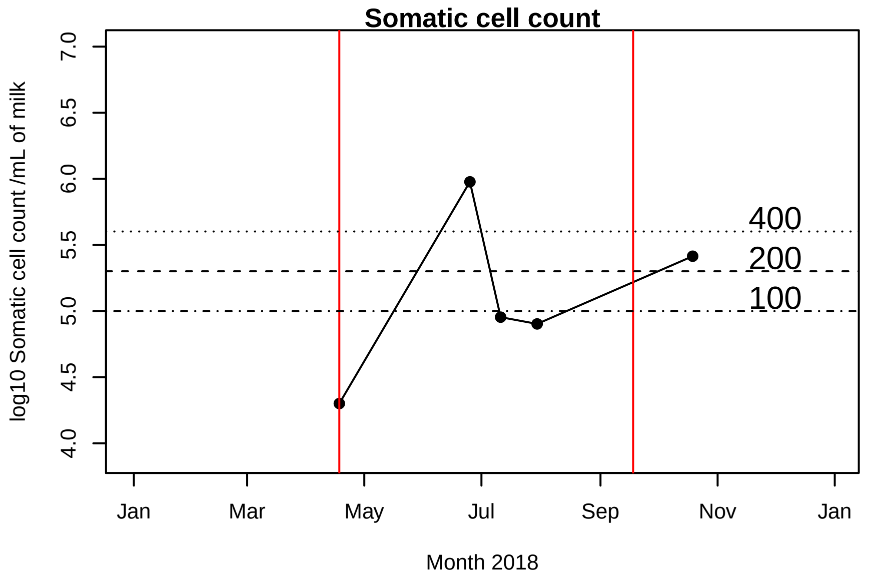

	# quarter	Sampling 1
**Aerococcus viridans**	2	2
**Bacillus licheniformis**	1	1
**Bacillus pumilus**	1	2
**Corynebacterium amycolatum**	1	1
**Enterococcus faecalis**	1	3
**Pediococcus pentosaceus**	1	1
**Staphylococcus epidermidis**	3	4
**Staphylococcus warneri**	1	1
**Streptococcus pluranimalium**	1	1

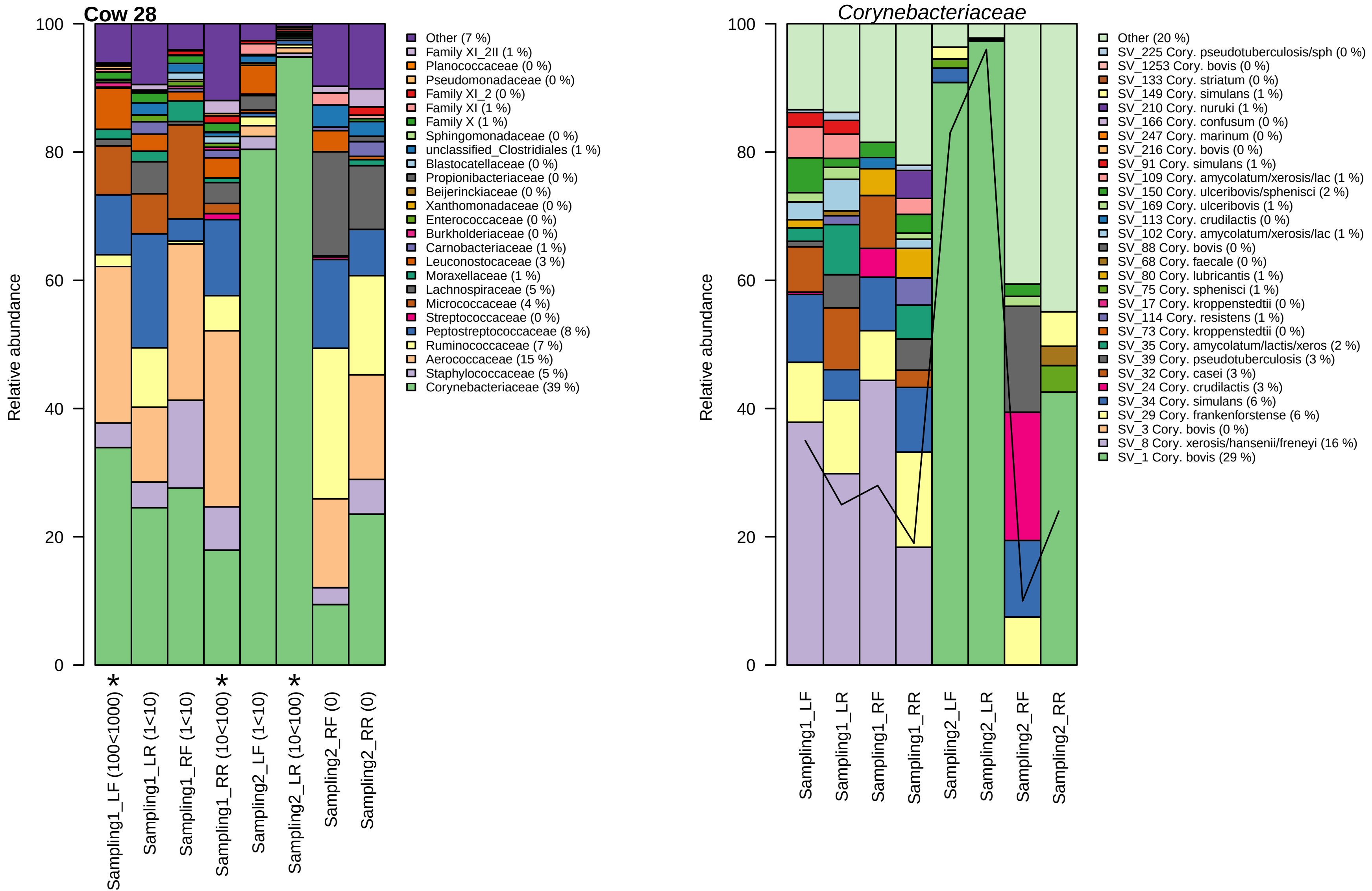

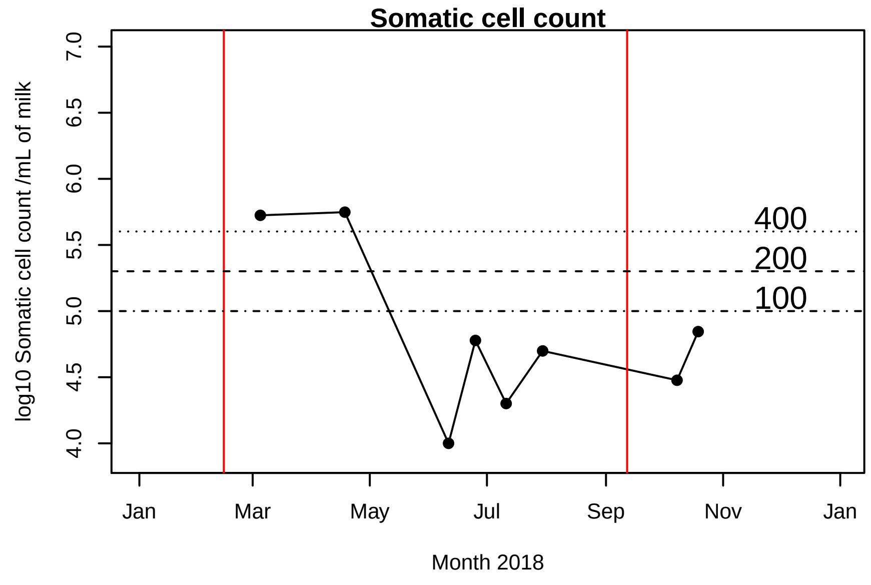

	# quarter	Sampling 1	Sampling 2
**Aerococcus viridans**	3	4	1
**Bacillus licheniformis**	1	1	0
**Bacillus pumilus**	2	2	0
**Corynebacterium amycolatum**	1	1	0
**Corynebacterium bovis**	1	0	1
**Corynebacterium sp**	1	0	1
**Pediococcus pentosaceus**	2	3	0
**Staphylococcus chromogenes**	1	1	0
**Staphylococcus epidermidis**	3	6	0
**Staphylococcus haemolyticus**	1	1	0
**Staphylococcus hominis**	1	0	1
**Tetragenococcus solitarius**	2	2	0

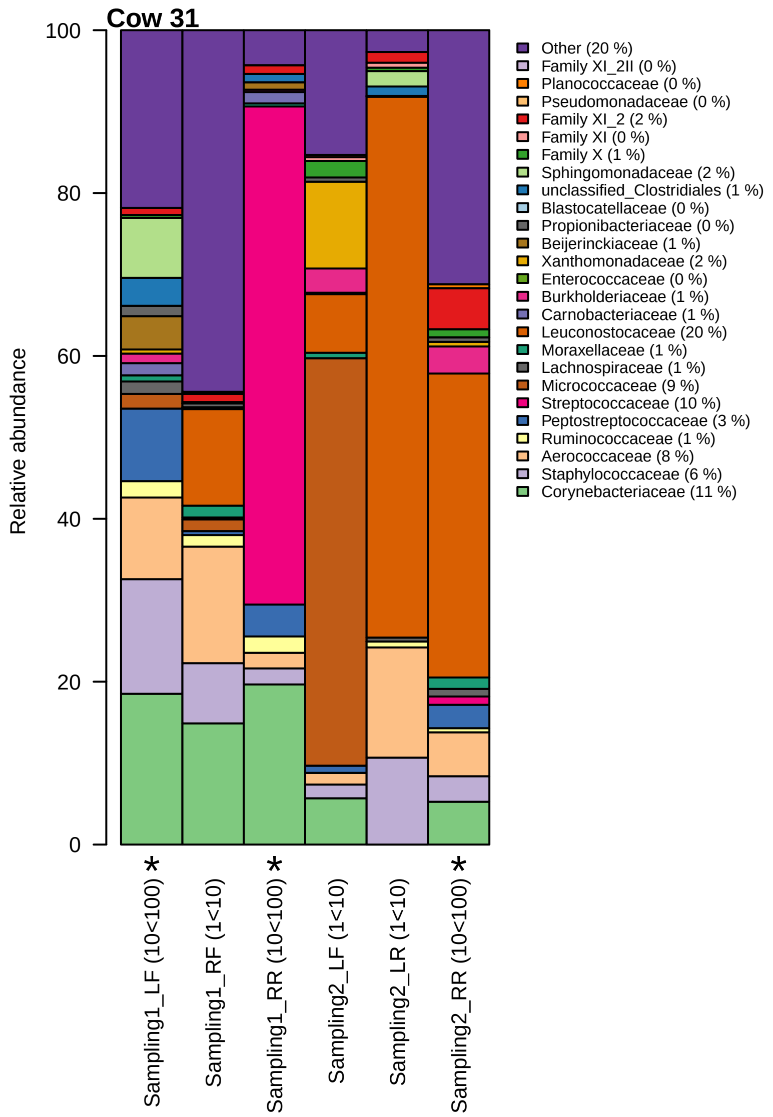

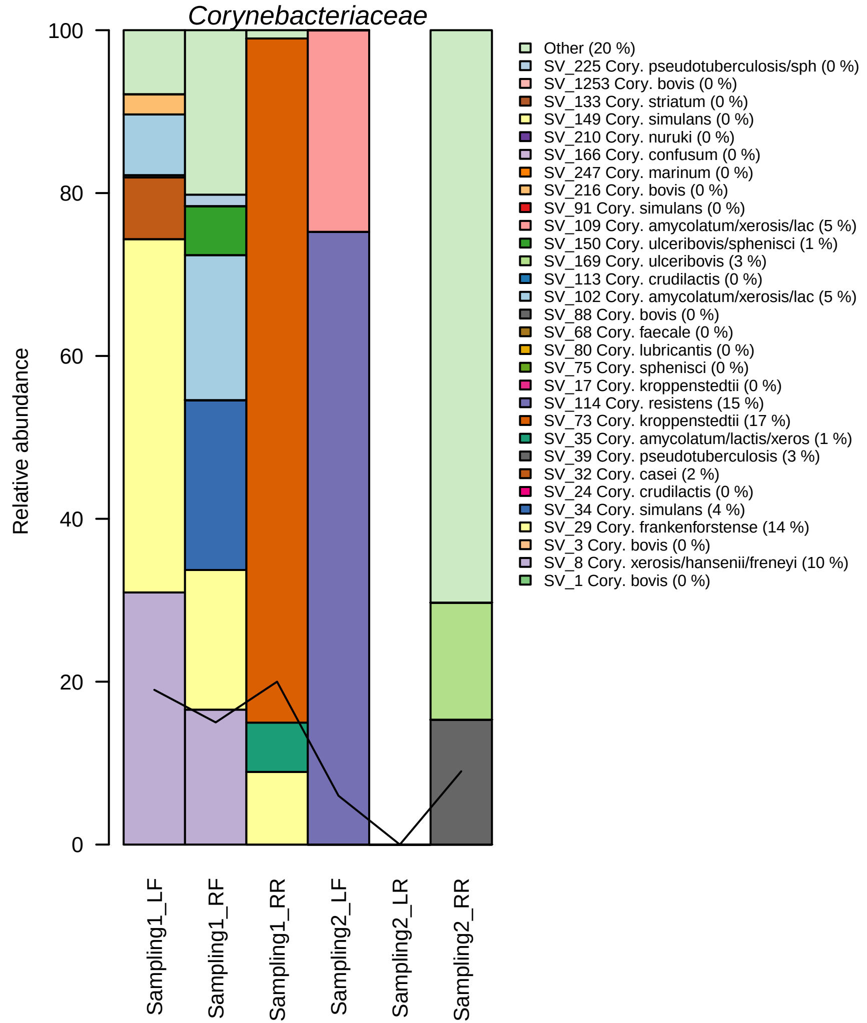

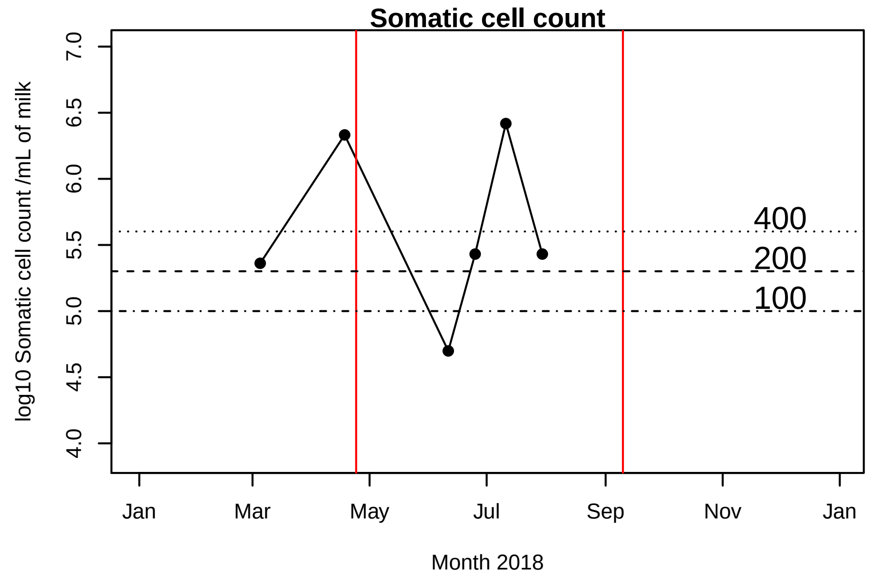

	# quarter	Sampling 1	Sampling 2
**Aerococcus viridans**	4	3	4
**Corynebacterium amycolatum**	1	0	1
**Staphylococcus capitis**	1	3	0
**Staphylococcus epidermidis**	1	3	0
**Streptococcus canis**	1	4	0
**Streptococcus dysgalactiae**	1	2	0

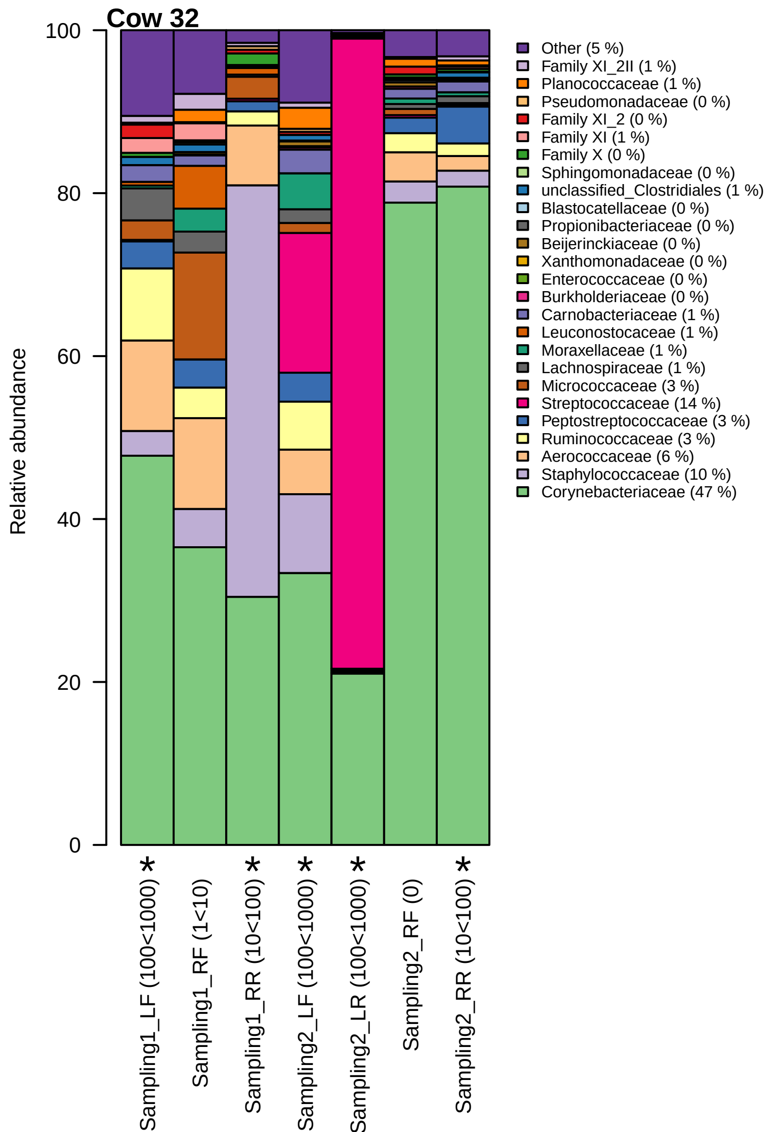

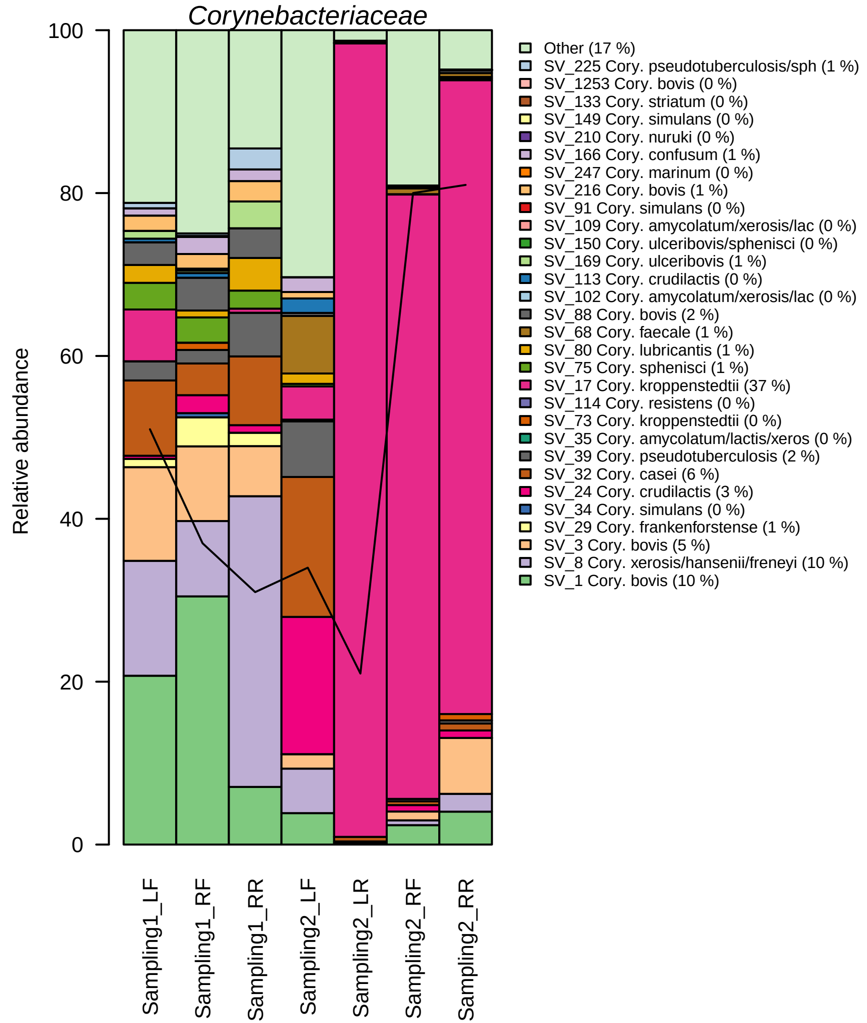

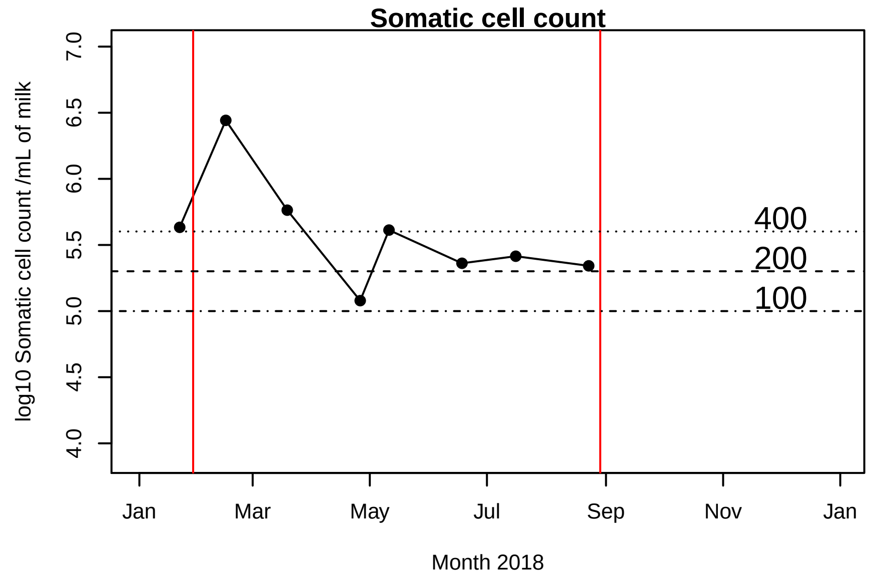

	# quarter	Sampling 1	Sampling 2
**Aerococcus viridans**	6	12	6
**Corynebacterium confusum**	1	0	1
**Corynebacterium sp**	1	0	1
**Pediococcus pentosaceus**	1	1	0
**Staphylococcus aureus**	1	0	1
**Staphylococcus chromogenes**	2	0	5
**Staphylococcus epidermidis**	2	4	0
**Staphylococcus haemolyticus**	1	0	1
**Staphylococcus hominis**	2	2	0
**Staphylococcus saprophyticus**	3	3	0
**Streptococcus dysgalactiae**	1	1	0
**Streptococcus uberis**	2	0	7

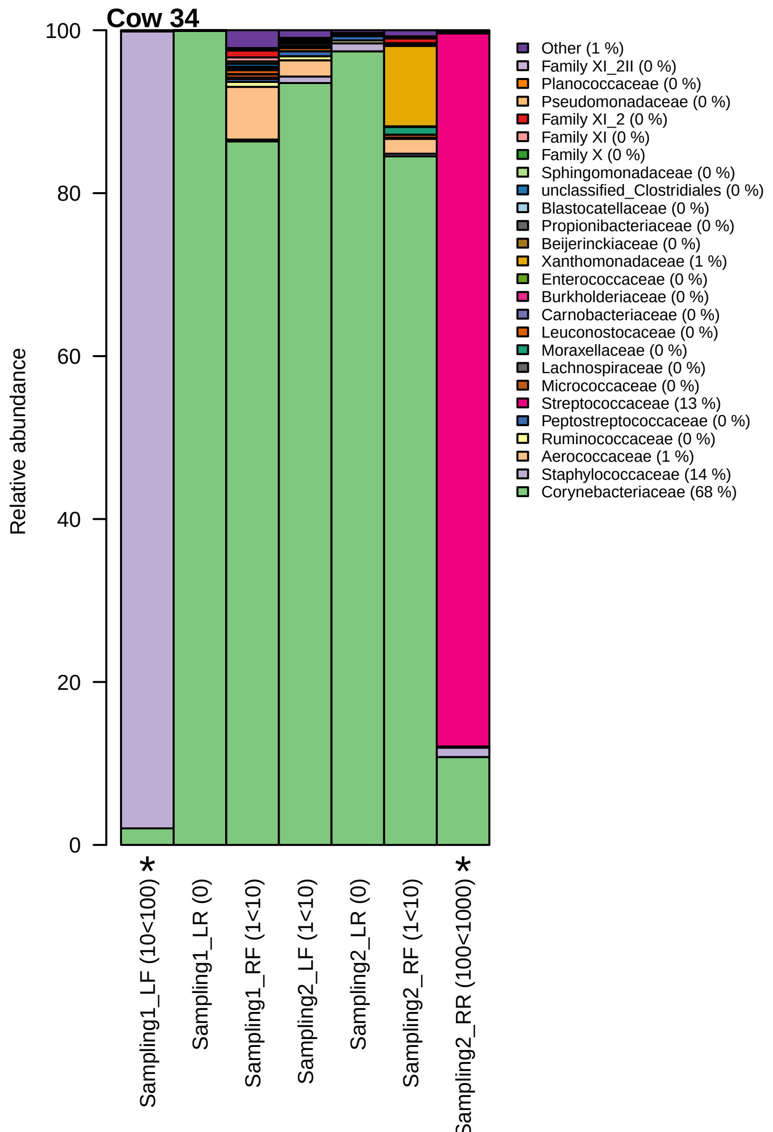

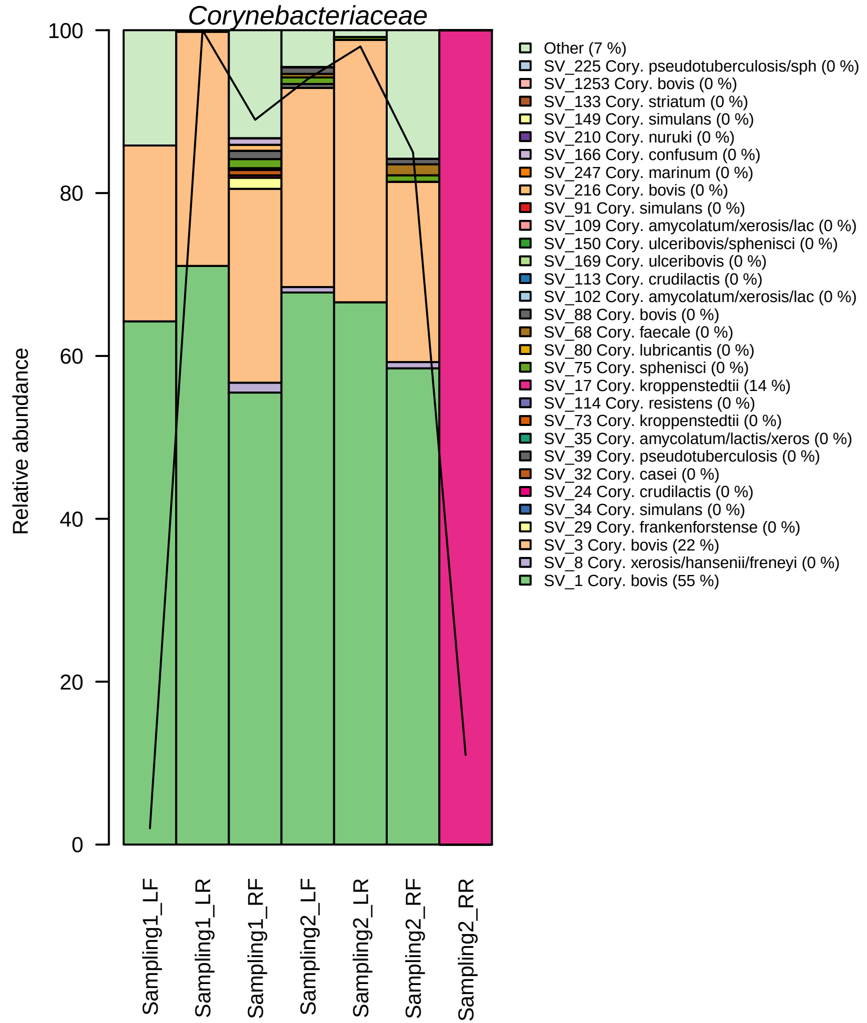

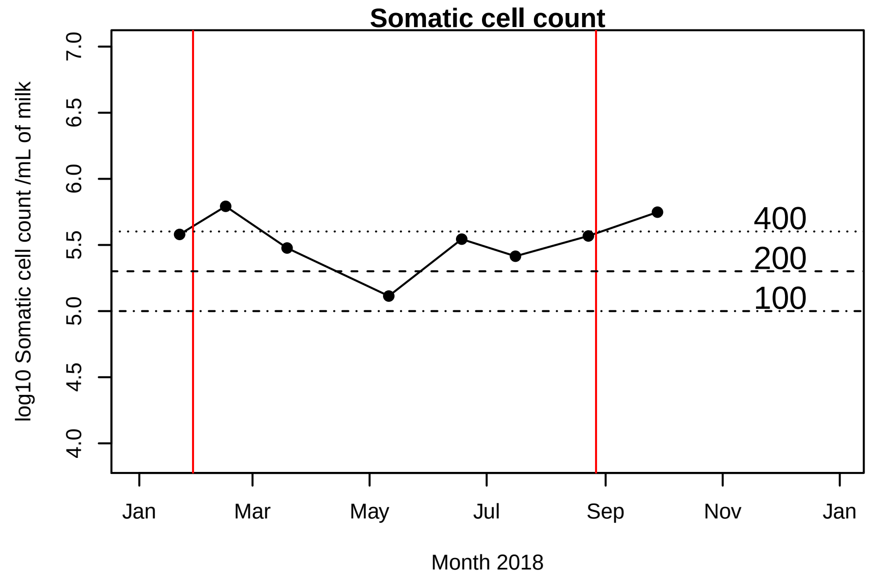

	# quarter	Sampling 1	Sampling 2
**Aerococcus viridans**	4	2	3
**Pantoea agglomerans**	1	0	1
**Staphylococcus epidermidis**	1	5	0
**Streptococcus uberis**	2	4	5

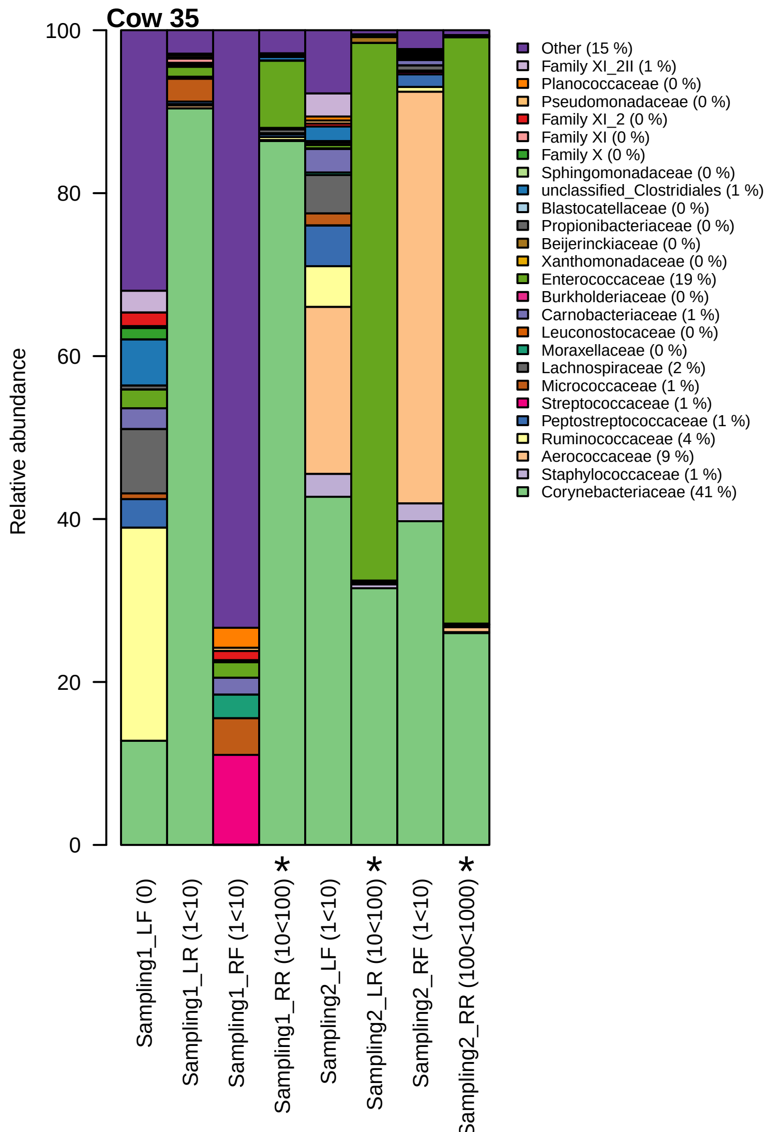

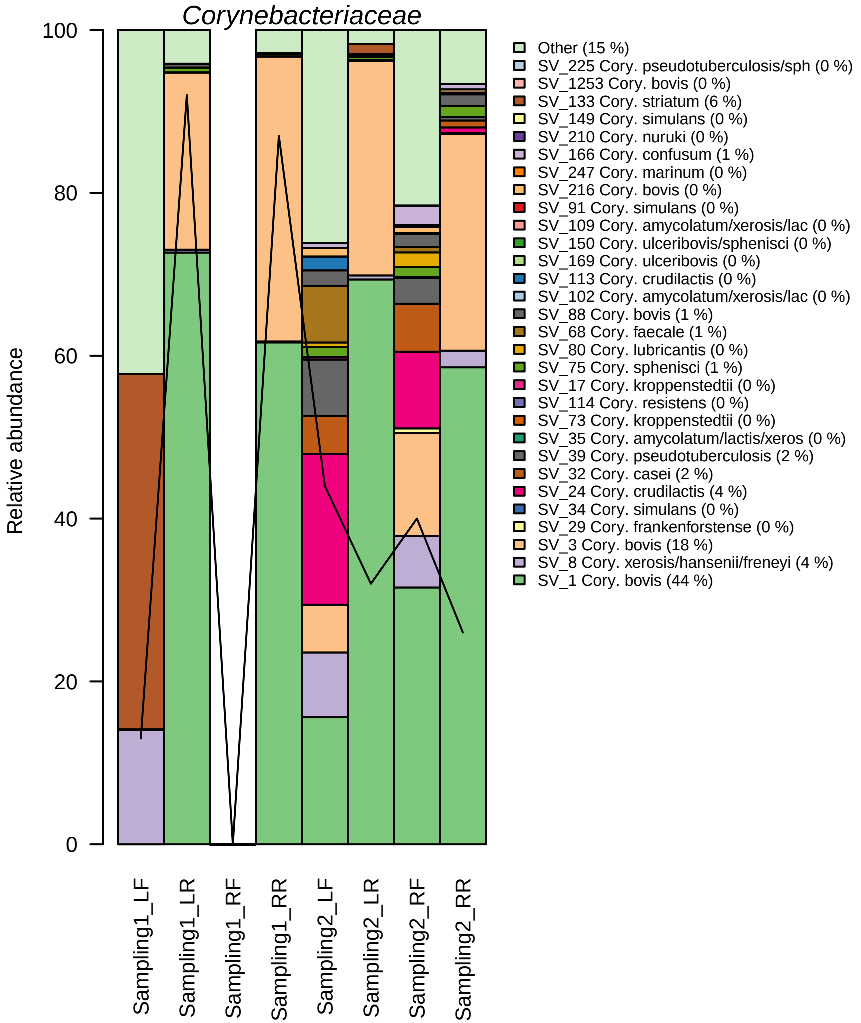

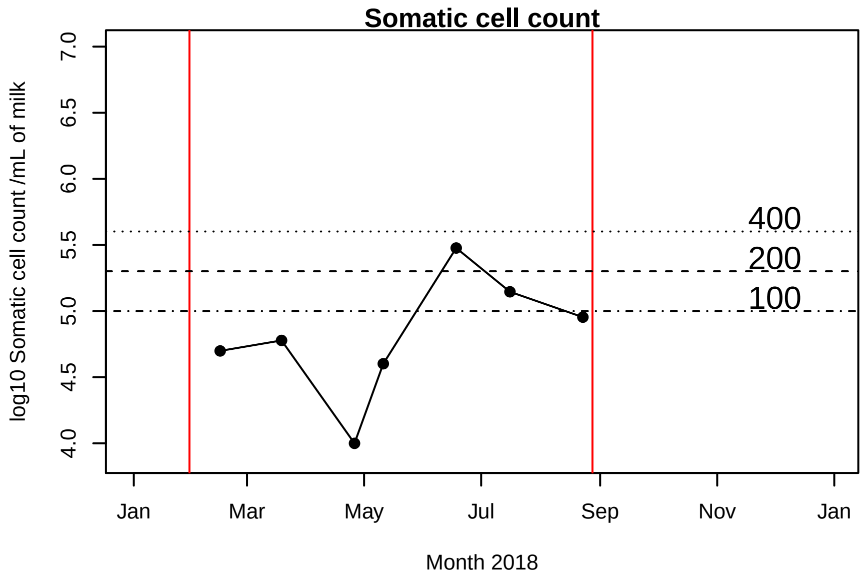

	# quarter	Sampling 1	Sampling 2
**Aerococcus viridans**	2	0	4
**Bacillus mycoides**	1	1	0
**Enterococcus faecalis**	4	15	8

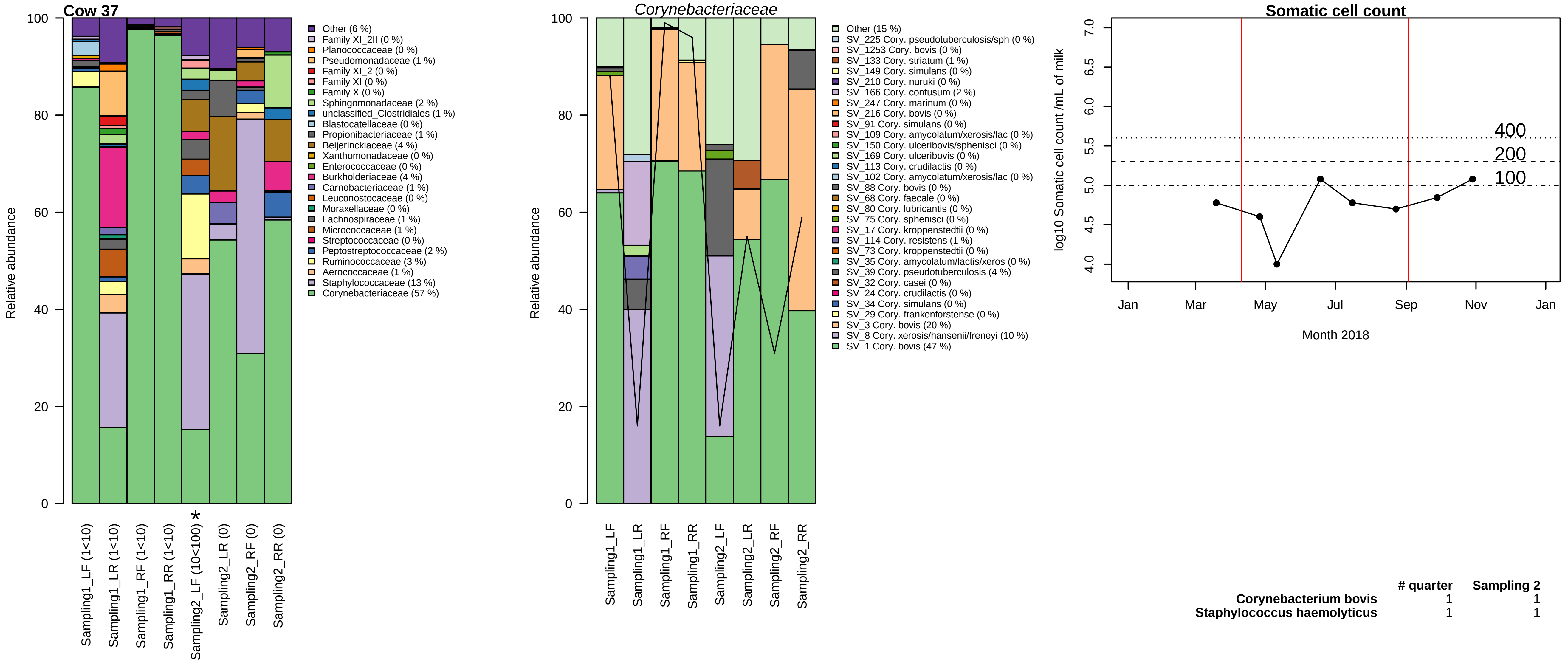
Cow 37
Relative abundance
Other (6 %)
Family XI_2II (0 %)
Planococcaceae (0 %)
Pseudomonadaceae (1 %)
Family XI_2 (0 %)
Family XI (0 %)
Family X (0 %)
Sphingomonadaceae (2 %)
unclassified_Clostridiales (1 %)
Blastocatellaceae (0 %)
Propionibacteriaceae (1 %)
Beijerinckiaceae (4 %)
Xanthomonadaceae (0 %)
Enterococcaceae (0 %)
Burkholderiaceae (4 %)
Carnobacteriaceae (1 %)
Leuconostocaceae (0 %)
Moraxellaceae (0 %)
Lachnospiraceae (1 %)
Micrococcaceae (1 %)
Streptococcaceae (0 %)
Peptostreptococcaceae (2 %)
Ruminococcaceae (3 %)
Aerococcaceae (1 %)
Staphylococcaceae (13 %)
Corynebacteriaceae (57 %)
Sampling1_LF (1<10)
Sampling1_LR (1<10)
Sampling1_RF (1<10)
Sampling1_RR (1<10)
Sampling2_LF (10<100)
Sampling2_LR (0)
Sampling2_RF (0)
Sampling2_RR (0)
*
Corynebacteriaceae
Other (15 %)
SV_225 Cory. pseudotuberculosis/sph (0 %)
SV_1253 Cory. bovis (0 %)
SV_133 Cory. striatum (1 %)
SV_149 Cory. simulans (0 %)
SV_210 Cory. nuruki (0 %)
SV_166 Cory. confusum (2 %)
SV_247 Cory. marinum (0 %)
SV_216 Cory. bovis (0 %)
SV_91 Cory. simulans (0 %)
SV_109 Cory. amycolatum/xerosis/lac (0 %)
SV_150 Cory. ulceribovis/sphenisci (0 %)
SV_169 Cory. ulceribovis (0 %)
SV_113 Cory. crudilactis (0 %)
SV_102 Cory. amycolatum/xerosis/lac (0 %)
SV_88 Cory. bovis (0 %)
SV_68 Cory. faecale (0 %)
SV_80 Cory. lubricantis (0 %)
SV_75 Cory. sphenisci (0 %)
SV_17 Cory. kroppenstedtii (0 %)
SV_114 Cory. resistens (1 %)
SV_73 Cory. kroppenstedtii (0 %)
SV_35 Cory. amycolatum/lactis/xeros (0 %)
SV_39 Cory. pseudotuberculosis (4 %)
SV_32 Cory. casei (0 %)
SV_24 Cory. crudilactis (0 %)
SV_34 Cory. simulans (0 %)
SV_29 Cory. frankenforstense (0 %)
SV_3 Cory. bovis (20 %)
SV_8 Cory. xerosis/hansenii/freneyi (10 %)
SV_1 Cory. bovis (47 %)
Sampling1_LF
Sampling1_LR
Sampling1_RF
Sampling1_RR
Sampling2_LF
Sampling2_LR
Sampling2_RF
Sampling2_RR
Somatic cell count
log10 Somatic cell count /mL of milk
400
200
100
Jan
Mar
May
Jul
Sep
Nov
Jan
Month 2018
quarter
Sampling 2
Corynebacterium bovis 1 1
Staphylococcus haemolyticus 1 1

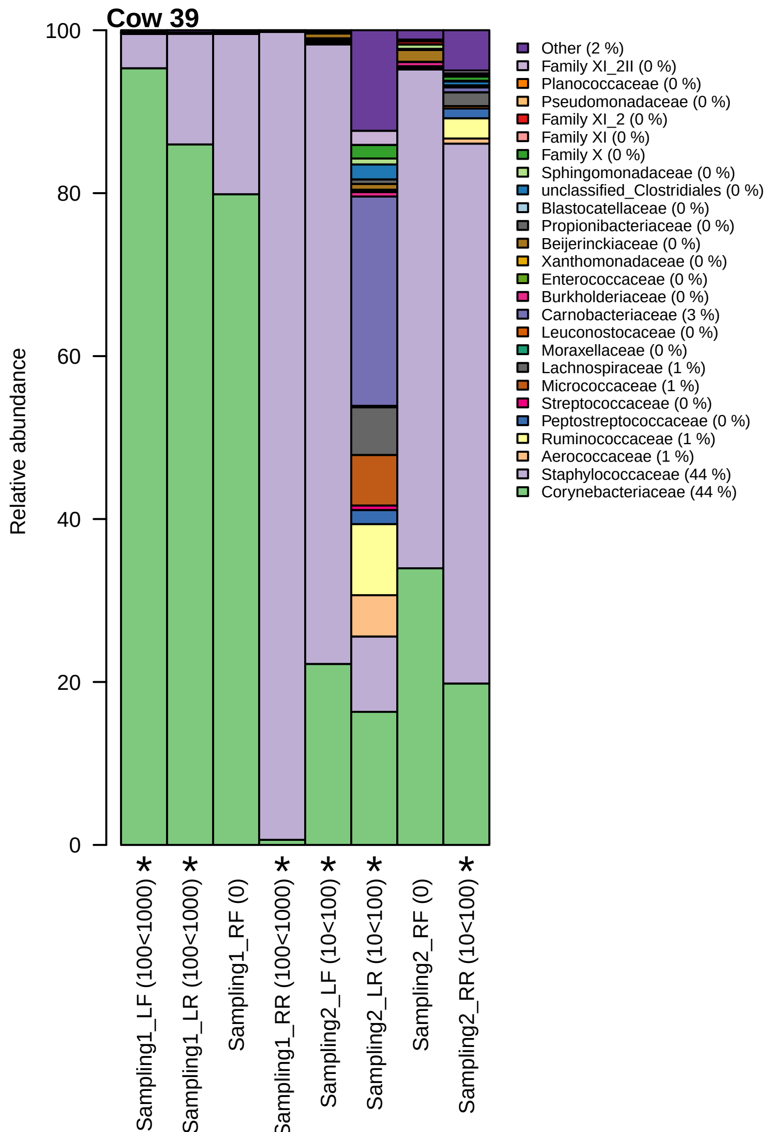

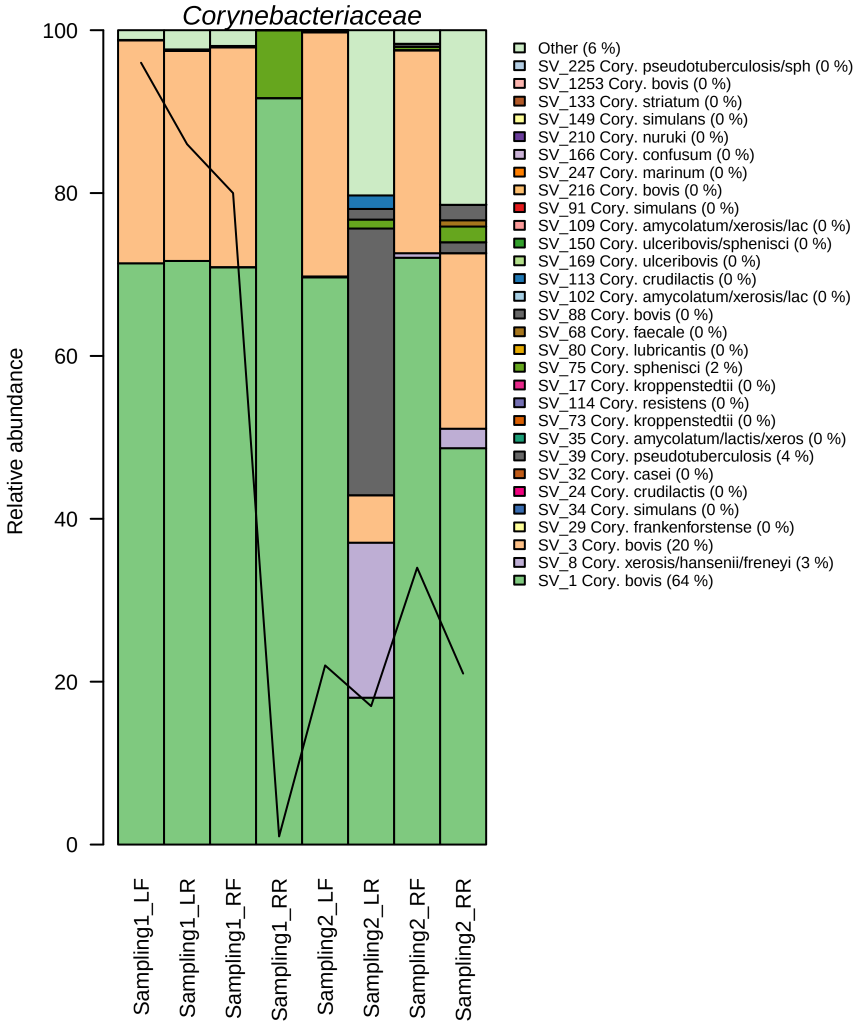

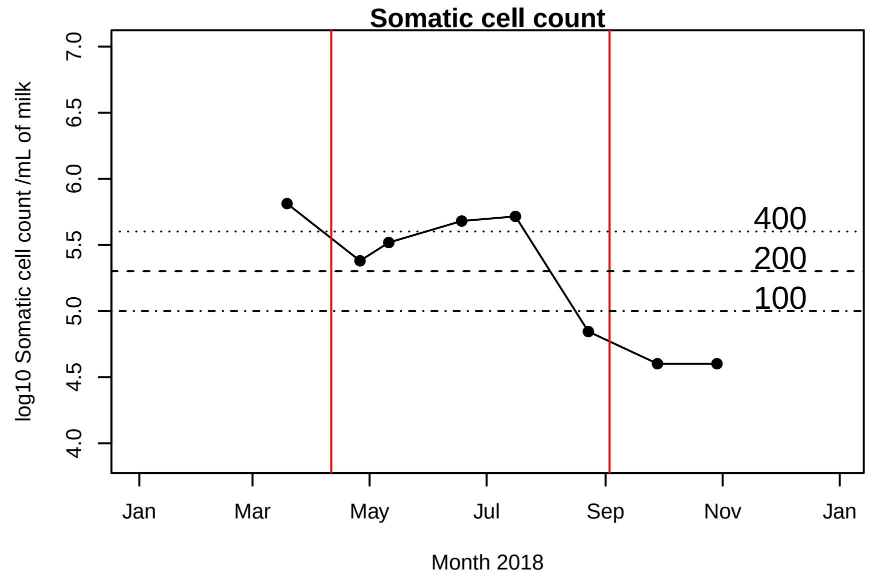

	# quarter	Sampling 1	Sampling 2
**Aerococcus viridans**	1	0	1
**Corynebacterium bovis**	1	0	1
**Staphylococcus epidermidis**	3	7	1
**Staphylococcus haemolyticus**	2	1	2

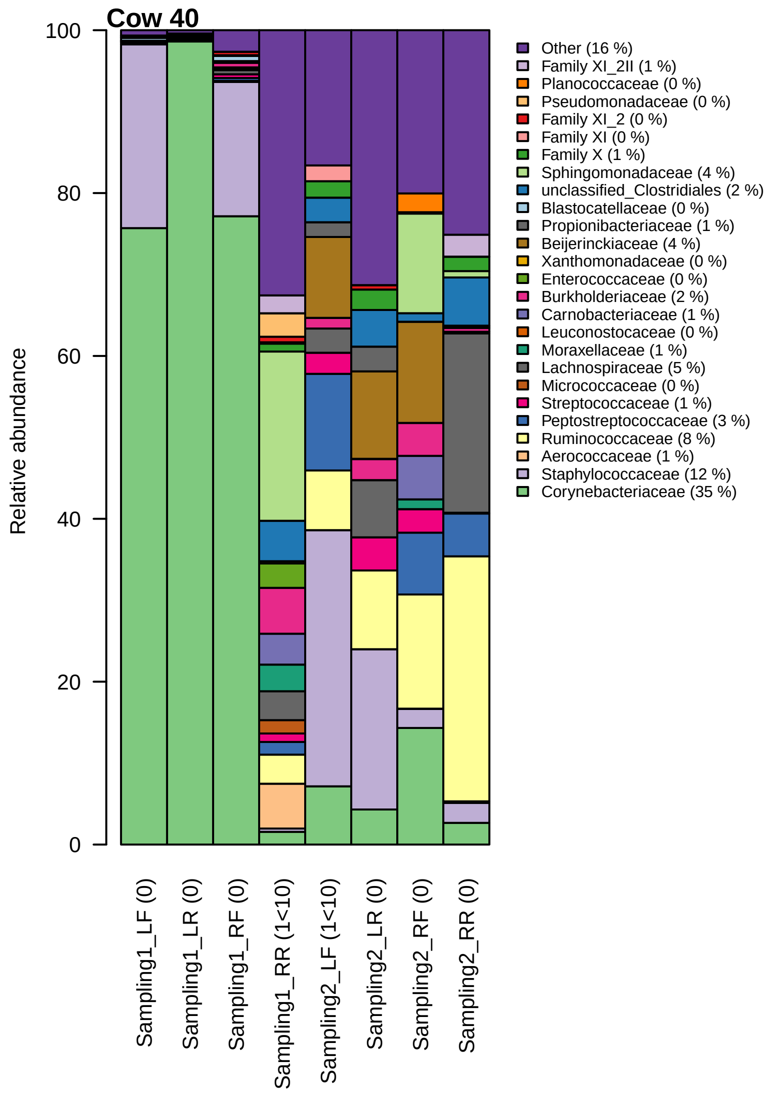

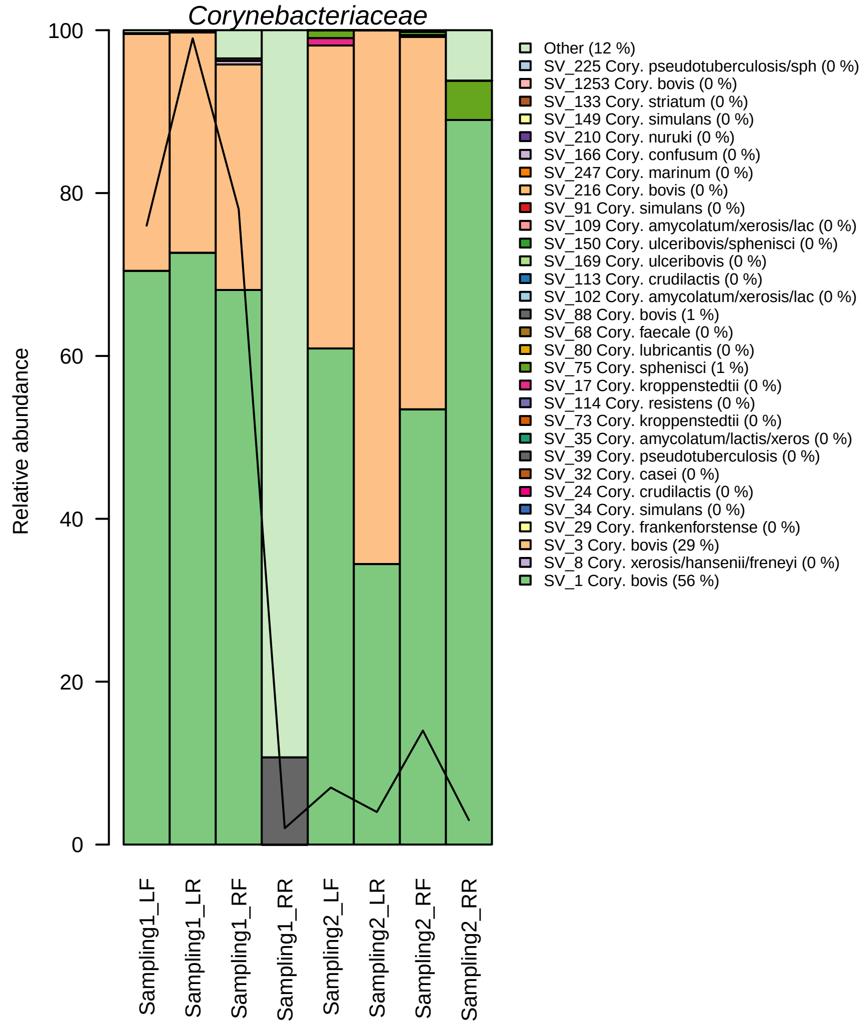

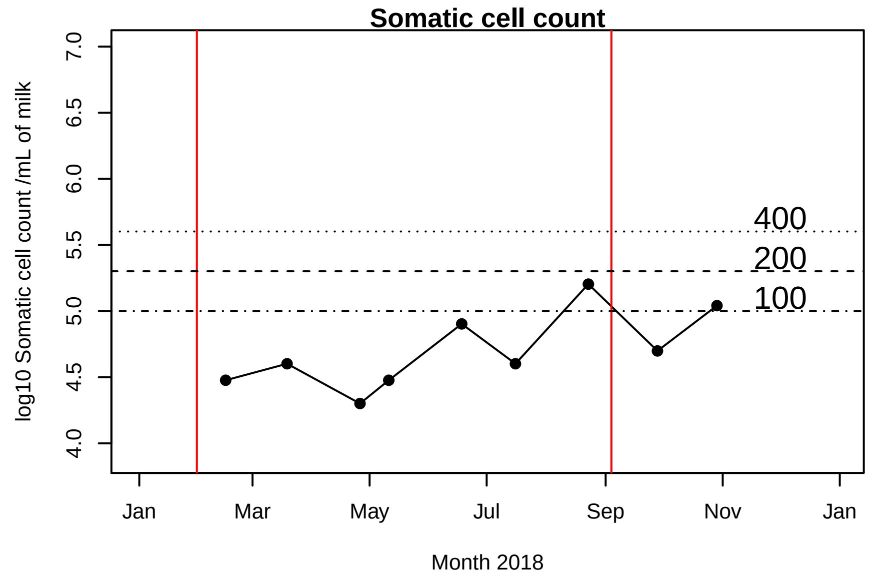

	# quarter	Sampling 1	Sampling 2
**Aerococcus viridans**	1	1	0
**Staphylococcus haemolyticus**	1	0	2

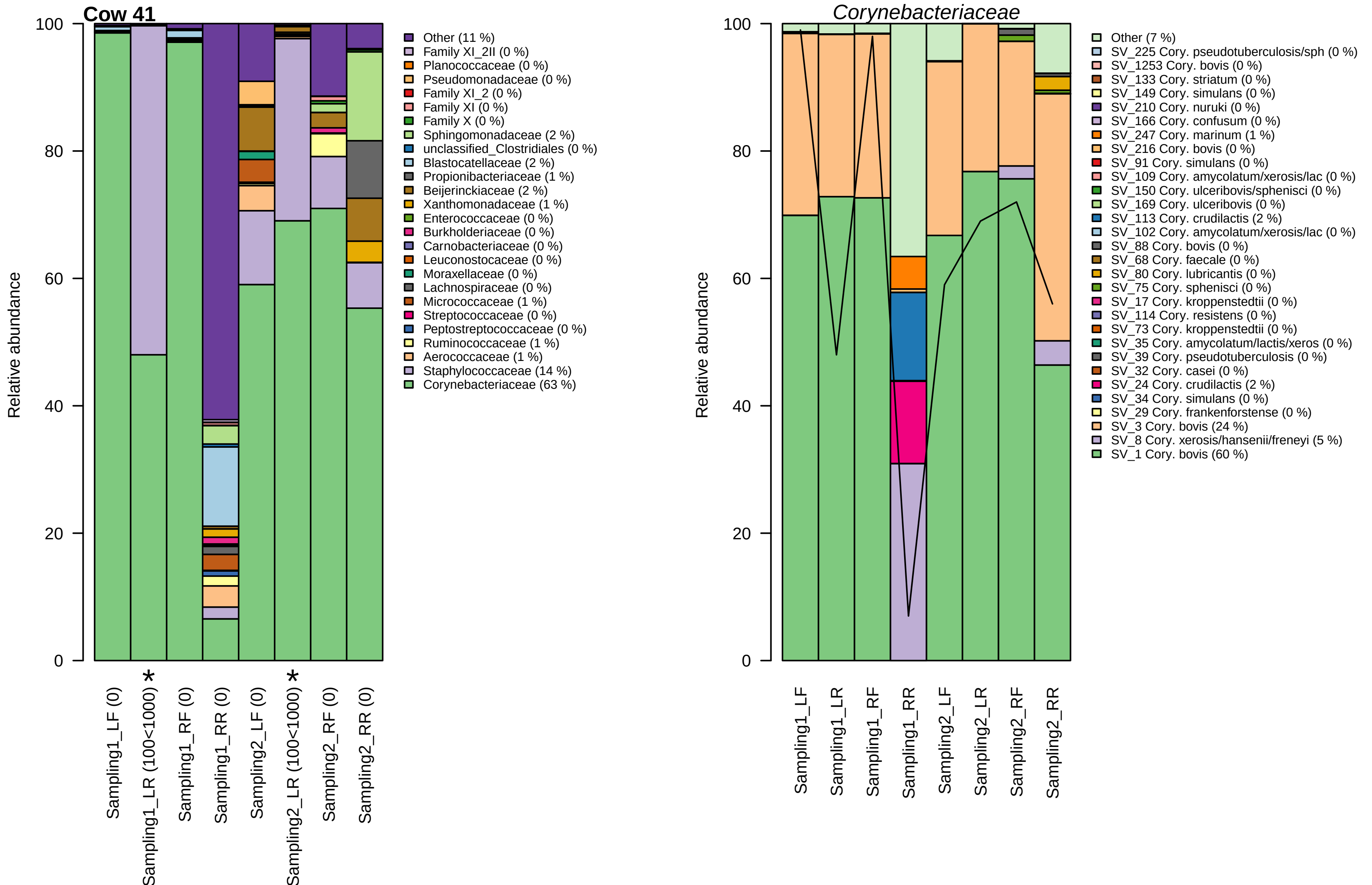

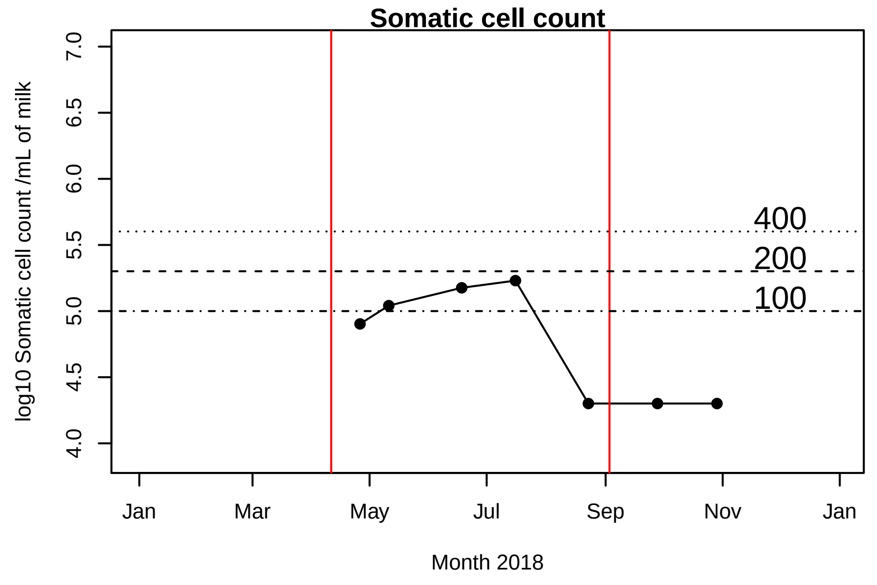

	# quarter	Sampling 1	Sampling 2
**Staphylococcus epidermidis**	2	7	4

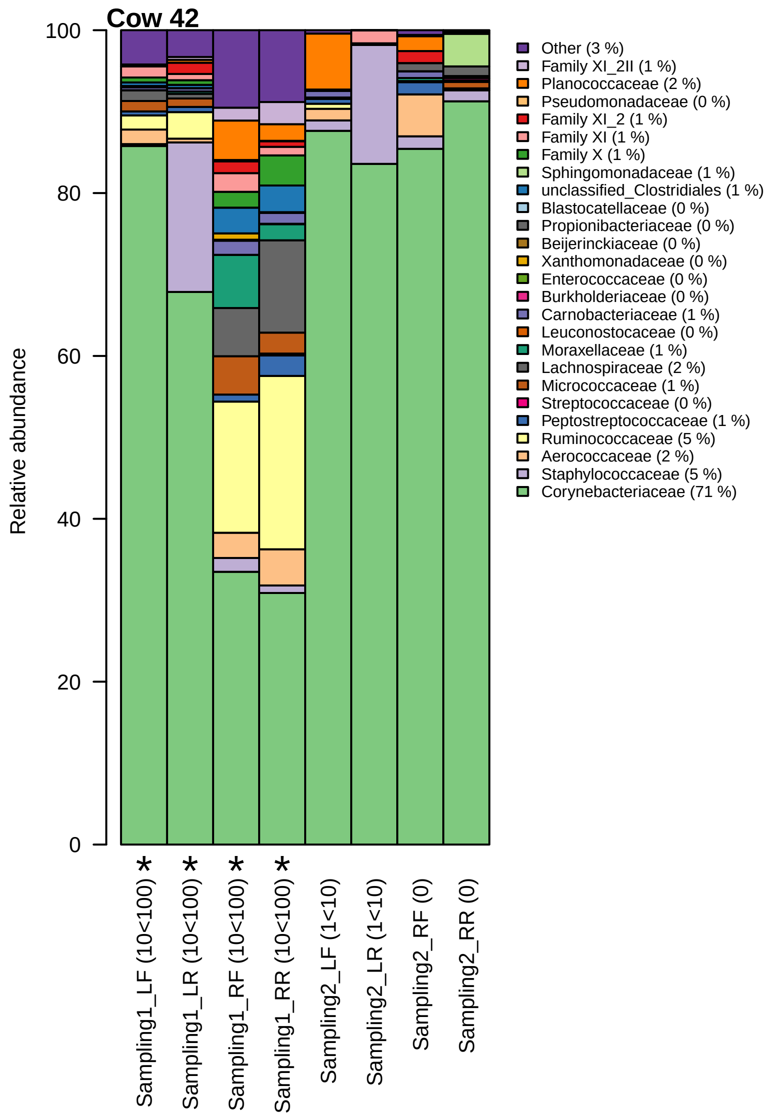

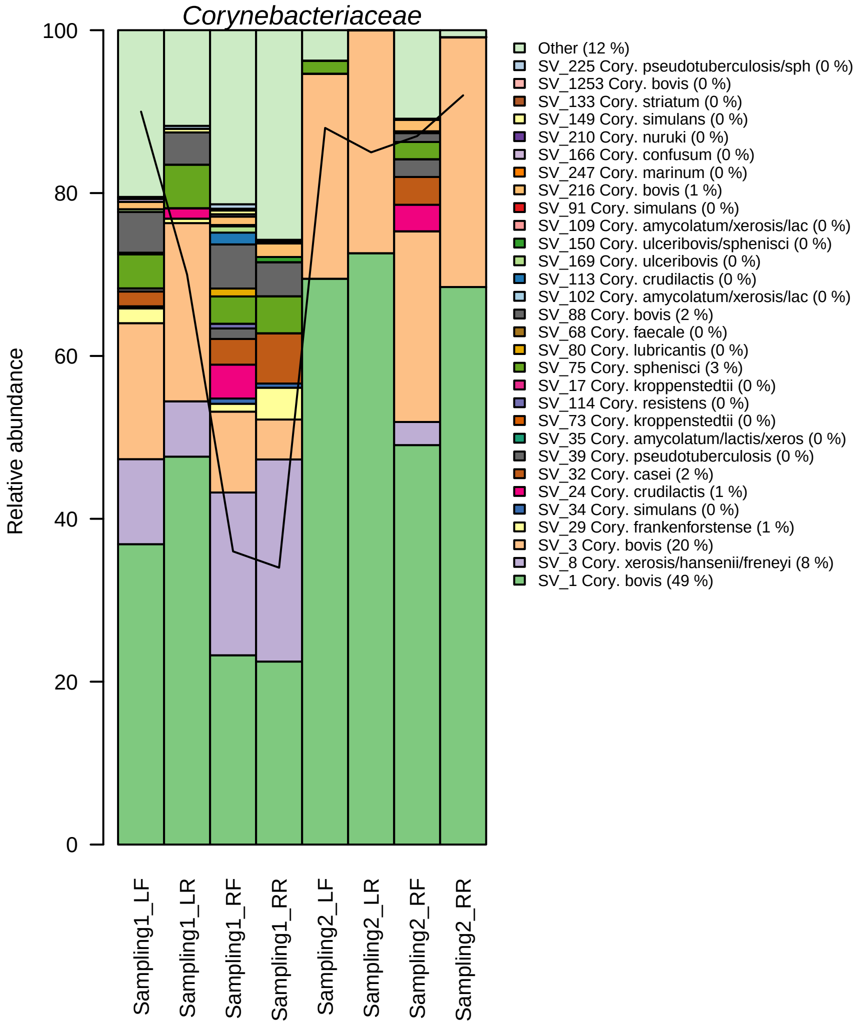

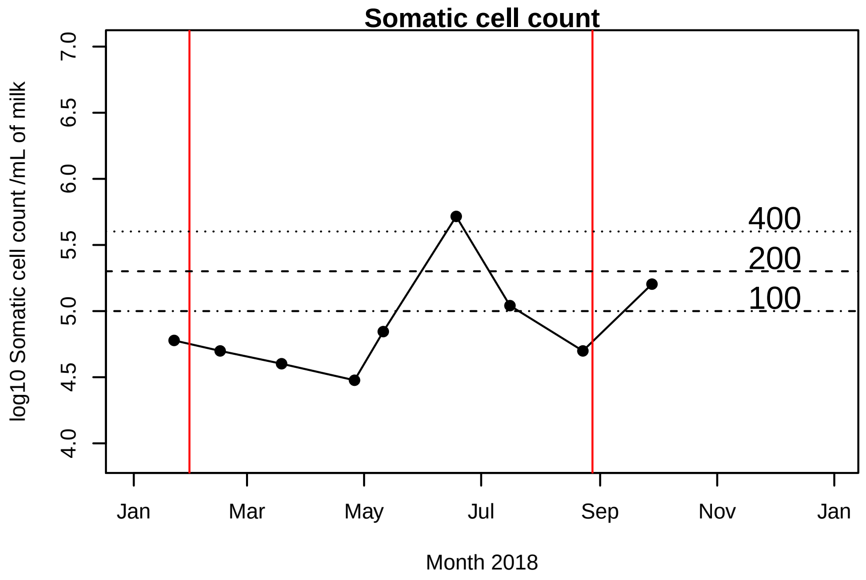

	# quarter	Sampling 1	Sampling 2
**Acinetobacter lwoffii**	1	1	0
**Aerococcus viridans**	5	13	1
**Corynebacterium confusum**	1	1	0
**Corynebacterium stationis**	1	1	0
**Staphylococcus chromogenes**	2	5	1
**Staphylococcus epidermidis**	1	1	0

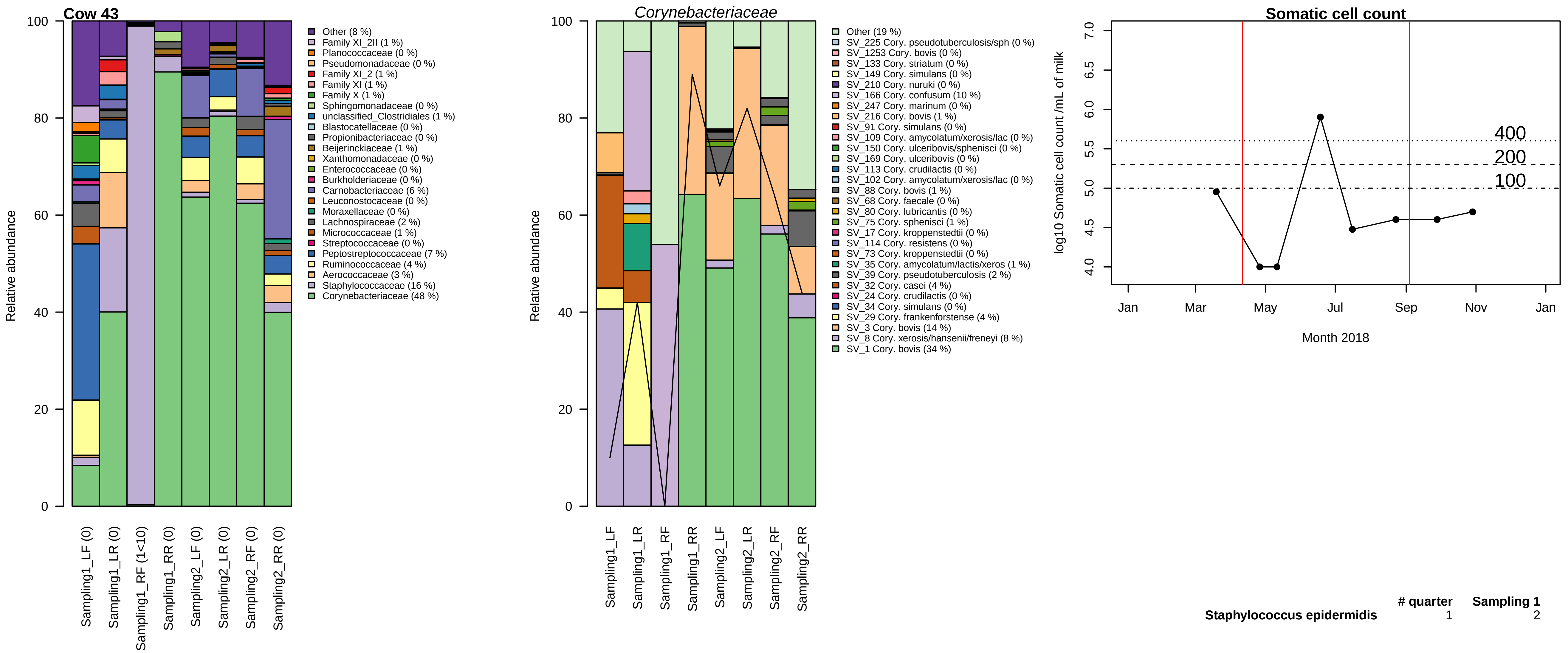

	# quarter	Sampling 1
**Staphylococcus epidermidis**	1	2

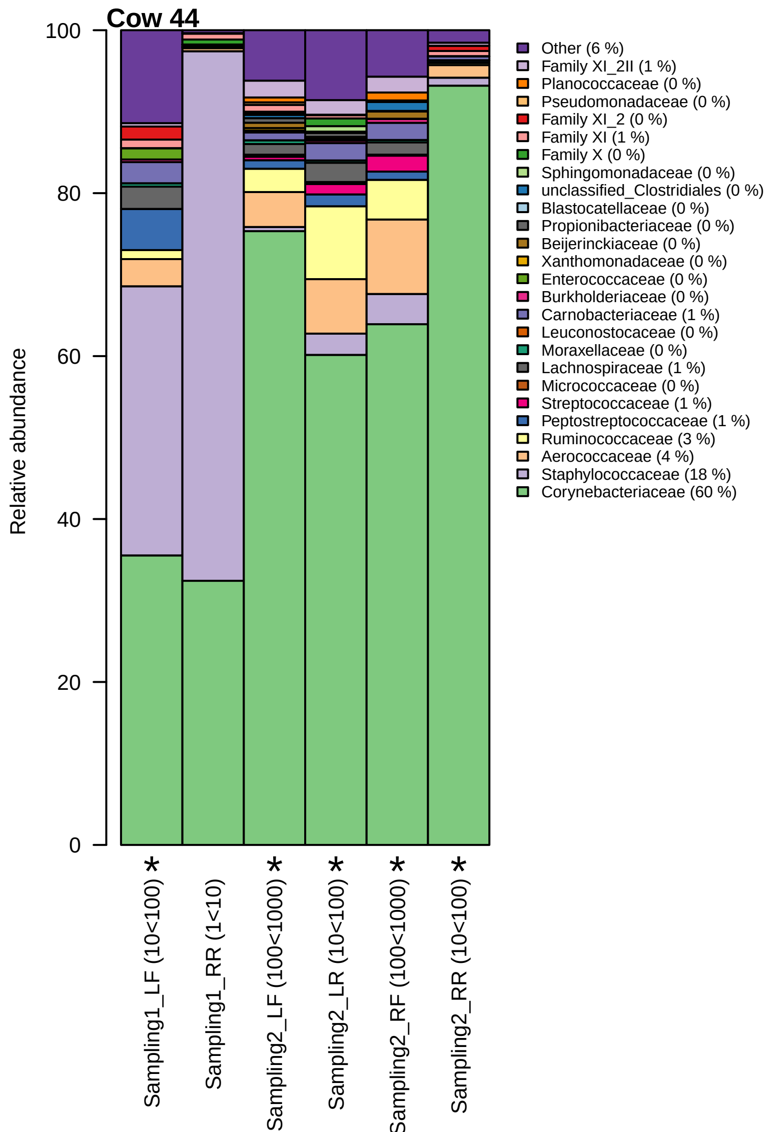

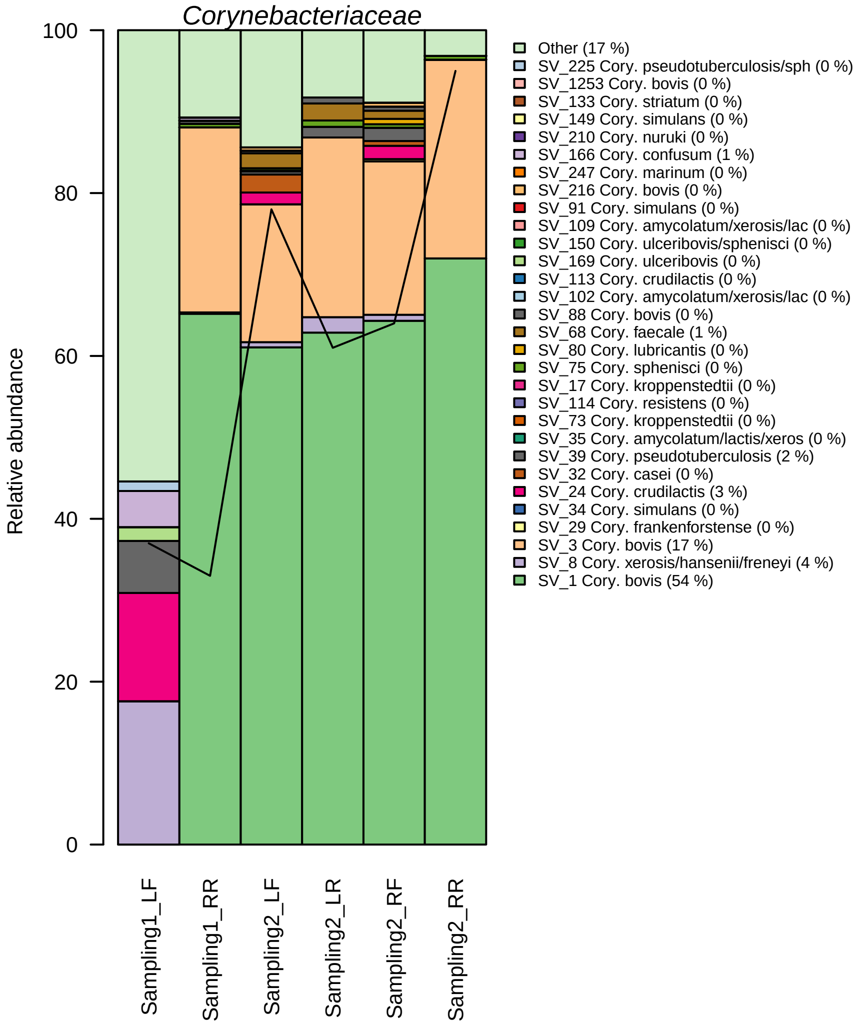

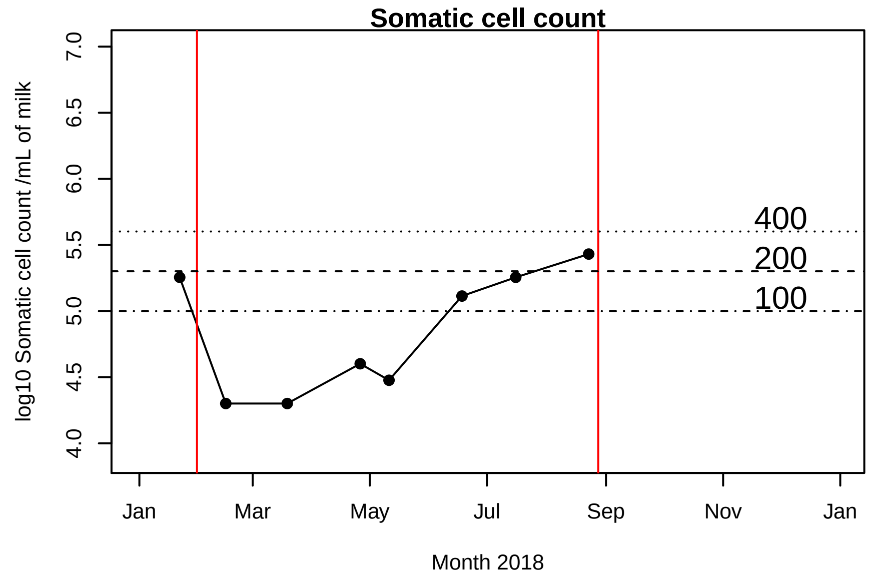

	# quarter	Sampling 1	Sampling 2
**Aerococcus viridans**	6	2	10
**Corynebacterium bovis**	3	0	3
**Corynebacterium confusum**	1	1	0
**Corynebacterium stationis**	1	0	1
**Enterococcus pseudoavium**	1	1	0
**Staphylococcus epidermidis**	4	10	0
**Staphylococcus haemolyticus**	2	0	2
**Staphylococcus saprophyticus**	1	0	1

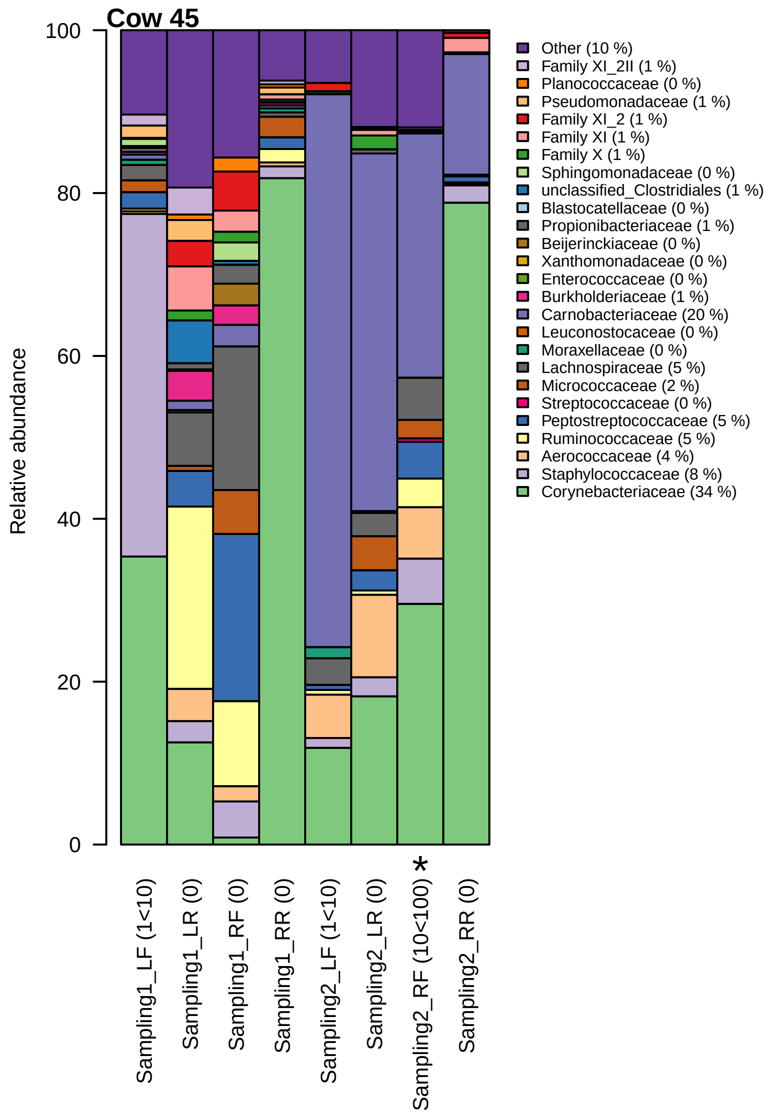

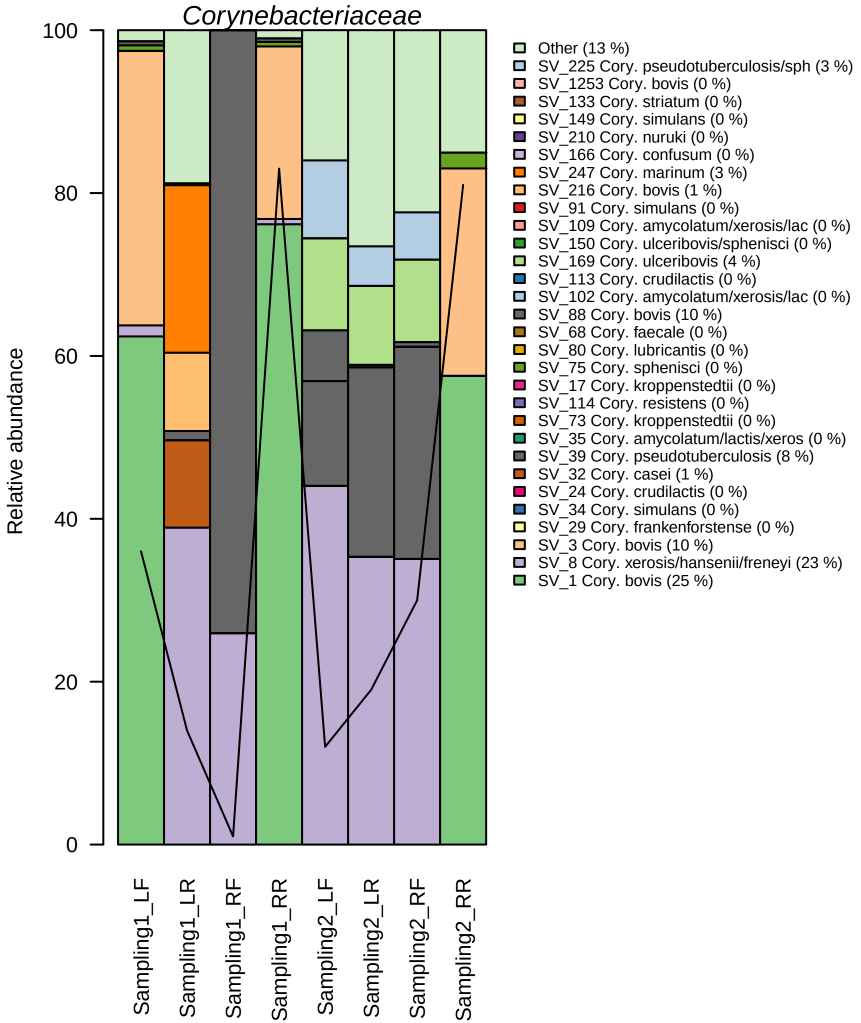

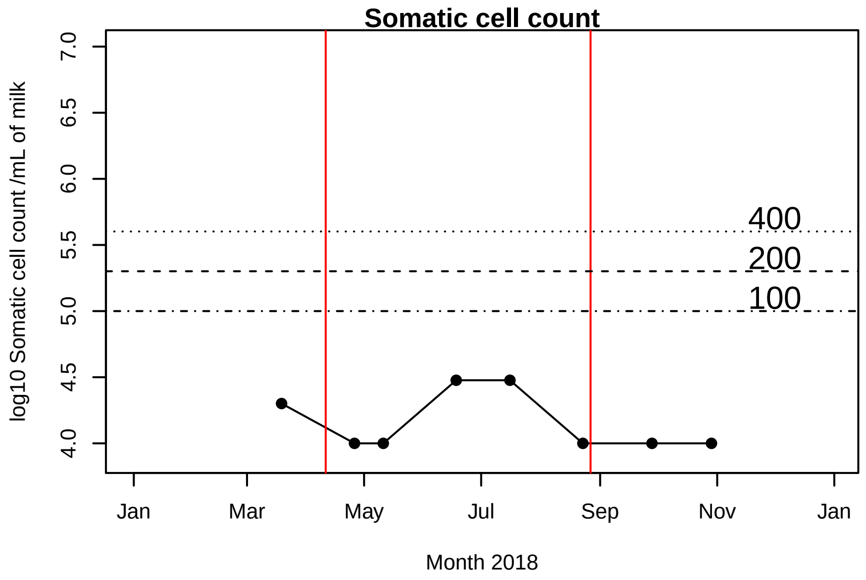

	# quarter	Sampling 1	Sampling 2
**Aerococcus viridans**	2	0	5
**Staphylococcus chromogenes**	1	0	1
**Staphylococcus epidermidis**	1	1	0

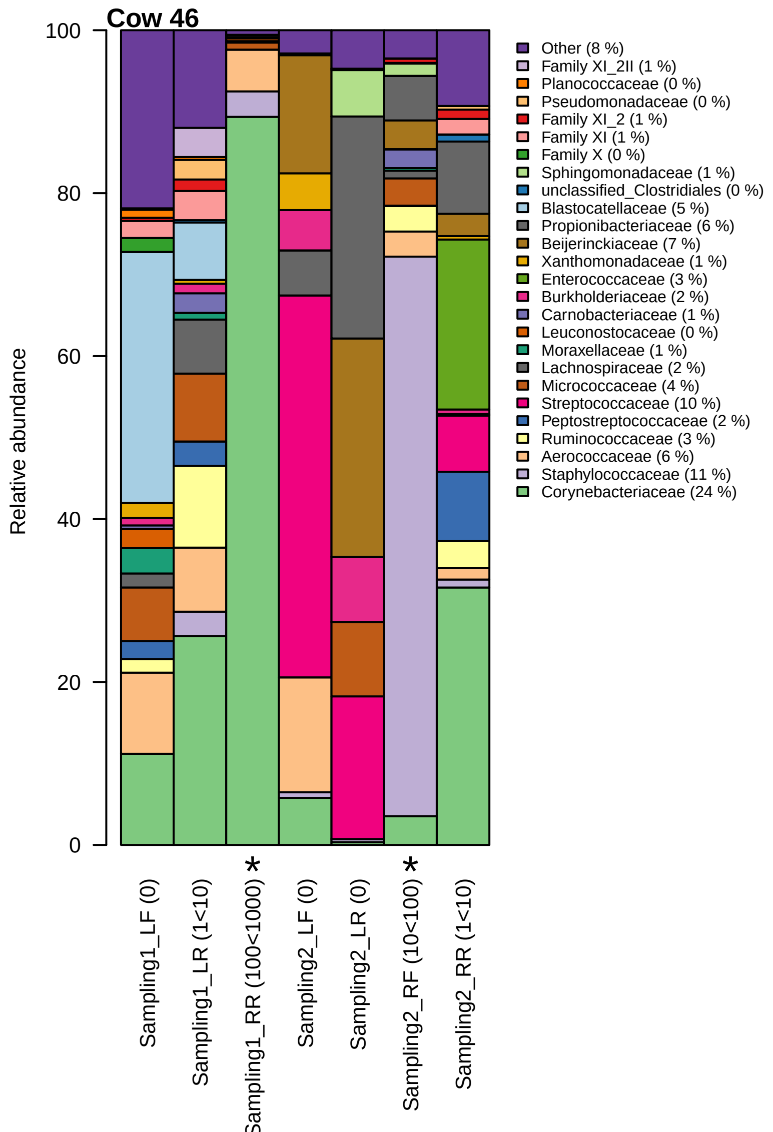

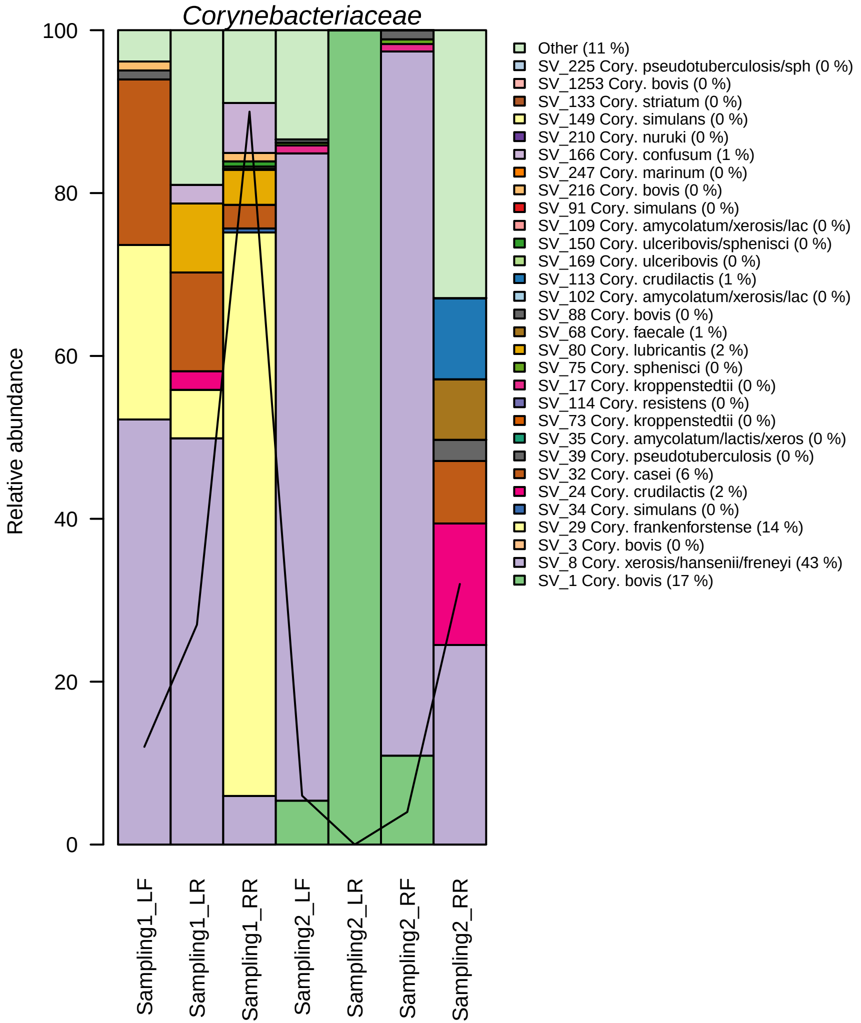

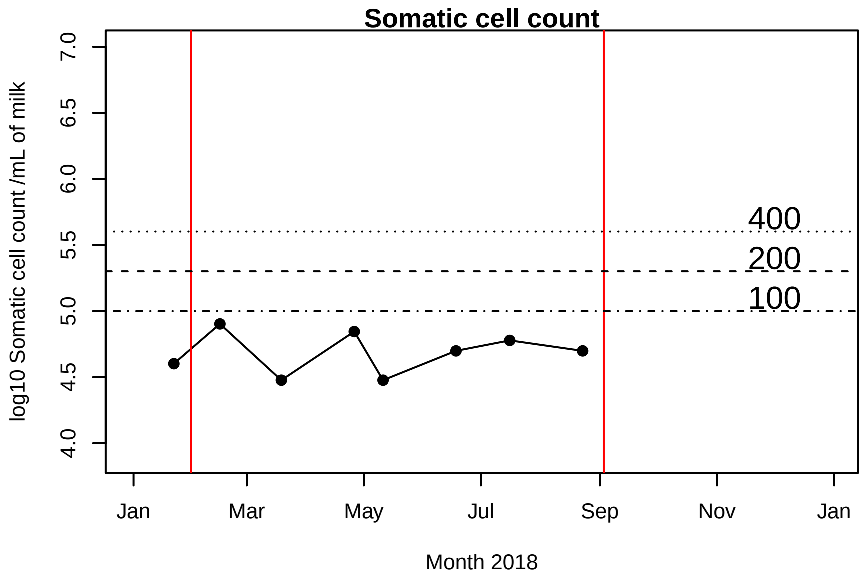

	# quarter	Sampling 1	Sampling 2
**Aerococcus viridans**	3	6	0
**Corynebacterium confusum**	1	1	0
**Corynebacterium stationis**	1	1	0
**Pediococcus pentosaceus**	2	2	0
**Staphylococcus chromogenes**	3	10	3
**Staphylococcus equorum**	1	1	0

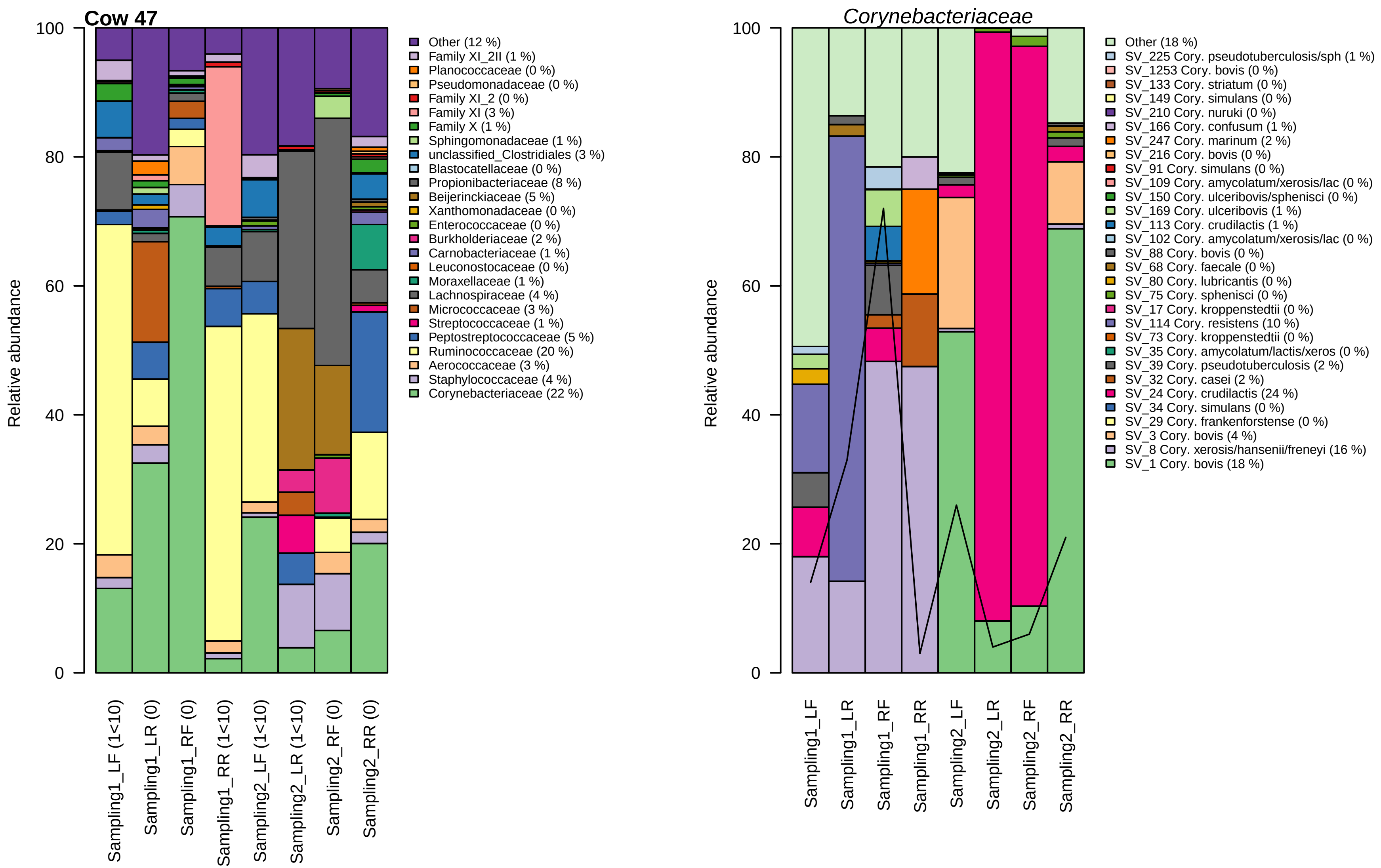

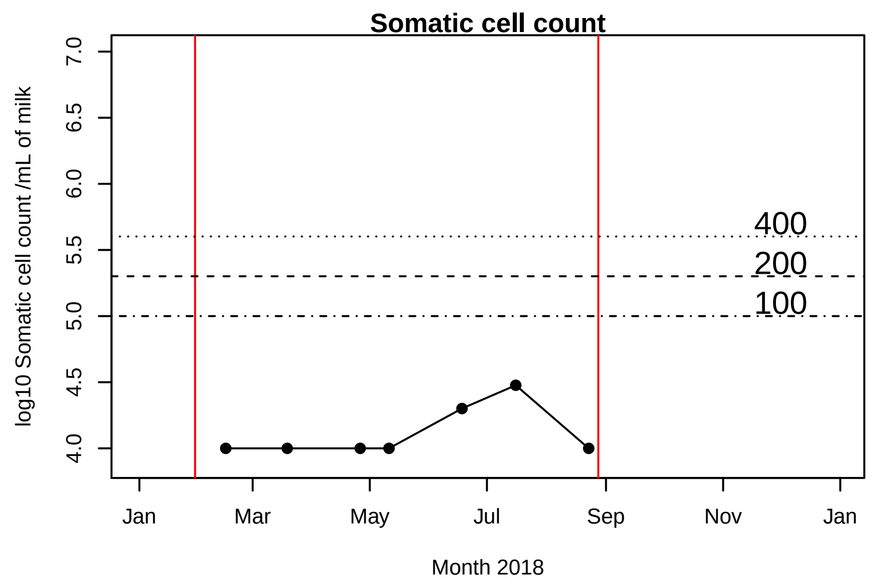

	# quarter	Sampling 1	Sampling 2
**Aerococcus viridans**	1	0	1
**Staphylococcus haemolyticus**	1	1	0

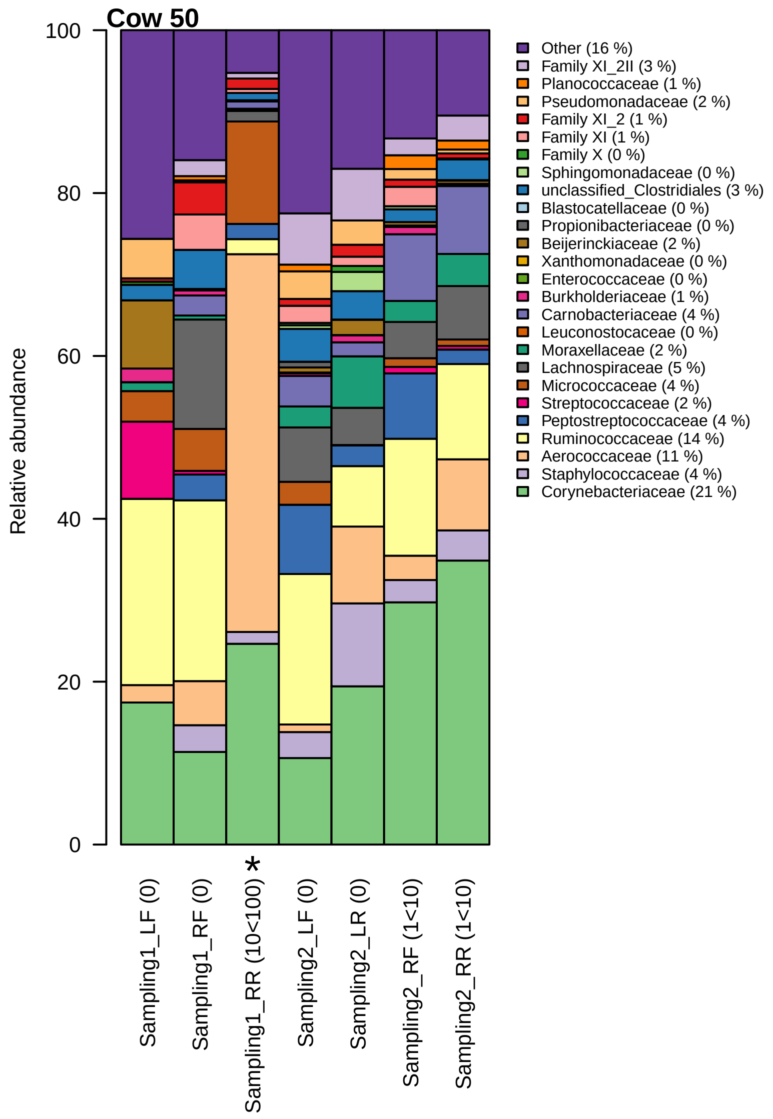

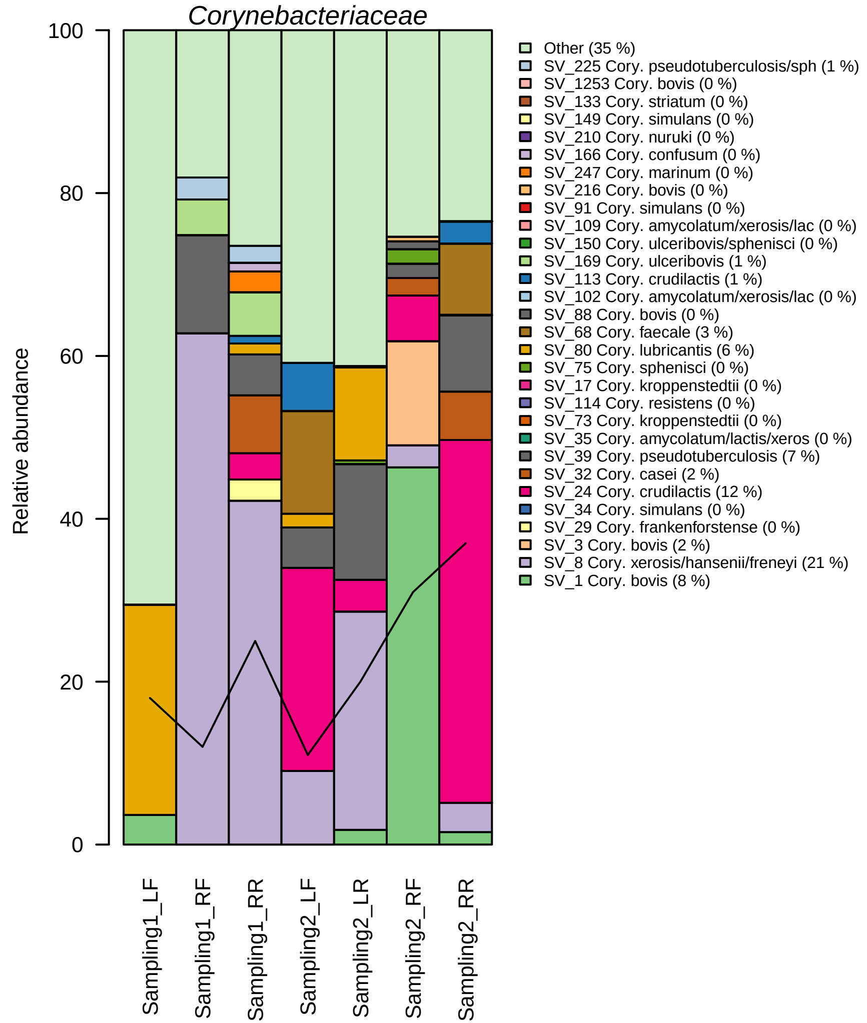

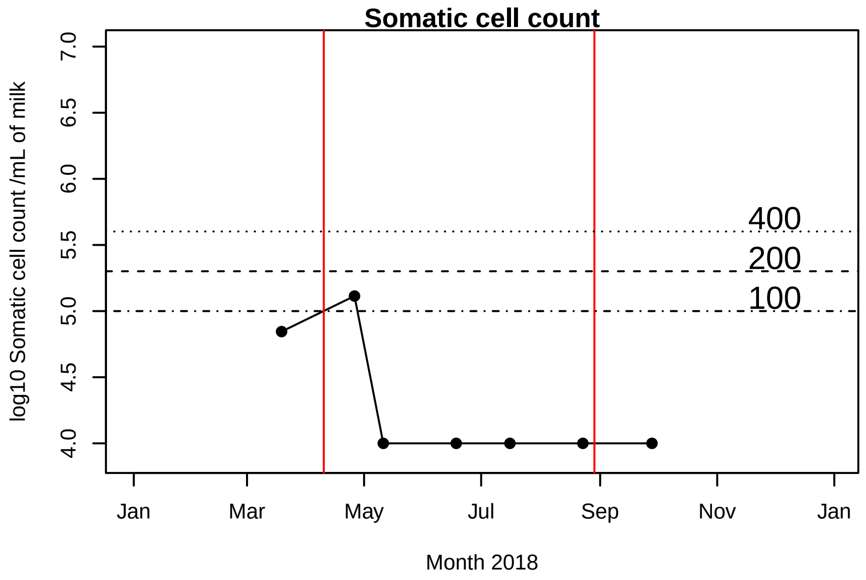

	# quarter	Sampling 1	Sampling 2
**Acinetobacter towneri**	1	0	1
**Aerococcus viridans**	3	8	1
**Pediococcus pentosaceus**	1	1	0
**Staphylococcus auricularis**	1	1	0
**Staphylococcus haemolyticus**	2	1	1

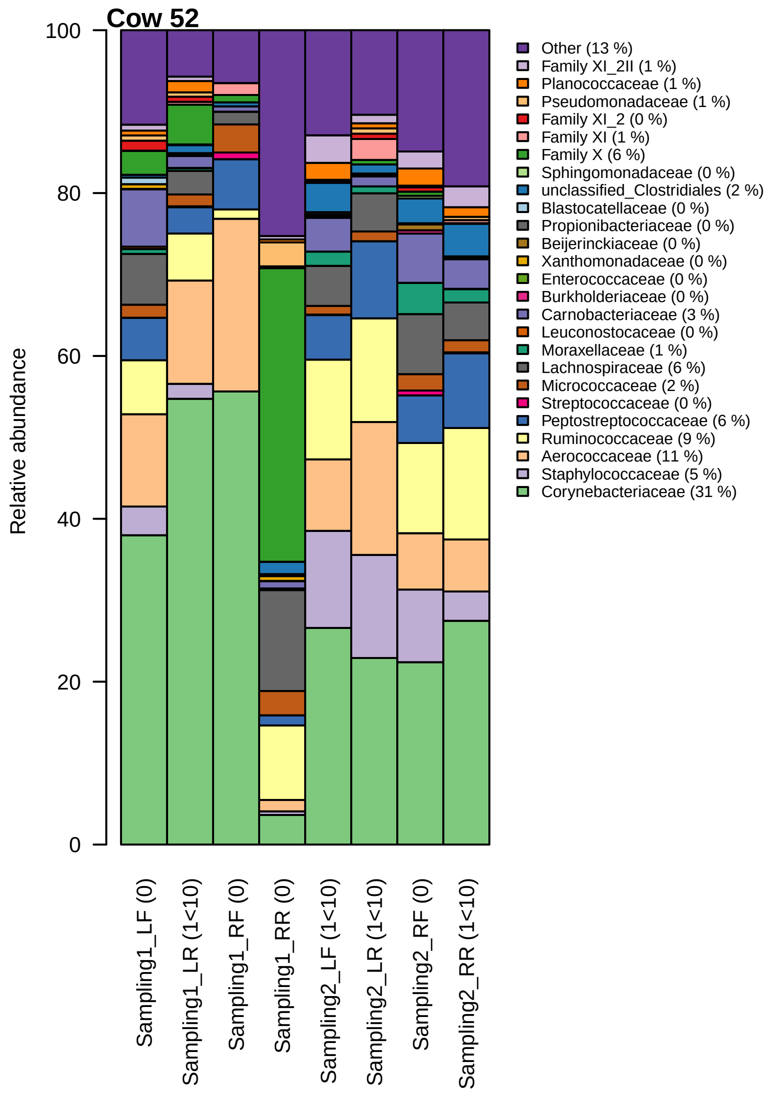

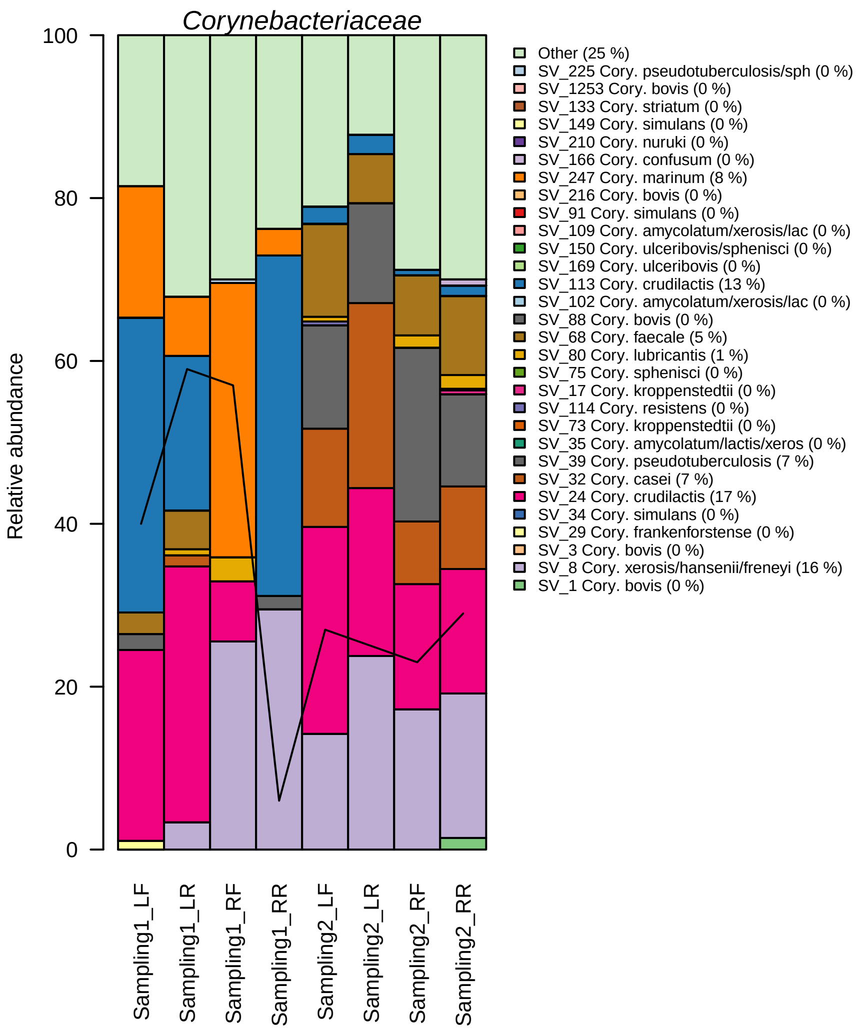

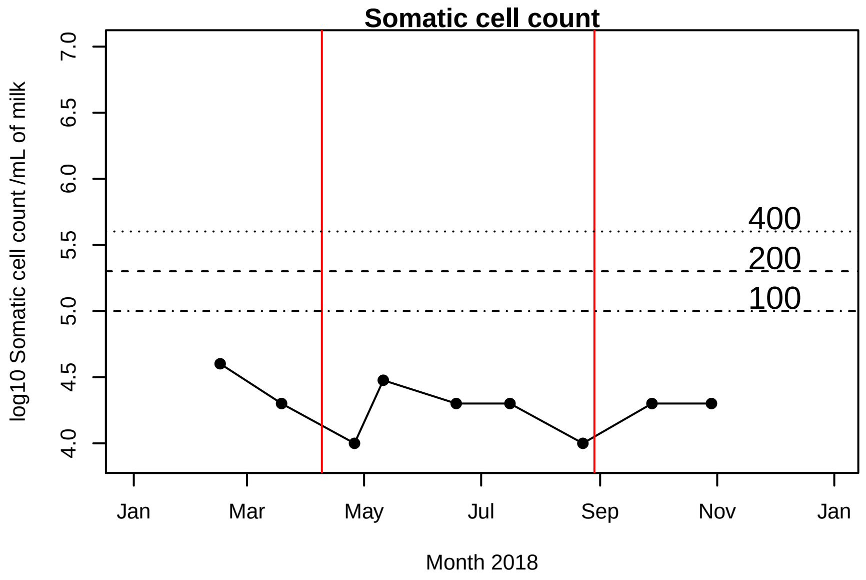

	# quarter	Sampling 1	Sampling 2
**Lysinibacillus sphaericus**	1	1	0
**Staphylococccus chromogenes**	1	0	1
**Staphylococcus capitis**	1	0	1
**Staphylococcus chromogenes**	1	0	1
**Staphylococcus haemolyticus**	1	0	1

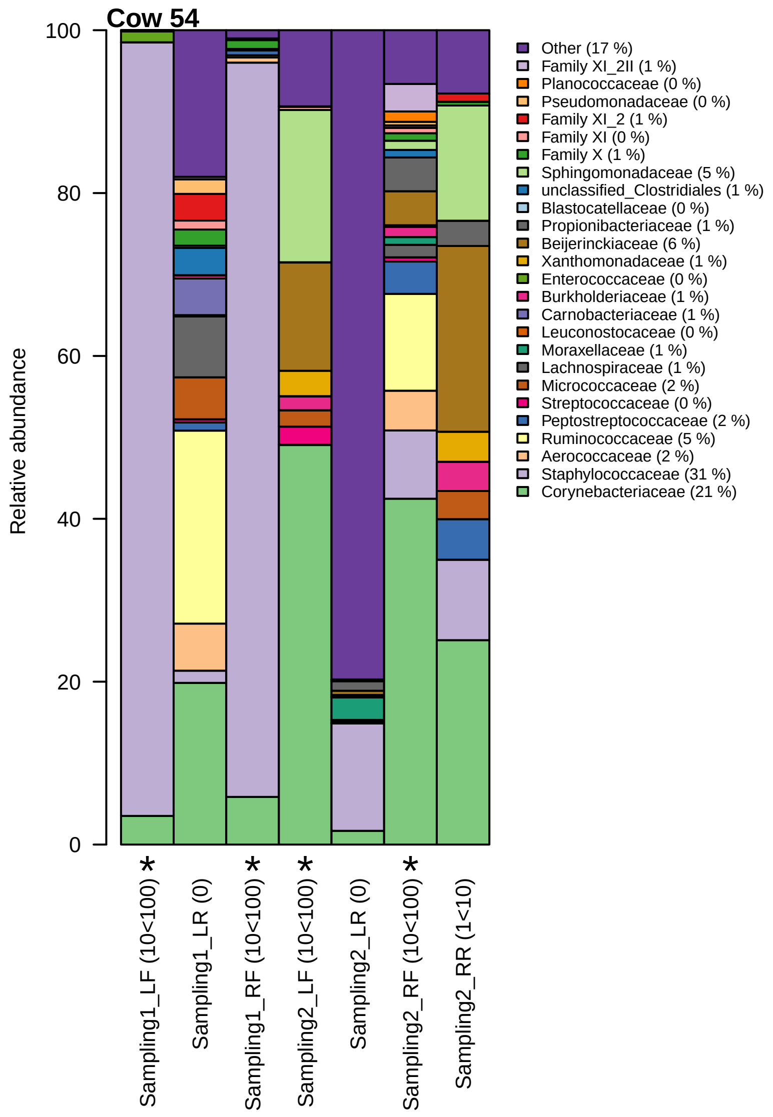

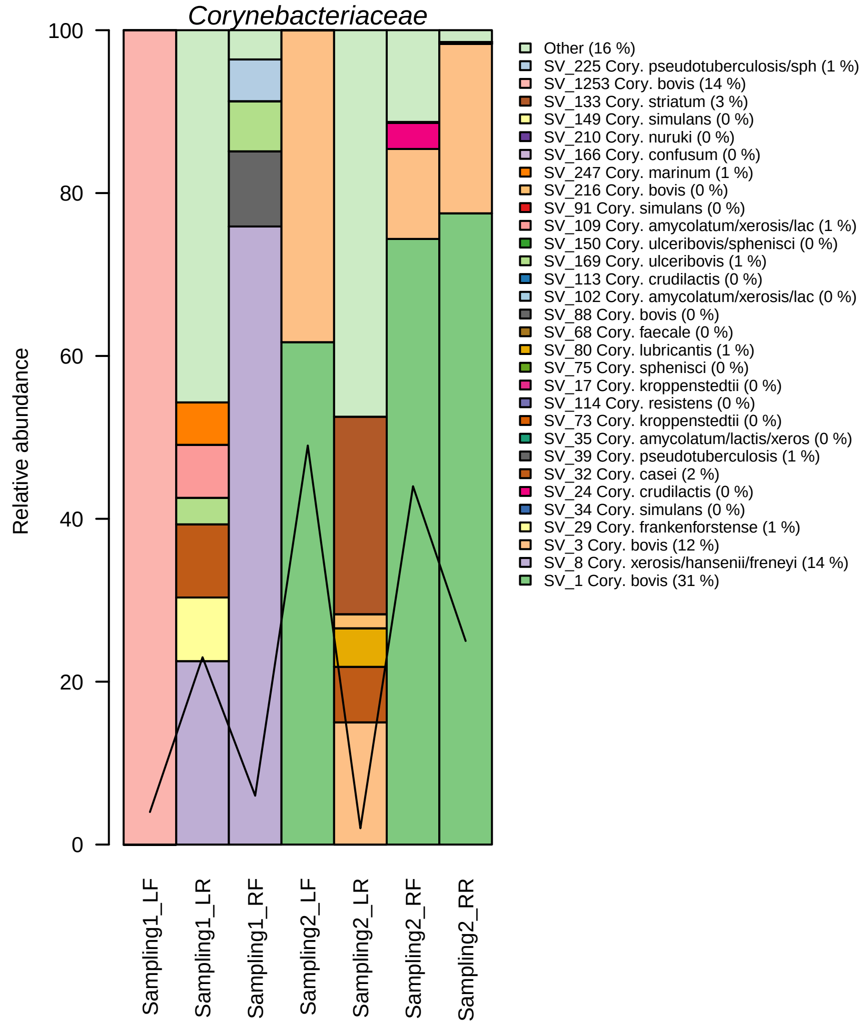

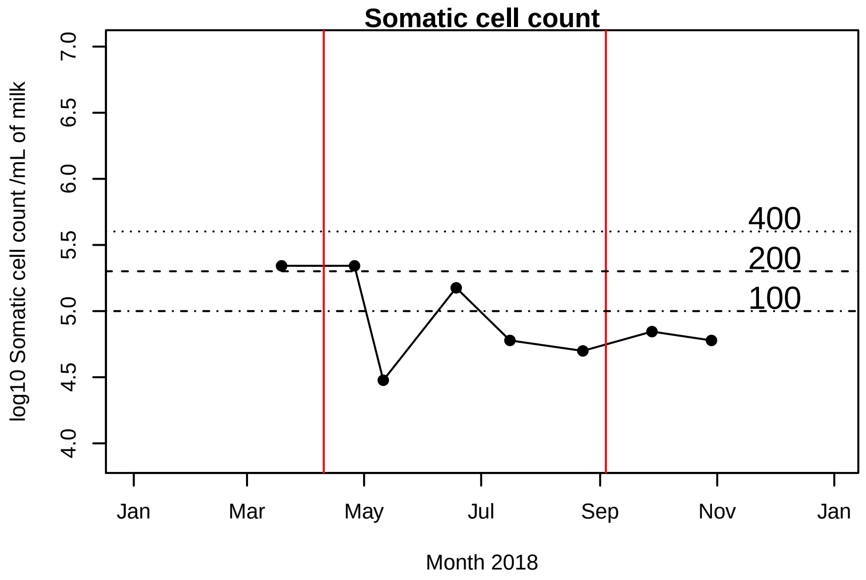

	# quarter	Sampling 1	Sampling 2
**Aerococcus viridans**	1	1	0
**Corynebacterium bovis**	3	0	3
**Staphylococcus chromogenes**	3	15	0
**Staphylococcus epidermidis**	1	0	1
**Streptococcus canis**	1	1	0
**Streptococcus dysgalactiae**	1	2	0

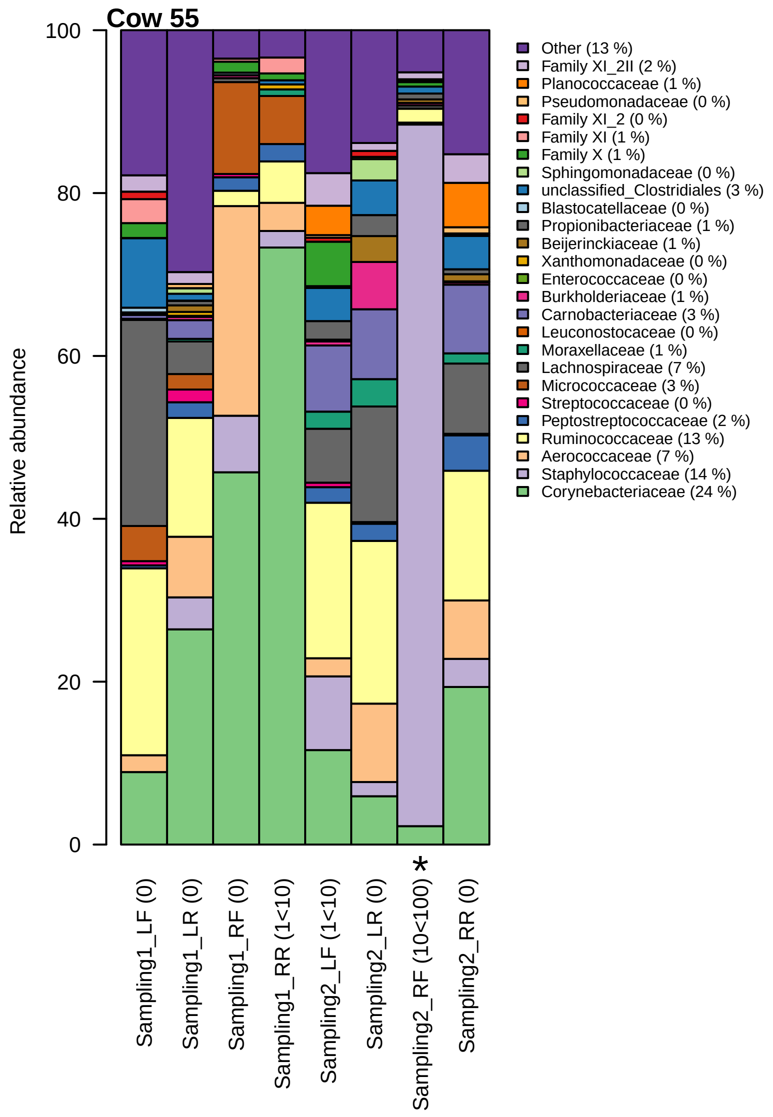

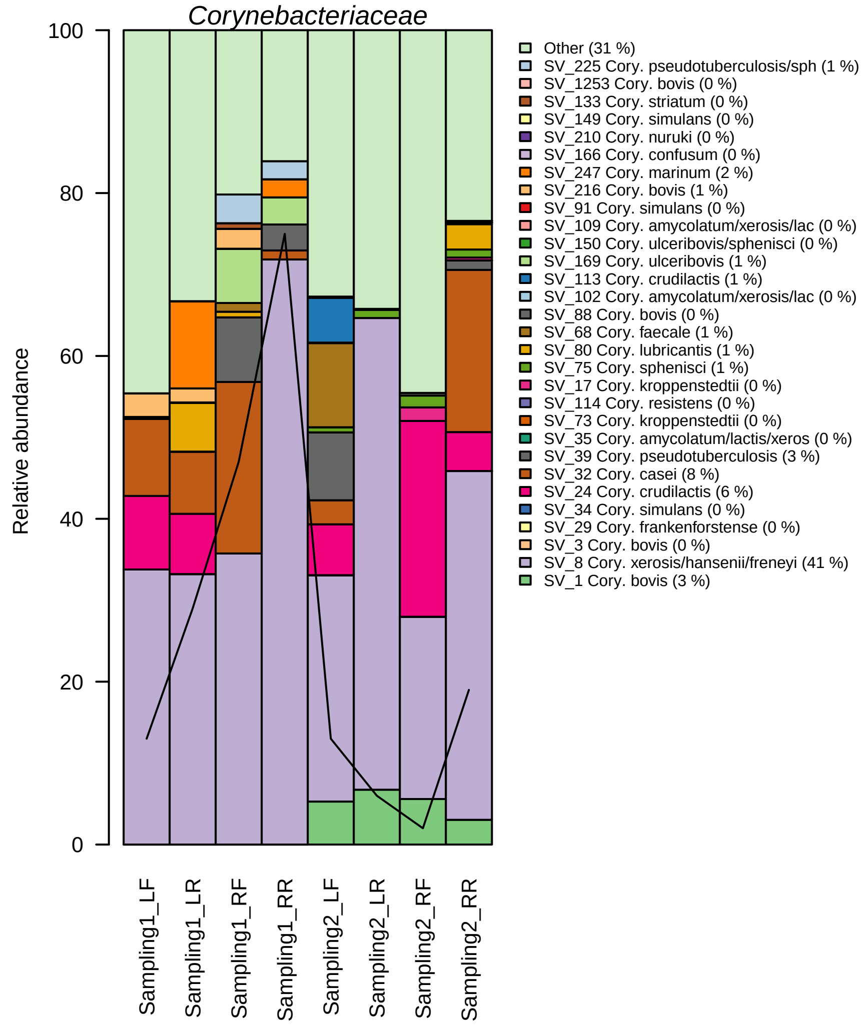

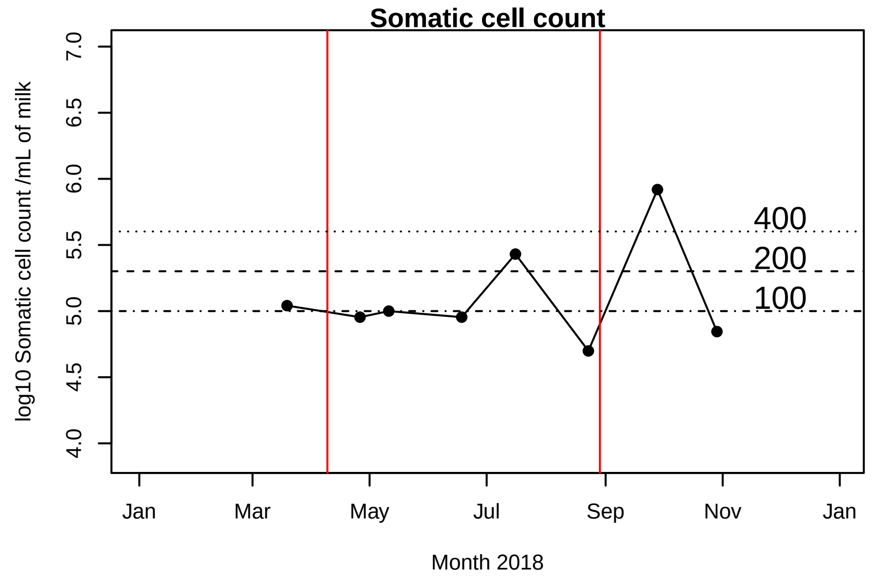

	# quarter	Sampling 1	Sampling 2
**Staphylococcus chromogenes**	3	7	4

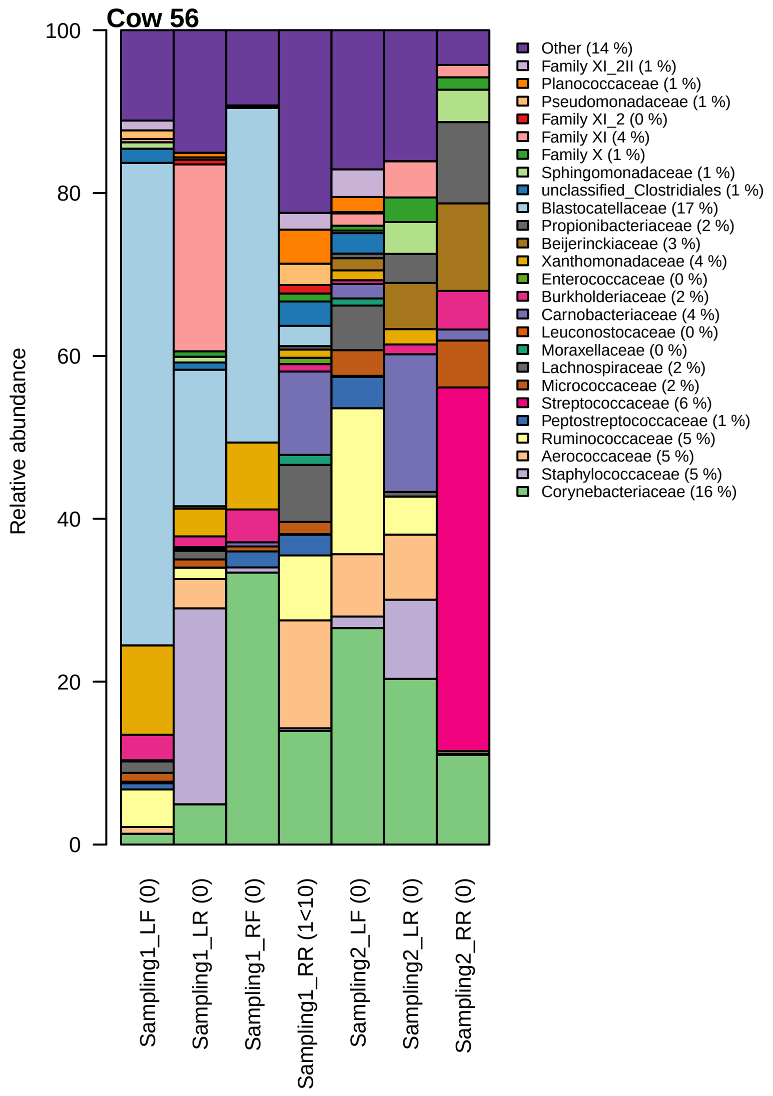

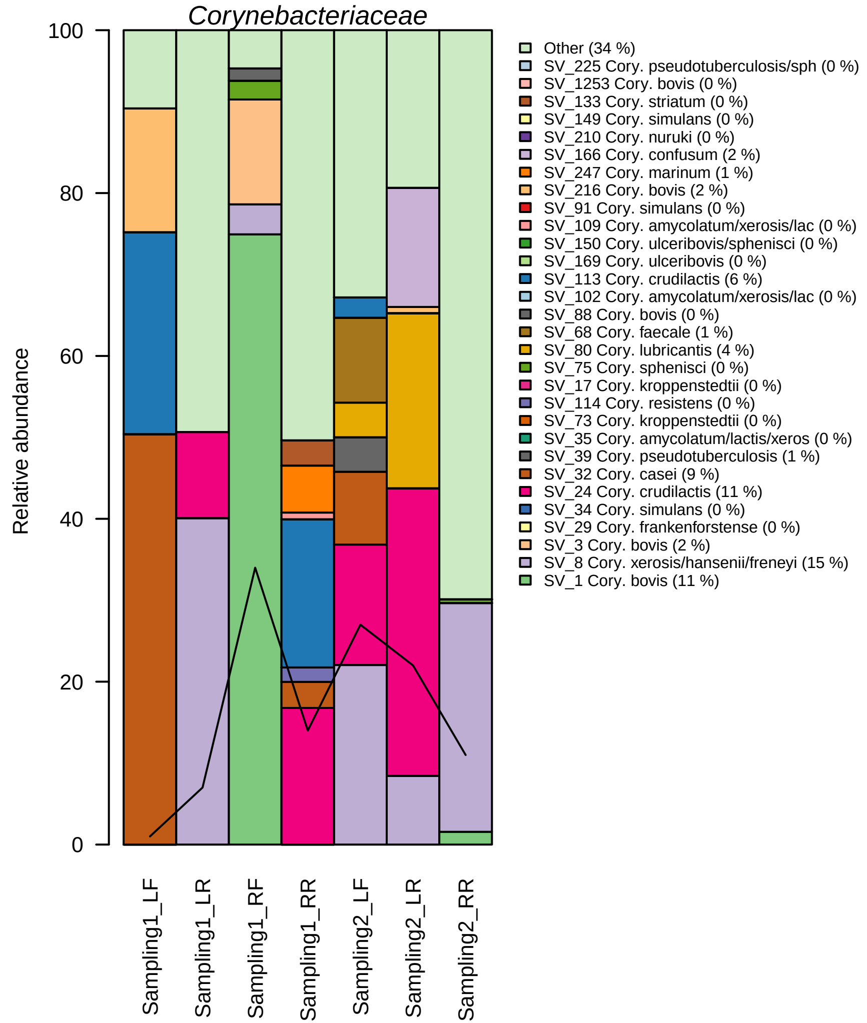

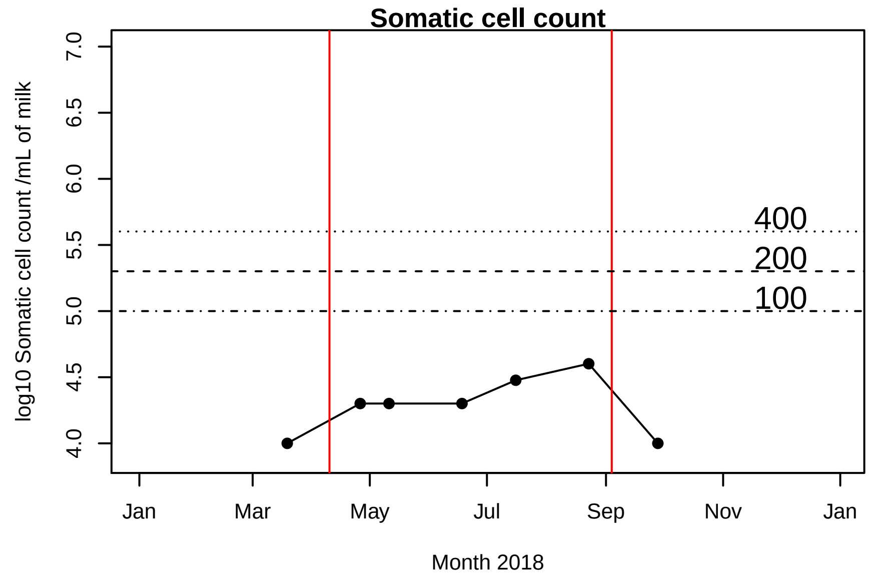

	# quarter	Sampling 1
**Aerococcus viridans**	1	1
**Corynebacterium xerosis**	1	1

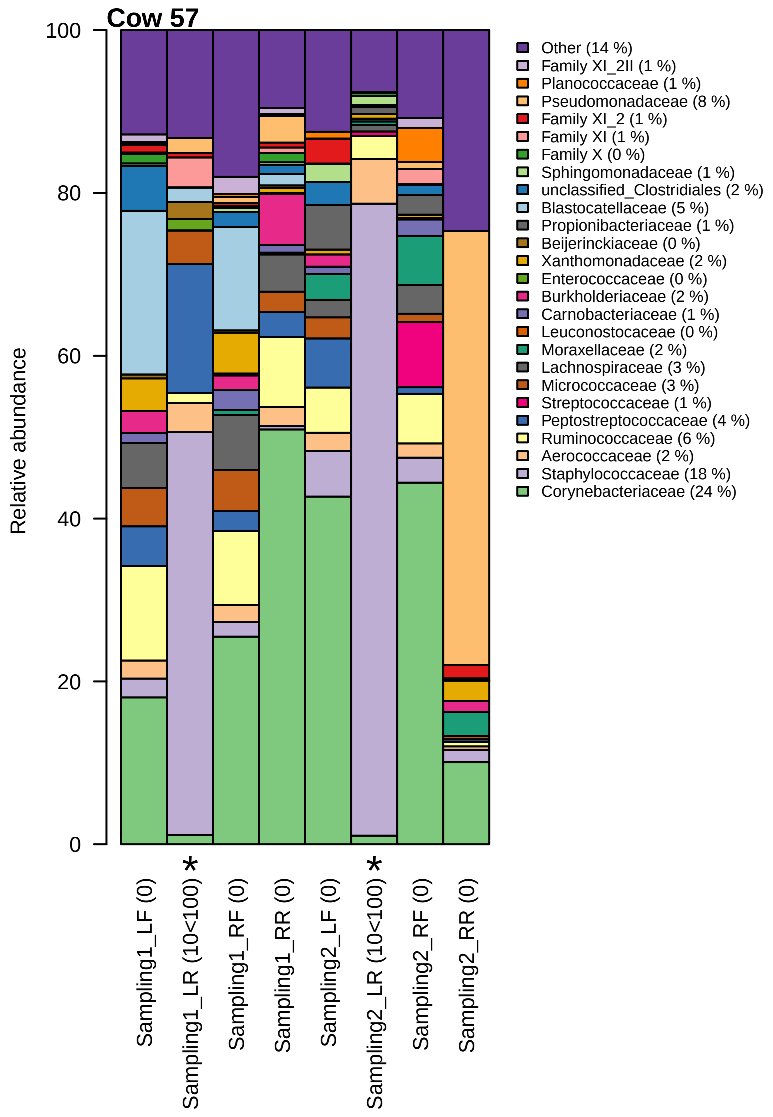

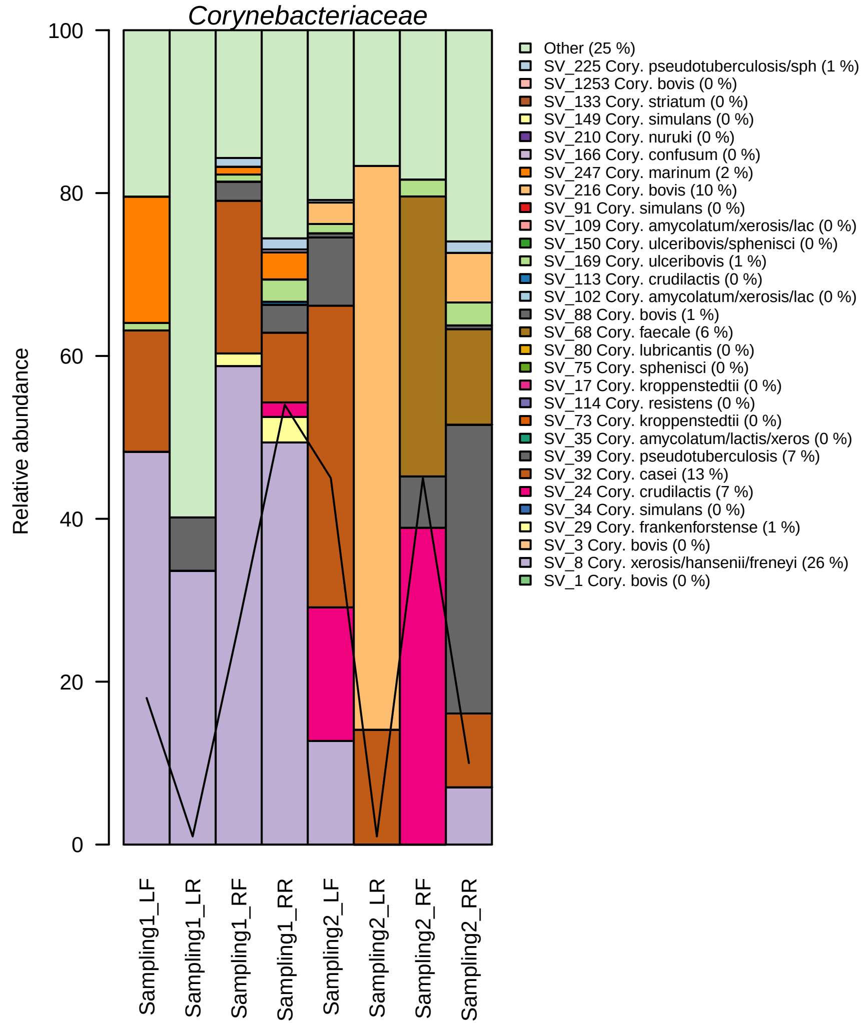

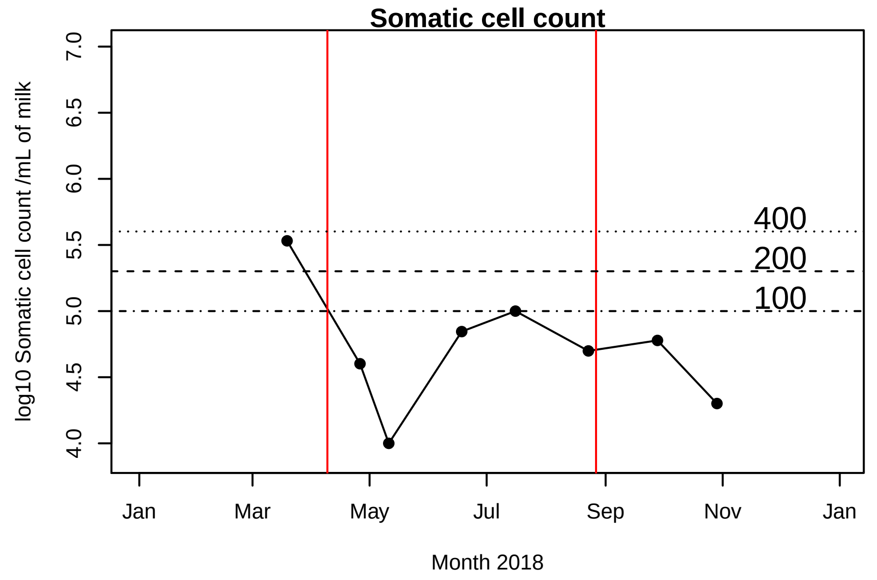

	# quarter	Sampling 1	Sampling 2
**Aerococcus viridans**	1	1	0
**Corynebacterium amycolatum**	1	1	0
**Staphylococcus chromogenes**	2	4	4

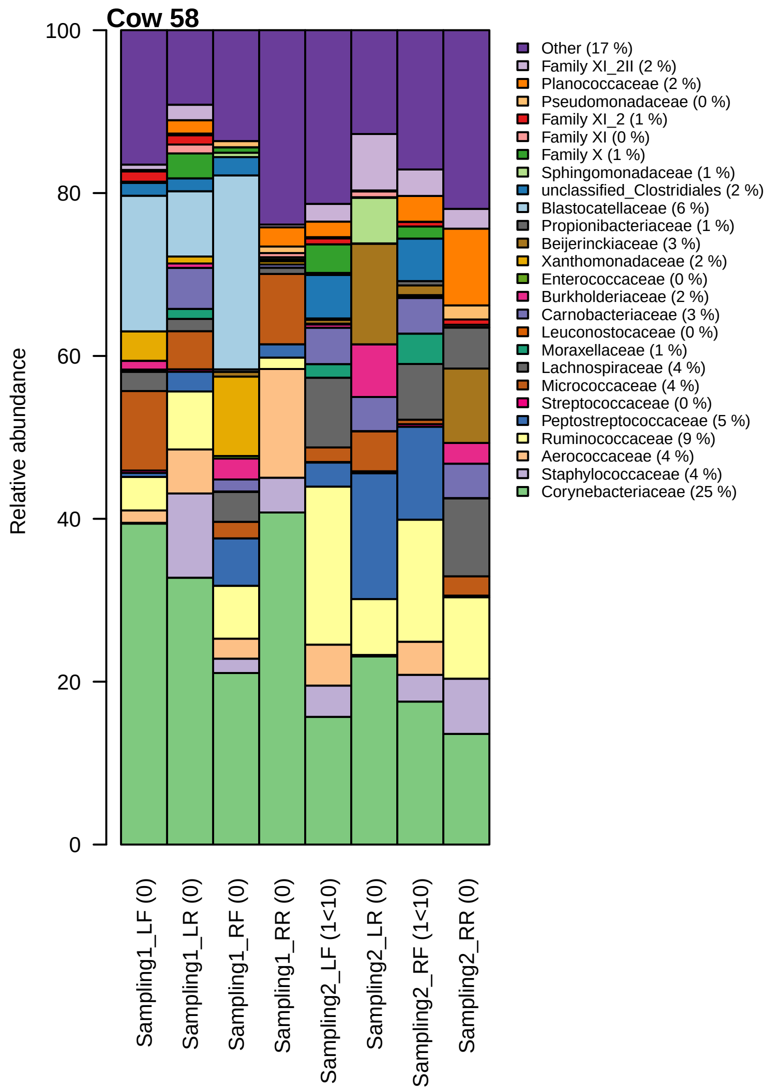

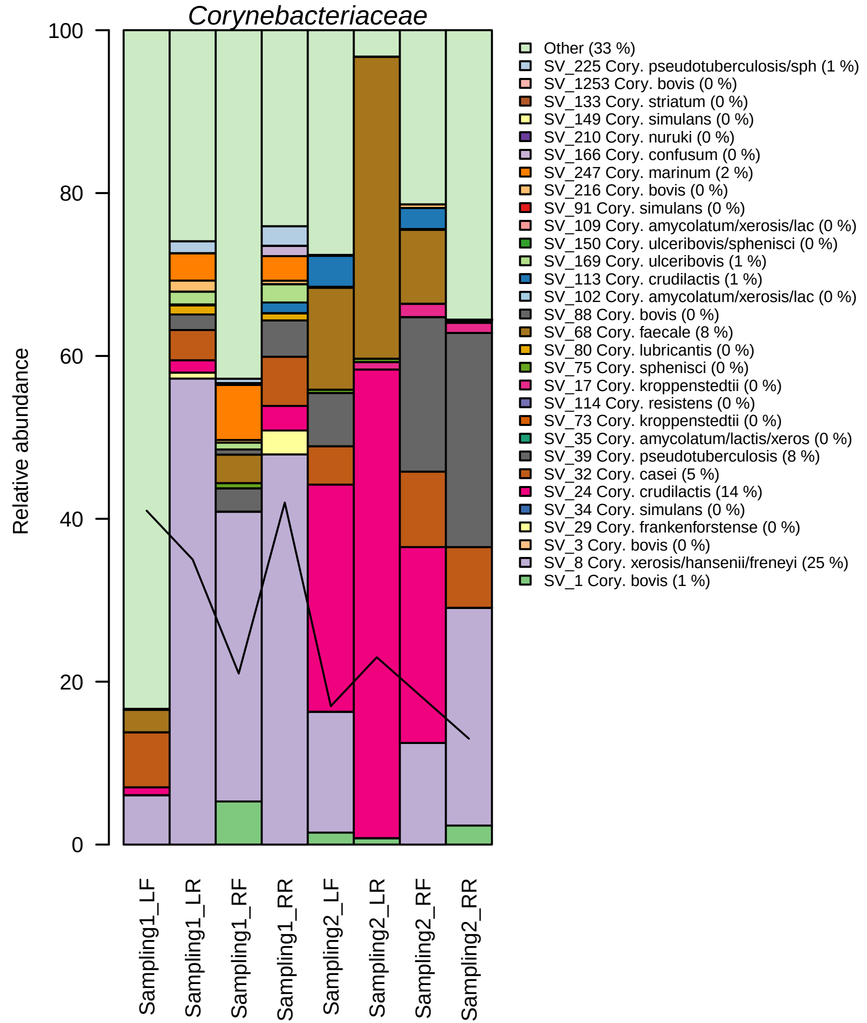

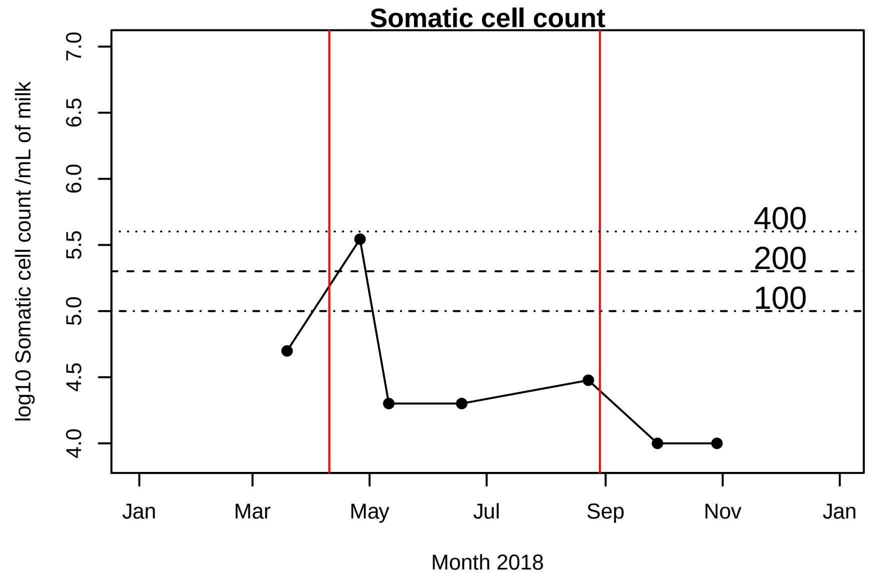

	# quarter	Sampling 2
**Aerococcus viridans**	3	3
**Staphylococcus haemolyticus**	1	1
**Staphylococcus warneri**	1	1

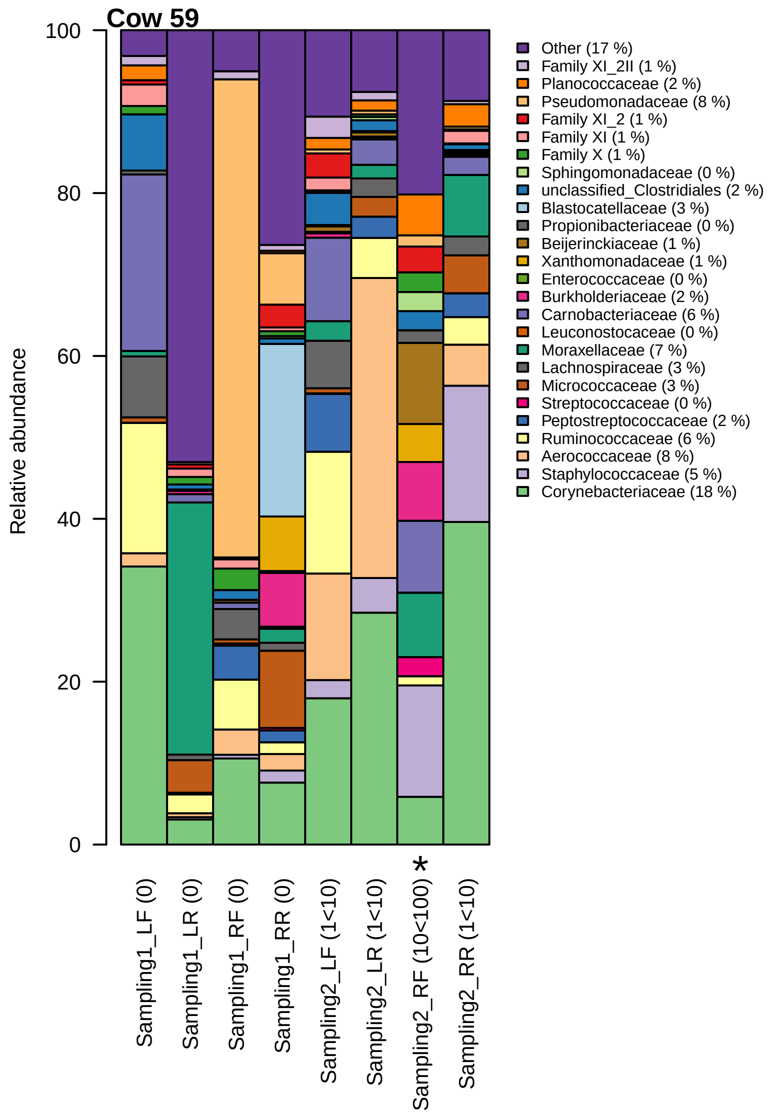

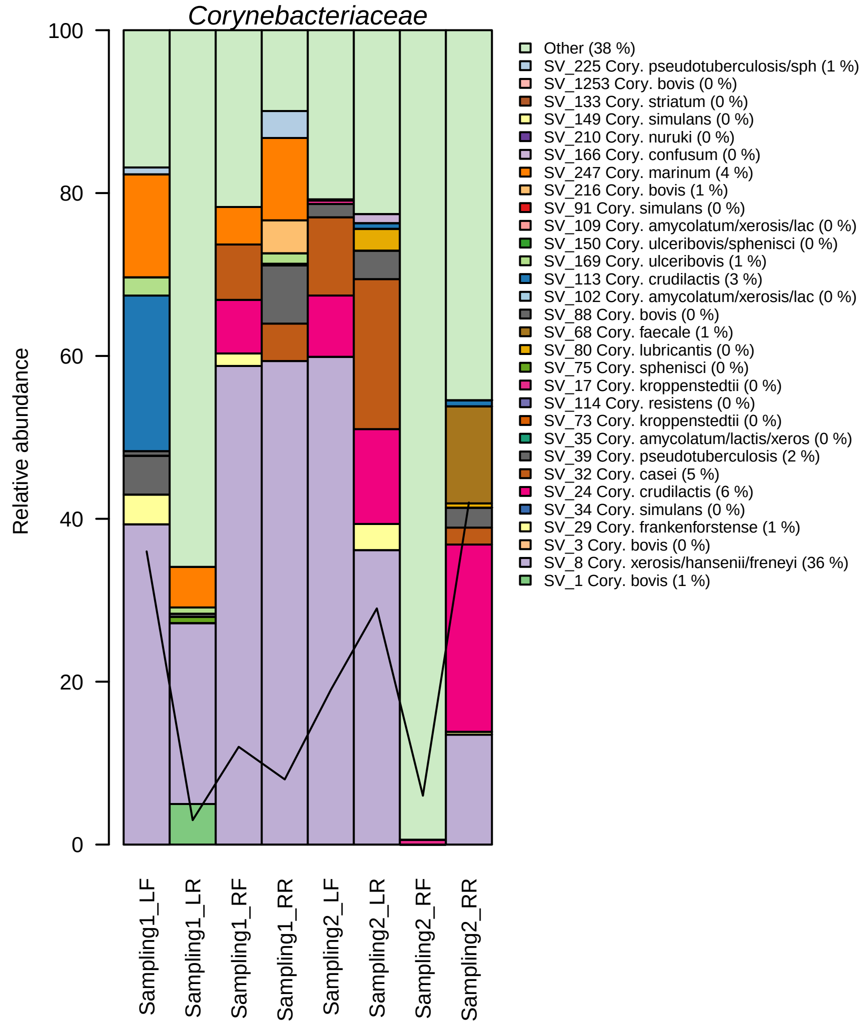

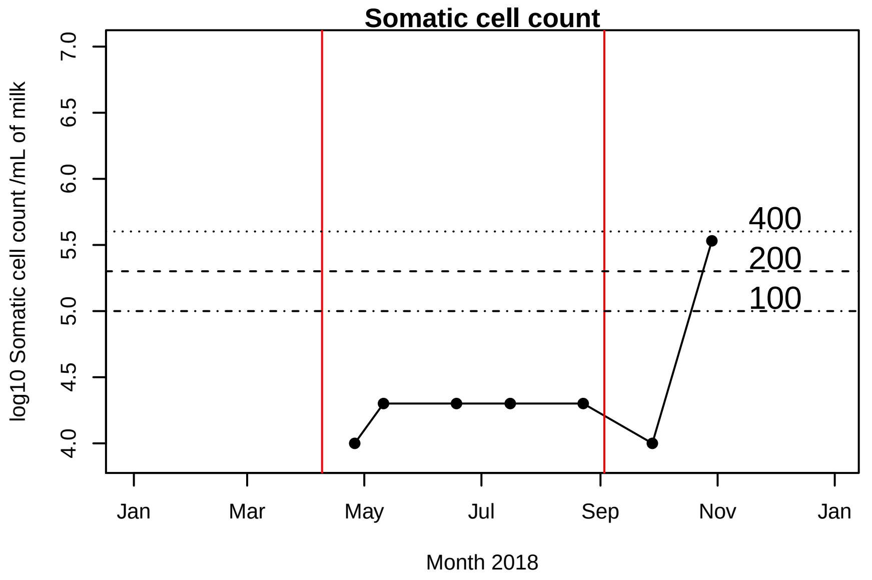

	# quarter	Sampling 2
**Aerococcus viridans**	2	3
**Corynebacterium confusum**	1	1
**Staphylococcus capitis**	1	1

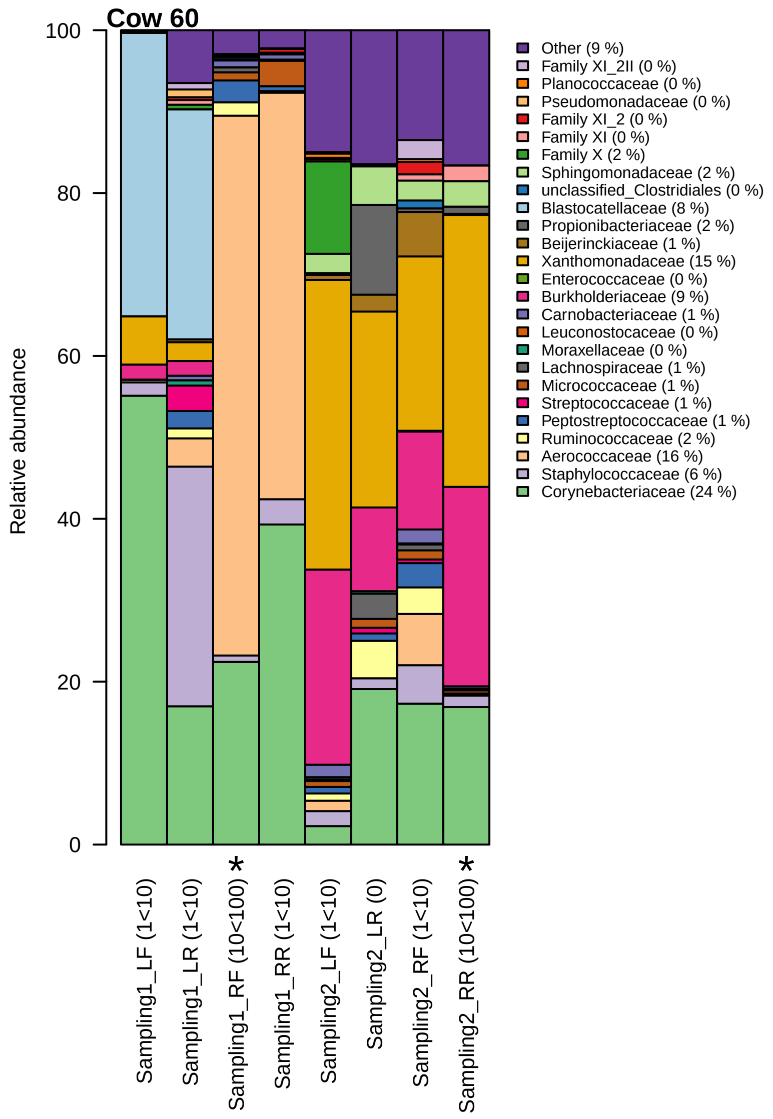

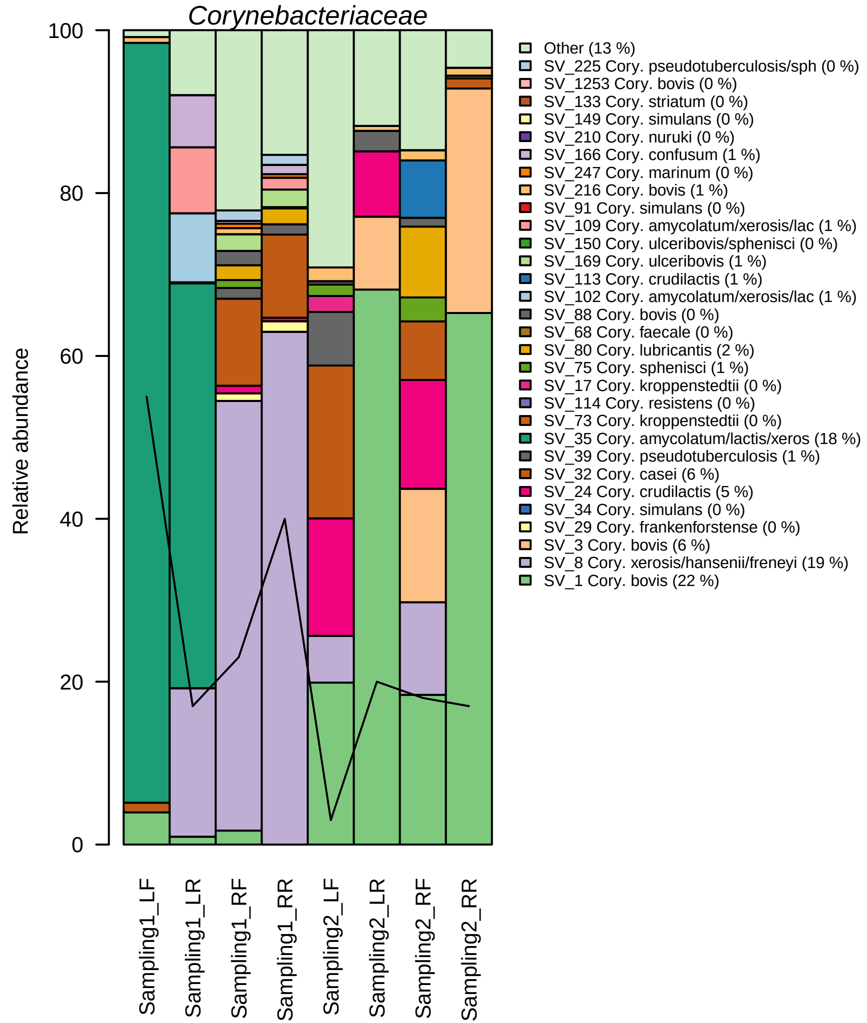

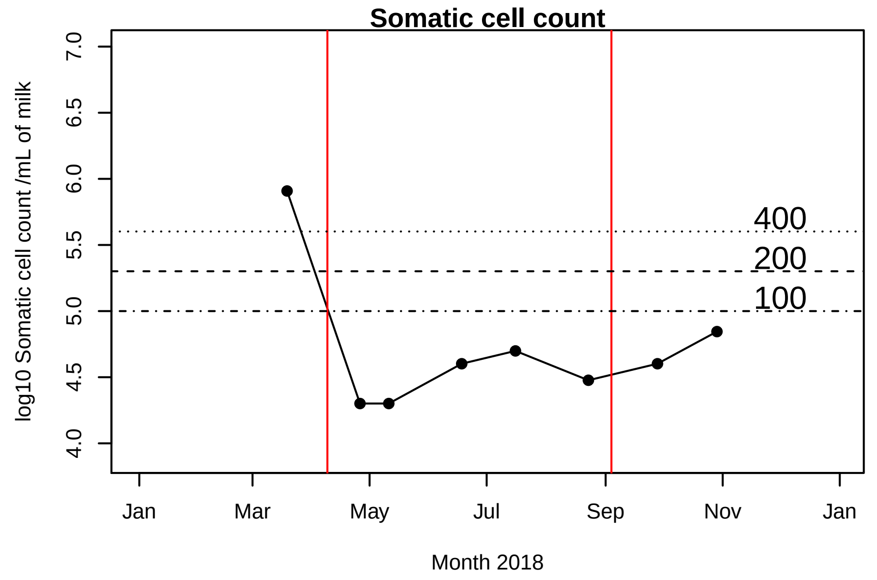

	# quarter	Sampling 1	Sampling 2
**Aerococcus viridans**	3	7	2
**Corynebacterium bovis**	1	0	1
**Corynebacterium confusum**	1	0	1
**Staphylococcus epidermidis**	1	1	0
**Staphylococcus haemolyticus**	1	0	1
**Staphylococcus warneri**	1	2	0
